# A Recalibrated Molecular Clock and Independent Origins for the Cholera Pandemic Clones

**DOI:** 10.1371/journal.pone.0004053

**Published:** 2008-12-30

**Authors:** Lu Feng, Peter R. Reeves, Ruiting Lan, Yi Ren, Chunxu Gao, Zhemin Zhou, Yan Ren, Jiansong Cheng, Wei Wang, Jianmei Wang, Wubin Qian, Dan Li, Lei Wang

**Affiliations:** 1 TEDA School of Biological Sciences and Biotechnology Nankai University, Tianjin, China; 2 Tianjin Research Center for Functional Genomics and Biochip, Tianjin Economic-Technological Development Area (TEDA), Tianjin, China; 3 School of Molecular and Microbial Biosciences, University of Sydney, Sydney, New South Wales, Australia; 4 School of Biotechnology and Biomolecular Sciences, University of New South Wales, Sydney, New South Wales, Australia; 5 Tianjin Key Laboratory of Microbial Functional Genomics, Nankai University, Tianjin Economic-Technological Development Area (TEDA), Tianjin, China; Institut Pasteur, France

## Abstract

Cholera, caused by *Vibrio cholerae*, erupted globally from South Asia in 7 pandemics, but there were also local outbreaks between the 6^th^ (1899–1923) and 7^th^ (1961–present) pandemics. All the above are serotype O1, whereas environmental or invertebrate isolates are antigenically diverse. The pre 7th pandemic isolates mentioned above, and other minor pathogenic clones, are related to the 7^th^ pandemic clone, while the 6^th^ pandemic clone is in the same lineage but more distantly related, and non-pathogenic isolates show no clonal structure. To understand the origins and relationships of the pandemic clones, we sequenced the genomes of a 1937 prepandemic strain and a 6^th^ pandemic isolate, and compared them with the published 7^th^ pandemic genome. We distinguished mutational and recombinational events, and allocated these and other events, to specific branches in the evolutionary tree. There were more mutational than recombinational events, but more genes, and 44 times more base pairs, changed by recombination. We used the mutational single-nucleotide polymorphisms and known isolation dates of the prepandemic and 7^th^ pandemic isolates to estimate the mutation rate, and found it to be 100 fold higher than usually assumed. We then used this to estimate the divergence date of the 6^th^ and 7^th^ pandemic clones to be about 1880. While there is a large margin of error, this is far more realistic than the 10,000–50,000 years ago estimated using the usual assumptions. We conclude that the 2 pandemic clones gained pandemic potential independently, and overall there were 29 insertions or deletions of one or more genes. There were also substantial changes in the major integron, attributed to gain of individual cassettes including copying from within, or loss of blocks of cassettes. The approaches used open up new avenues for analysing the origin and history of other important pathogens.

## Introduction

Cholera was recognised in accounts from ancient India and China [1], but it was only in 1817 that an epidemic in India spread westwards to become the first of 7 pandemics. The etiological agent of pandemic cholera was the bacterium *Vibrio cholerae* serotype O1, which was later divided into two biotypes: classical biotype and El Tor biotype. The first six pandemics were caused by the classical biotype, but in the early 1900s a new form of the species emerged that caused mild or no symptoms, and was later put into the El Tor biotype, to distinguish it from the form causing cholera (classical biotype). The El Tor biotype differed by being haemolytic, and positive in the Voges Proskaur reaction that detects a specific fermentation pathway. The 6^th^ pandemic continued until 1923, but from then on was largely confined to Asia (and thus not pandemic). The 7^th^ pandemic, which erupted in 1961, was El Tor in biotype, which was quite unexpected. However there had been several outbreaks of cholera, now known as pre-7^th^ pandemic outbreaks, in the Makassar area, Sulawesi, Indonesia between 1937 and 1957. The organism was El Tor but the disease resembled true cholera in severity and mortality. It differed in not spreading rapidly but remained localised as reported in a review soon after the events [2]. The 7^th^ pandemic (also El Tor) arose in the same area, and is generally thought to have arisen from the Makassar outbreak strain. It spread rapidly and eventually fully replaced the classical biotype 6^th^ pandemic strain, which was present in India throughout the period between the 6^th^ and 7^th^ pandemics and for many years after the 7^th^ pandemic arrived (see also Supporting Introduction in Text S1).

*V. cholerae* has also been found in water, particularly brackish water, and associated sediments, and also on invertebrates in these habitats. These environmental isolates are phenotypically very diverse with over 200 serotypes reported. They resemble the El Tor biotype, but the term is not used as the biotyping is confined to serotype 1. The fact that the 6^th^ and 7^th^ pandemics are of different biotype, with the 7^th^ pandemic strain more similar to environmental isolates than the 6^th^, suggests that the 7^th^ pandemic strain did not arise from the 6^th^ pandemic but arose independently.

**Figure 1:**
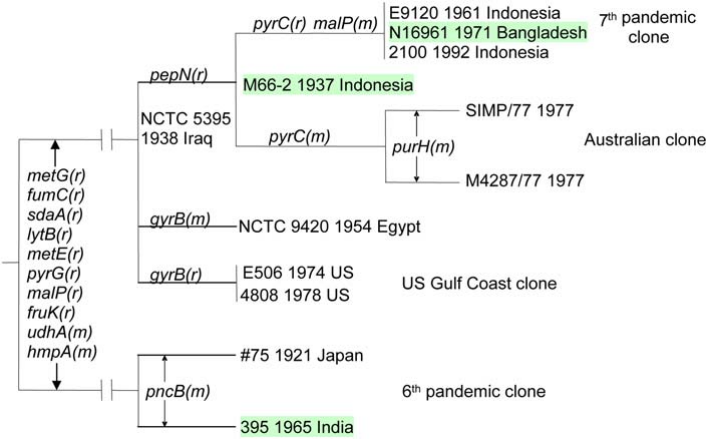
Relationships of M66-2, 6^th^ and 7^th^ pandemic clones, and other closely related toxigenic strains based on 26 house keeping genes (Salim *et al.* 2005). The relationships of the strains are identical to those in Figure 2 of Salim *et al.* (2005) but the tree is presented as phylogram as commonly used for easy interpretation. The mutational (m) and recombinational (r) changes with gene names are marked on the branches. The 3 strains compared in this study are highlighted in light green colour. The branch lengths are not drawn to scale.

We have already looked at the relationships of the pathogenic strains using sequence of 26 house keeping genes [3]. This confirmed earlier conclusions from several groups, based on MLEE and sequence data, that the 6^th^ and 7^th^ pandemic clones, the pre 7^th^ pandemic outbreak isolates, and some other pathogenic strains are related. However with 26 genes and 12 isolates an unambiguous tree was obtained, with no reverse or parallel events. It was also clear that the 6^th^ pandemic clone stands apart from the other pathogenic strains (Figure 1). Single base change mutational and multi bases change recombinational events could be distinguished, and this showed that there has been extensive recombination, with 8 of the 26 genes having undergone recombination in either 6^th^ or 7^th^ pandemic lineage since divergence. A 1937 Makassar outbreak isolate (strain M66-2) appeared to be on the direct line to the 7^th^ pandemic. The picture that we have for *V. cholerae* is of a species that is diverse with as many O antigen forms as *E. coli*, with one lineage that has adapted to a new niche as an intestinal pathogen of humans. On at least 2 occasions this pathogenic form developed a high level of transmission to become pandemic [3], [4].

These circumstances provide an ideal opportunity to study at DNA level the events that enabled the Makassar outbreak strain to become a pandemic strain and enabled the 7^th^ pandemic clone to fully replace the 6^th^ clone. We sequenced the genomes of strain M66-2, and 6^th^ pandemic isolate O395, and compared them with the published genome of 7^th^ pandemic isolate N16961. We describe a method to distinguish mutation from recombination-based changes over a full genome, and this provided the data to assess the rate of mutational change, as the 7^th^ pandemic strain N16961 was isolated in 1971, and the 6^th^ pandemic strain, O395, was isolated in 1965. Comparison of the genomes enables us to document the changes that had occurred during divergence of the three clones. Significant findings include an estimate for the mutation rate that is 100 times higher than generally assumed, a model for change in the major integron, and assessment of mutational, recombination, integron and other evolutionary events to the separate lineages.

## Results and Discussion

### General Genome Features and Genome Rearrangement

**Figure 2:**
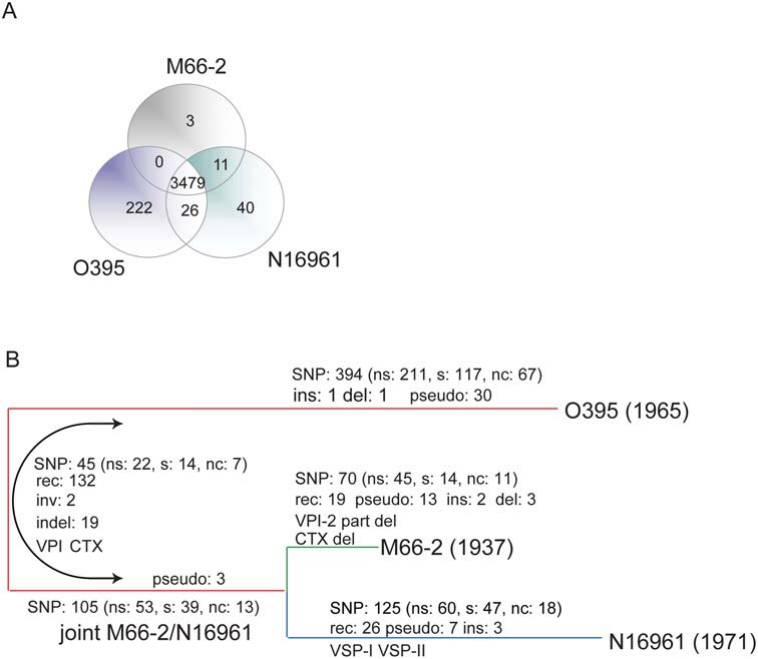
Distribution of genes among the 3 genomes and evolutionary events during the divergence of the 3 strains. (A) Venn diagram showing number of genes unique to each genome or present in 2 or 3 genomes. Genes in the major integron, pseudogenes and their homologs excluded. (B) Relationships of M66-2, N16961 and O395. Branch lengths are proportional to the estimated time frames for divergence, which were based on synonymous mutations as described in the text. All events were allocated to specific lineages if possible, or to the O/NM divergence as shown (details in Supporting Text S1). Note that numbers of SNPs have been adjusted for the proportion of the genome covered after allowing for recombination segments (see Supporting Methods Text S1 for details). ns, non-synonymous; s, synonymous; nc, non-coding region; rec, recombination event; inv, inversion; ins, large insertion; del, large deletion; indel, insertion or deletion in one of 2 lineages; pseudo, pseudogenes.

**Figure 3:**
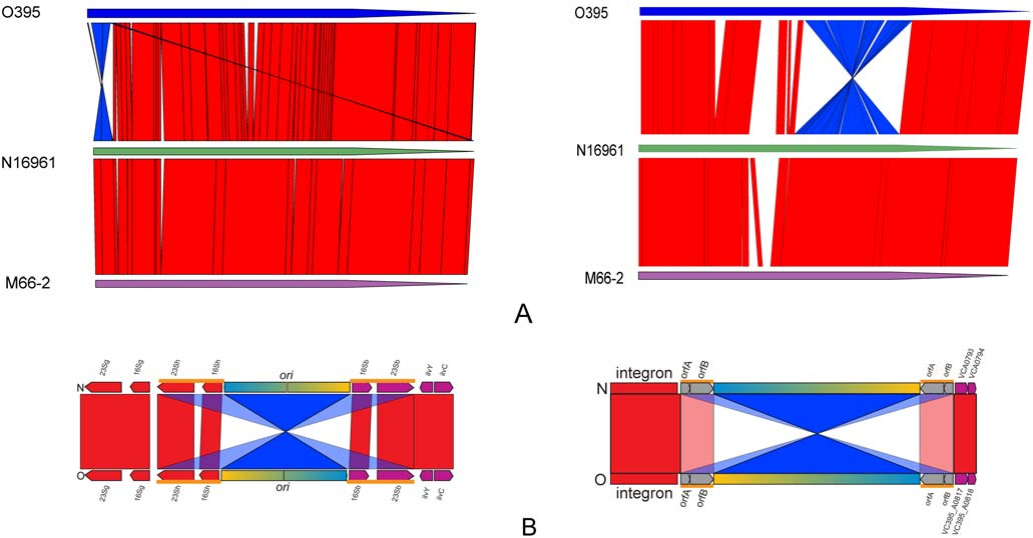
Alignment of the whole genomes and inverted regions. (A) Alignment of the O395, N16961 and M66-2 large (left) and small (right) chromosomes. (B) Alignment of the inverted regions of O395 relative to N16961 /M66-2 (not to scale). Both inversions are bracketed by inverted repeats comprising ISVch4 elements (small chromosomes) and *rrn* operons h and b (large chromosomes). The inverted repeats marked by orange bold line, align to both copies in the other strain, as shown in the figure by red bands and light blue bands connecting them to the proximal and distal homologues respectively. The inversions are assumed to have arisen by homologous recombination between these repeats, but sequence comparisons did not have any indication of recombination sites and so it is not possible to determine precisely where the break points should be.

The *V. cholerae* M66-2 and O395 genomes were sequenced and, as expected for *Vibrio* genomes, both comprise a large and small chromosome. The statistics for the three genomes are shown in Figure 2A and Table S1. The genomes are generally collinear, but in O395 each chromosome has an inversion relative to the others (Figure 3).

### Three Times as many Mutations as Recombination Events, but More Genes are Affected by and Many More Base Substitutions are due to Recombination

**Figure 4:**
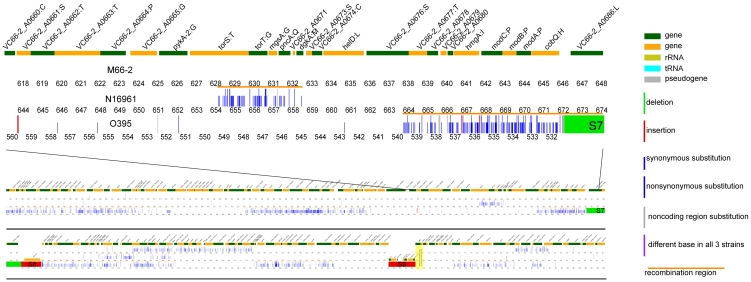
Alignment of the of M66-2, N16961 and O395 genomes. Top: View to show gene annotation. Below: Zoom-out view to include two 100 kb segments to show the alignment of 3 genomes. At top of each alignment is annotation of M66-2, and below 3 bands indicating how M66-2, N16961 and O395 differ from the other genomes. Map positions in kb given below each genome. Large indels shown as blocks of colour (green shows deletion, red shows insertion) named as in Table S5, and gene names or locus tags are also shown for the indels not in M66-2. SNPs of various classes indicated as shown in the key, and clusters of SNPs proposed to have entered the lineage by recombination indicated by orange bars as shown in the key. Full genome alignments are shown in Figure S1.

As predicted from our analysis of individual gene sequences, we could distinguish recombinant segments with a relatively higher frequency of base substitutions, and developed a formal procedure for identifying recombinant segments in the genome (for details see Supporting Methods in Text S1; Figure 4 and Figure S1). Base changes outside these segments were treated as mutations, and each mutation or recombination event was allocated to the M66-2 lineage, the N16961 lineage, or to the divergence between the joint M66-2/N16961 lineage and the O395 lineage (the O/NM divergence), as shown in Table 1 and Figure 2B. We also showed that recombinant segments were randomly distributed in the M66-2 and N16961 lineages, and those attributed to the O/MN divergence are probably also randomly distributed, but there is some distortion probably because with 32% of the genome affected, some recombinant segments overlap (see Supporting Methods in Text S1). It can be seen that there were far more recombination and mutation events during the O/NM divergence, than in either M66-2 or N16961 lineage, and also that M66-2 had undergone significant change. There is no genome sequence suitable for use as an outgroup to allocate events to the O or MN lineages, as examination of a series of genes shows that there has been extensive recombination, such that individual gene trees are not congruent and not useful for analysis of strain relationships. However for the 368 mutational changes we can ask if that particular base has undergone any change in the genome sequences that we have for comparison, which are in effect a complex array of lineages reassorted by recombination, as if not we can use that information in effect as an outgroup. We took 5 genomes that are outside of the pathogenic lineage under study, and for each of the mutational SNPs in the O/MN divergence, determined the base present in these strains. Of the sites the base present was same in all 5 genomes, and was either the base found in the O395 genome or that in both N16961 and M66-2. On this basis we allocated most of these changes to either the O or MN lineage. For some sites the sequence segment involved was not present in any of the outgroup strains, while for others it was not sufficiently consistent to enable us to determine the lineage. For details see Table S2. In this way we were able to root the tree as shown in Figure 2.

**Table 1:** Summary of mutational and recombination changes in the 3 *V. cholerae* genomes

	Mutational SNPs*					Recombination events							
Lineage	NS†	S†	NC†	Total†	No. genes‡	No. events§	% genome	Range of variation (%)	NS	S	NC	Total	No. Genes¶
M66-2	43 (45)	14 (14)	11 (11)	68 (70)	57 (43)	20 (11)	3.94	0.20–6.17	148	1509	283	1940	151 (23)
N16961	57 (60)	44 (47)	17 (18)	118 (125)	97 (55)	27 (13)	5.68	0.31–2.89	479	2010	405	2894	210 (28)
MN	36 (53)	27 (39)	9 (13)	72 (105)	61 (35)								
O	143 (211)	79 (117)	45 (67)	267(395)	208 (137)								
O/MN	15 (22)	9 (14)	5 (7)	29(43)	12 (17)	134 (77)	32.60	0.15–17.65	3195	14305	2359	19859	1230 (172)
Total	294 (391)	173 (231)	87 (116)	554 (738)	415 (273)	181 (101)			4022	17824	3047	24893	1443 (209)

* Excludes SNPs in regions thought to have undergone recombination, the *ctx* region, the integron and *rrn* operons. NS, non-synonymous, S, synonymous, NC: non coding.

† The number in brackets is number of SNPs extrapolated to the whole genome, taking into account of the proportion of the genome having undergone recombination. We excluded the *ctx*, integron and *rrn* operons in the calculation of total genome size for this purpose. These were used for calculating branch lengths for fig 1B and divergence times.

‡ The number in brackets is number of genes carrying at least 1 non-synonymous SNP.

§ The number in brackets is number of recombination regions that contain genes with ratio of synonymous to non-synonymous substitution rate less than 4.

¶ The number in brackets is number of genes with ratio of synonymous to non-synonymous substitution rate less than 4.

The level of change observed in M66-2 puts it on a side branch to the main lineage to N16961, and this was not predicted from the history, as M66-2 was isolated in 1937, in the first of a series of El Tor outbreaks from 1937–1959, in Makassar, the same region in which the 7^th^ pandemic arose in 1961, and it had appeared from that to be on the direct lineage to the 7^th^ pandemic clone (see Supporting Introduction in Text S1).

Recombination has introduced substantial changes to these genomes with 32.60% replacement during the O/NM divergence and 9.52% during divergence of N16961 and M66-2 (Table 1). The size distribution of recombinant segments is given in Figure S2. The largest recombinant segment is 70 kb with the majority between 1 and 20 kb. *V. cholerae* can undergo gene transfer by conjugation, transduction and transformation [5]but we have no means of inferring the processes involved. However the sizes are all within the range observed for transduction in *E. coli* [6]. Some must result from overlap between events but clearly recombination can introduce large segments of DNA. Sequence variation within recombinant segments varies from 0.15% to 17.65% suggesting that all or most donors are *V. cholerae*. In the 3 lineages that we can distinguish, there are in total 467 intragenic mutations involving 415 genes, and 181 recombination events involving 1446 genes. However, there are 24893 base substitutions in recombinant segments, more than 44 times the number (554) attributed to mutation (Table 1). However some of the substitutions in the recombinant segments will be due to mutation after the recombination event. The number of mutations that would have occurred if the rate had been the same in recombinant segments as elsewhere is 740 (Table 1). If we assume that half of the additional substitutions would have occurred before recombination and thus lost, we have 24800 of the observed substitutions due to recombination and 647 due to mutation, indicating that there have been 38 times as many of the substitutions observed due to recombination than to mutation.

### Pseudogenes Accumulated During Development of Pathogenicity

The distribution of pseudogenes is interesting (Figure 2B and Table S3). The common ancestor of the 3 strains must have had very few pseudogenes as there are only 6 common to the three genomes, but there are 53 cases of pseudogene formation during divergence. For pseudogenes we assume that in general the mutation in the lineage involved loss of function, and hence can attribute the pseudogene mutations that occurred during O/NM divergence to either the O or MN lineage. On this basis 30 occurred in the O395 lineage, 3 in the N16961/M66-2 common lineage, and 7 and 13 respectively in N16961 and M66-2 after their divergence (Table S3).

Most of those pseudogenes differ from the parent form only by a single frameshift mutation, or base change to give a stop codon. Also the 6 pseudogenes common to the 3 strains, are identical in all 3, except for VC0254 which has an additional indel in O395. Note that because even very low levels of sequencing error would affect these conclusions, the sequences for the regions involved were verified as discussed in Supporting Methods Text S1. Most pseudogenes are present in only one strain and *a priori* it is possible that the mutations involved occurred after strain isolation. We therefore looked at the sequence data for MO10, an O139 strain isolated in 1992, the first year in which that O139 variant of the 7^th^ pandemic clone was observed, as it is a descendent of the O1 form of the 7^th^ pandemic clone[7], although N16961 may not be on the direct lineage. The data suggested that at least 4, and perhaps 6, of the 7 pseudogenes attributed to the N16961 lineage, predated isolation of N16961 (2 genes were not in the current release of the MO10 sequence so no information, and one was a functional gene, so that N16961 pseudogene could have arisen before or after isolation). Details for the genes involved are given in Supporting Information Text S1.

### Gain and Loss of Cassettes in the Major Integron but Otherwise Conservation of Cassette Order

*V. cholerae* has a very large integron which fits the usual pattern of having at the 5′ end an *intI* gene followed by an *attI* site, with a series of cassettes downstream. In common with other *Vibrio* species it has many more cassettes than in the archetypal drug resistance integrons, and is often called a superintegron. Also the *attC* sites at the 3′ end of each cassette have a relatively conserved sequence [8]. Both M66-2 and O395 include a major integron that fits this pattern, and is in the same location as in N16961 [9]. The 3 *intI* genes are identical as are the *attI* sites, and there are 139, 142 and 179 cassettes respectively in the integrons of M66-2, O395 and N16961, with 168, 188 and 214 genes respectively. *V. cholerae attC* sites are generally species specific [10]. And using data from several genomes we were able to show that some in the *V. cholerae* genomes came from other *Vibrio* species, presumably associated with new cassettes (see Supporting Discussion Text S1 and Figure S4 for details).

**Figure 5:**
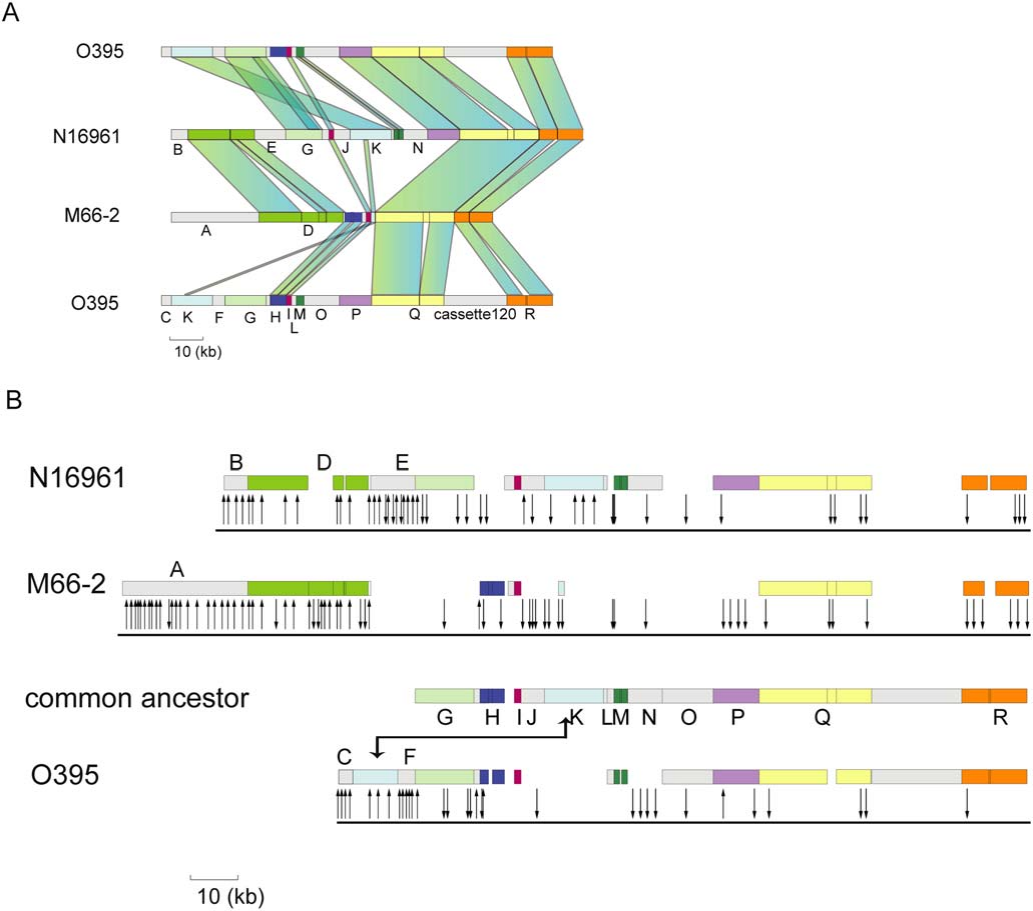
Homologies among cassettes of the integrons of M66-2, N16961 and O395. (A) Comparison showing major blocks of cassettes. (B) Alignment with gaps to properly align homologous cassettes for blocks F to R, thought to have been in the common ancestor as shown in row 3: gaps represent segments lost in that strain. Lines with arrows below represent the proposed copying of cassettes. Upward pointing arrows indicate cassette proposed to have been copied by IntI from a downstream cassette. Downward pointing arrows indicate proposed donor cassettes with some no longer present in that strain. Details are shown in Figure S4.

There is substantial conservation of cassette order with cassettes generally in blocks with a common cassettes order within the blocks, and block boundaries determined by differences in distribution of the blocks among the 3 strains. The distribution of these blocks, named A–R, is shown in overview in Figure 5, with details for blocks and cassettes given in Figures 5, S3, and S4. Figures 5 and S4 in particular can be usefully used in conjunction. Blocks D, G, H, I, K, M. P, Q and R contain mostly cassettes present in all 3 or the same 2 strains, and in the same order in each strain. These cassettes are presumed to have been in the relevant common ancestor, and are therefore treated as a single occurrence of that cassette, to give a total of 271 cassette locations as shown in Figure S4. The distribution of cassettes at each location is shown in Table S4.

Many cassettes are present at more than one location within a strain or at different locations in different strains, as has been noted before [10]. The sequences are generally identical and it seems that they arose by duplication rather than independent transfer from other strains. There were other cassettes that were near identical for which the same applies, as some divergence by mutation is expected, and for the purposes of this analysis we consider both identical and near identical (less than 1% sequence difference) cassettes to have arisen by duplication within the lineages under study. Treating these near identical cassettes as variants of the same form gives us a total of 207 cassette forms occupying the 271 locations. The relationships of the 207 cassette forms were assessed by using BLASTN with a cutoff e-value of 10^−10^, which puts 92 of them into 22 groups of 2 or more related cassette forms. The distribution of these cassettes is shown in Table S4. There were an additional 79 forms (Table S4, cassette forms 93–171) that were present in more than one strain and/or location but not allocated to groups because they are identical or near identical within each form. Finally there are 36 cassettes (Table S4, cassette forms 172–207) that are found in only one strain at one location.

### Pattern of Cassette Copying in the Major Integron

Integrons can gain new cassettes at the 5′ end by incorporation at *attI* [11]. M66-2, N16961 and O395 have blocks (A, B and C) of 40, 9 and 4 cassettes respectively at the 5′ end, that fit the pattern expected for cassettes added sequentially at *attI* since their divergence. M66-2 and N16961 share the downstream block D, also potentially added cassette by cassette at *attI* prior to strain divergence (Figure 5). A phylogenetic analysis of the *attC* sites indicates that many of these cassettes were of foreign origin (details in Supporting Discussion Text S1).

However as discussed above many cassettes are present at more than one location. There are 39 cassettes are present in duplicate, and 6, 3 and 1 with 3, 4 or 5 copies respectively (see Table S4), all most easily explained by copying of pre-existing cassettes in the same integron. The details of this proposal are presented in Figure 5 and Figure S4. For these cassettes, most of the upstream (new) copies are separated as a group from most of the downstream (donor) copies, although there is overlap of the 2 regions (Figure 5B). This pattern is assumed to reflect copying from downstream to upstream locations, as the only known mechanism for adding individual cassettes is at the upstream end of the integron. On this hypothesis 21 of the 40 cassettes in block A, proposed to have been added to M66-2 since divergence from N16961, were copied from downstream cassettes, one (M6) from another cassette in block A, 12 from cassettes still in M66-2, and 8 from cassettes in N16961 and presumably since lost in M66-2.

The original evidence was for excision of cassettes as circular DNA and integration at *attI*. It was shown that IntI can excise a cassette to give a circular form, and that this circular form can then be incorporated at the *attI* site, again by IntI, to give a new cassette downstream of *attI*, effectively transferring a cassette from one site to another. These experiments involved cassettes in different integrons, so gave no indication of the status of the donor integron after “excision”, but *a priori* did not provide a model for copying of a cassette while leaving the original intact. However under the current model for the action of IntI [12], [13], the formation of the translocation intermediate involves only a single strand of the donor cassette, and it is entirely plausible that at the time of its removal as an IntI-associated folded single strand, it would be replaced by repair DNA synthesis on the complementary strand. This would generate the pattern that we observe if this IntI-associated copy were later inserted into the same integron. Indeed our observations provide support for such a model for generation of the IntI-associated recombination intermediate. There are alternative explanations for the copying process, such as copying from one fork to another after DNA replication, or by involvement of the chitin induced DNA transformation between cells of the same clone forming a biofilm on chitin[14], [15]. The latter paper showed that DNA released by one cell could be taken up by another in the biofilm. The experiments involved homologous recombination but any circular cassettes present would also be released, and if taken up could be incorporated at *attI*. If both donor and recipient were from the same “colony” in the biofilm, there would be duplication as inferred from the distribution of cassettes.

### Twenty-nine Large Insertions and Deletions occurred during divergence of the 3 lineages

**Figure 6:**
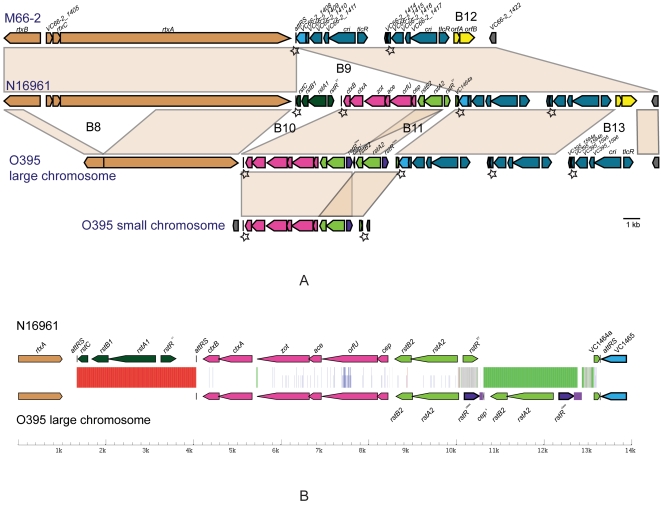
A. Comparison of the CTX phage regions in the 3 genomes M66-2, N16961 and O395. The 6 indels (B8–B13) in this region are also shown and discussed in Supporting Discussion Text S1. The CTX phage on the small chromosome in O395 is also shown for comparison. The genes are named for M66-2, and the genes of the CTX phage missing in M66-2 are named in N16961. The colour codes are; orange, *rtx* toxin region; black, the 17 bp attRS sites; red, core region of the CTX phage; dark green, the RS1 (*rstC, rstB, rstA, rstR*) region of the CTX phage of N16961; light green, the RS2 (*rstB*, *rstA*, *rstR*) region of the CTX phage of N16961; purple, the divergent *rstR* gene of RS2 of O365; light blue, genes not related to CTX; dark blue, the 5-gene toxin-linked cryptic (TLC) element–2–3 copies in each strain. The first and second copies of TLC differ between N16961 and O395 by 2 and 3 SNPs respectively. Only *cri* and *tlcR* are named and the others given locus tags as in N16961; yellow, IS3. B. Plot of base changes in the CTX phage genomes of O395 and N16961. The base changes in the homologous region of the CTX phage genome between O395 and N16961 were plotted using the same approach as in Figure 4. Except that red and green are specifically for presence and absence respectively in N16961 relative to O395. The differences in *rstR* are too numerous to be shown. The residual fragment of the *rtxA* gene upstream of the CTX phage in O395 differs by 1 base from the homologue in N16961.

There are 29 insertions and deletions involving at least one gene, which we refer to as large indels to distinguish them from mutations involving one or few base pairs. They are named B1 to B20, and S1 to S9 respectively in the large and small chromosomes (Table S5), and marked in Figure S1 or Figure 6. Three (B9, B16 and B20) can be identified as deletions and 2 (S2 and B17) as insertions in M66-2, as we have O395 as outgroup, and likewise for the 3 presumed insertions (B2, B3 and B5) in N16961. In addition B8 and S3 are thought to be a deletion and insertion respectively in O395 for reasons given in the Supporting Discussion Text S1. The remaining 19 indels are attributed to events during the O/NM divergence, with 15 present and 4 absent in O395.

The most significant additions are the 2 known islands (VSP-I and VSP-II) [16] in N16961 and 2 novel islands in O395 (B1 and B4). O395 also has a K139-like and 2 Mu-like phages. The CTXΦ is an indel because of its absence in M66-2, and an extra copy present on the small chromosome in O395, which is identical in sequence to that on the large chromosome. More interestingly, the CTXΦ genomes of O395 and N16961 contain different *rstR* genes at the ends, and have significant base differences throughout the phage genome except for *ctxA,* which is identical, and *ctxB* which differs by only 2 bases (Figure 6), suggesting replacement of the whole or part of the CTXΦ in one of the strains. There is further discussion of the indels in Supporting Discussion Text S1.

### Estimating Divergence Times for 6^th^ and 7^th^ Pandemic Strains and for 7^th^ Pandemic from the Makassar Outbreak Strain Required Recalibration of Molecular Clock

With full genome sequences for 3 of the isolates it is possible to estimate the divergence times for the 2 nodes present in Figure 2B. We used only mutational changes identified above to estimate the timeframe for the changes, and only synonymous substitutions as is traditional [17]–[19], referring to them as synonymous SNPs (Single-Nucleotide Polymorphisms) as at each site there are 2 alternative nucleotide bases. We excluded regions inferred to have undergone recombination, but extrapolated from the number of mutational synonymous SNPs observed to cover the whole genome (see Table 1 and Supporting Methods Text S1). The exclusion of recombinational SNPs has been used before for whole genomes, for example for *Y. pestis* [20], [21] but the problem of recombination did not arise in these studies (see also Supporting Methods Text S1). The widely used Dykhuizen and Whittam divergence rates [22], [23] gave 10,000 to 50,000 years for divergence of the two pandemic strains and 2,000 to 10,000 years for divergence of M66-2 and N16961 (Table S6). This takes us long before epidemiological records for pandemic cholera and is also not consistent with the Makassar outbreak being a precursor to the 7^th^ pandemic.

However it has recently been shown that species that diverged less than about a million years ago have accumulated up to 10 fold more than the expected number of SNPs, as many of the mutations seen in intraspecies comparisons are later lost due to random genetic drift or purifying selection [24], [25]. With an accurate record of the history of these strains, and in the absence of any remaining basis for an externally based rate of divergence, we took the split between M66-2 (isolated in 1937) and N16961 (isolated in 1971) as the basis for assessing relative time frames. There have been 14 synonymous mutations in M66-2 since its divergence from N16961 compared with 47 for N16961. To calculate the divergence rate, we assumed the same rate of change from divergence to isolation in 1937 and 1971 respectively, which gave a rate of 0.97 SNPs/genome/year and a divergence date for M66-2 and N16961 in 1923. Applying the same rate of change to their common MN lineage gives an estimated divergence date of 1880 for the 6^th^ and 7^th^ pandemic strains (see Methods for details). Note that as for pseudogenes, the sequences for the regions involved were verified as discussed in Supporting Methods Text S1. These dates are clearly estimates. There will be statistical sampling errors, and these will be significant as the number of synonymous SNPs between N16961 and M66-2 is low, and this was used to calibrate the mutation rate used to estimate both divergence dates, Also there is no reason to expect a constant rate of divergence over such short periods, as many factors will vary between the different lineages and there is not time for these to average out. For example the average generation time will depend markedly on the fraction of time spent in the host and environment, which will vary over time, Also the effective population size will vary and affect retention of the mutations involved. Indeed O395 and N16961 have clearly had different mutation rates as O395, isolated in 1965, has 30% more mutations than N16961 isolated in 1971. However, even as approximations, these estimates fit well with the fact that the El Tor outbreaks first appeared in 1937 and the 6^th^ pandemic started in 1899. They are much more realistic than the 10,000–50,000 years ago as estimated using conventional rates.

We conclude that for the *V. cholerae* pandemic strains the traditional criteria give divergence times about 100 fold too long. The rate estimates of Ho et al. [24], [25] were based on related species or forms of a species with good fossil data for divergence times, but our conclusion on much more limited data seems realistic given that 1 million years gave a 10 fold increase over the long term rate for accumulation of divergence, and we are dealing with much shorter periods. It seems clear that as for the cases analysed by Ho et al., we must use data appropriate to the timeframe involved rather than assume a constant rate for accumulation of SNPs. It is also probable that for clones within species, there will be variation in rates of divergence between species and even within species for clones in different niches. If we are to have realistic estimates of divergence times we will need to accumulate data for a range of species from different environments. We also caution that this first application of the approach is based on a very small sample and certainly not so precise as to preclude the possibility that pandemics 1 to 6 were due to the same clone as has also been suggested [1].

A major reason for studying the evolution of *V. cholerae* is to better understand its pathogenicity and pandemicity. We undertook several analyses focusing on this issue, and while the results were informative, there were no clear “suspects” for the change from causing local outbreaks as exhibited by the Makassar outbreak clone represented by M66-2, and pandemic capacity as exhibited by the 7^th^ pandemic clone, represented by N16961 (for details see Supporting Discussion Text S1, Figure S5, Figure S6 and Table S7).

### Concluding comments

The 3 genomes we compare represent critical stages in the history of cholera. The 6^th^ and 7^th^ pandemic strains were both major in their time, but 7^th^ has now fully replaced the 6^th^ [26] and is clearly fitter in evolutionary terms. The Makassar outbreak is seen as being at a critical stage in the development of the 7^th^ pandemic strain. The three strains are closely related such that recombination events were generally well separated in the genome, enabling us to distinguish between mutation and recombination events. The genomes have been very dynamic with substantial changes through mutation, recombination, acquisition of genes in islands, and acquisition of cassettes in the major integron. We now have the catalogue of genetic differences between the 3 strains that must be responsible for differences in pathogenicity and pandemicity, opening up new areas for research.

We have been able to put a realistic timeframe to the divergence of the pandemic strains because 1) we could distinguish between mutation and recombination events and used only the mutational SNPs to calculate divergence rate, and 2) N16961 (7^th^ pandemic) is thought to be derived from a Makassar outbreak of known date and represented by M66-2, which allowed us to infer a divergence rate, that could then be used to infer a date for divergence of the 6^th^ and 7^th^ pandemic strains. *V. cholerae* is unusual in having a defined date and location for the origin of a major pandemic that allowed this approach**.** There are other landmarks in the spread of *V. cholerae* which will allow extension of the approach to further refine our understating of the evolution of pandemic cholera, and suitable circumstances are found in some other species. There are certainly other estimates of divergence times for important human pathogens that are surprising and worth reanalysis, such as *E. coli* O157:H7 (∼40,000 years) [27], *S. enterica* serovar Typhi (10,000–43,000 years) [28], and our own estimate for *Shigella* (35,000–270,000) [18].

## Methods

For most methods and further details of those discussed below, see Supporting Methods Text S1.

### DNA sequencing and annotation

*V. cholerae* strains M66-2 and O395 were sequenced by random shotgun sequencing. For M66-2 the final genome is based on 62,004 reads from an ABI 3730 sequencer. For O395 the final genome is based on 14,210 reads from an ABI 3730 sequencer and 334,497 reads from a Roche 454 GS-FLX System. Open reading frames from 30 amino acids in length were predicted using Glimmer 3.0[29] and verified manually using the annotation of N16961. Artemis [30] was used to collate data and facilitate annotation. Pseudogenes were detected by comparing the genome sequences of M66-2, N16961 and O395 using the program Psi_Phi [31] and verified manually (for details see Supporting Methods Text S1).

The complete nucleotide sequences and annotations of the *V. cholerae* strain M66-2 and O395 have been deposited at GenBank under the accession number CP001233, CP001234, CP001235 and CP001236 respectively.

### Alignment of the genomes and verification of sequence variations

The genomes of N16961 and MO10 were downloaded from GenBank (RefSeq accession numbers NC_002505, NC_002506 and AAKF00000000). Alignment of the genomes was done using Multi-LAGAN [32] and checked visually. Because of the significance that each variation carries in such closely related strains, we verified the sequence accuracy for all mutational SNPs and all pseudogenes (for details see Supporting Methods Text S1 and Table S8).

### Differentiation of random mutations and recombination

(for details see Supporting Methods Text S1 and Figure S7)

### Calculation of divergence rates and dating the clones

N16961 was isolated in 1971, and is thought to have diverged from M66-2 in about 1937, when M66-2 was isolated in the first of the El Tor outbreaks in Makassar. There are 14 synonymous SNPs since the MRCA with N16961 compared with 47 for N16961 (Table 1). We assumed that the rate of change per year (r) was the same in both lineages, ie (1937-X) * r = 14 and (1971-X) * r = 47**,** where X is the divergence date and r is the number of mutations accumulated per annum since divergence. This is equivalent to r = (47−14)/(1971−1937) or 0.97 per year and X is 1923. There are 39 synonymous SNPs which can be allocated to the MN common lineage. We applied the same rate of mutation to the MN common lineage, but first notionally allocated the 14 synonymous SNPS in the O/NM divergence that could not be allocated to the O or NM lineage, in the proportion of those that could be allocated to the O and MN lineages, to give a final allocation of 42 to the MN lineage and a divergence date of 1880 for the MRCA of N16961 and O395. The number of synonymous SNPS allocated to the O395 lineage on this basis is 129 for a mutation rate of 1.5 of synonymous SNPs per year.

## Supporting Information

Text S1 This Supporting Text file includes Supporting Introduction, Supporting Discussion, Supporting Method and Supporting reference.
(0.19 MB PDF)

Figure S1 Plot of mutations, recombination events and indels in the genomes of strains M66-2, N16961 and O395
(6.69 MB PDF)

Figure S2 Histogram of the lengths of the recombinant segments
(0.29 MB PDF)

Figure S3 Comparison of the 3 integrons using artemis comparson tool and dot plots
(0.58 MB PDF)

Figure S4 Relationships of cassettes in the integrons of strains M66-2, N16961 and O395
(0.31 MB PDF)

Figure S5 Function Categories (COGs) of genes with or without mutational changes
(0.05 MB PDF)

Figure S6 Correlation of gene expression level with ratio of synonymous and non-synonymous substitution rates
(0.46 MB PDF)

Figure S7 Distribution of Inter-SNP segment (ISS) lengths for the 6 chromosomes
(0.37 MB PDF)

Table S1 General features of the M66-2, N16961 and O395 genomes
(0.02 MB PDF)

Table S2 Allocation of SNPs in the O/MN divergence to either O395 or MN lineage
(0.17 MB PDF)

Table S3 Pseudogenes allocated to the M66-2, , N16961, MN and O395 lineages
(0.04 MB PDF)

Table S4 Integron cassette groups
(0.08 MB PDF)

Table S5 Major indels in the 3 genomes
(0.04 MB PDF)

Table S6 Substitution rates and divergence times
(0.04 MB PDF)

Table S7 Genes in recombinant segments with protein product having ks/ka less than 4
(0.10 MB PDF)

Table S8 Corrections to N16961 genome bases
(0.06 MB PDF)
