# Supplementary material for: A Recalibrated Molecular Clock and Independent Origins for the Cholera Pandemic Clones
Source: PLoS One. 2008 Dec 30;3(12):e4053. doi: 10.1371/journal.pone.0004053 (PMC2605724; doi:10.1371/journal.pone.0004053)
Supplement: Text S1 — This Supporting Text file includes Supporting Introduction, Supporting Discussion, Supporting Method and Supporting reference. (0.19 MB PDF) [file pone.0004053.s001.pdf]

A recalibrated molecular clock and independent origins for the cholera pandemic clones

Lu Feng[1,2,6], Peter R. Reeves[3,6], Ruiting Lan[4,6], Yi Ren[1,6], Chunxu Gao[1], Zhemin Zhou[1], Yan Ren[1], Jiansong Cheng[1], Wei Wang[1,5], Jianmei Wang[1], Wubin Qian[1], Dan Li[1] and Lei Wang[1,2,5,7]

[1]TEDA School of Biological Sciences and Biotechnology Nankai University and [2] Tianjin Research Center for Functional Genomics and Biochip, 23 Hongda Street, Tianjin Economic-Technological Development Area (TEDA), Tianjin 300457, China. [3]School of Molecular and Microbial Biosciences, University of Sydney, Sydney, New South Wales, Australia. [4]School of Biotechnology and Biomolecular Sciences, University of New South Wales, Sydney, New South Wales, Australia. [5]Tianjin Key Laboratory of Microbial Functional Genomics, Nankai University, 23 Hongda Street, Tianjin Economic-Technological Development Area (TEDA), Tianjin 300457.

[6] These authors contributed equally to this work.
[7]Correspondence should be addressed to LW. Email: wanglei@nankai.edu.cn

Supplemental Material

Table of contents

Supporting Text 2
Supporting Introduction 2
Supporting Discussion - Large insertions and deletions 4
Supporting Discussion – Analysis of *attC* sites in the integrons.. 7
Supporting Discussion – Pathogenicity and pandemicity 8
Supporting Methods 10
Supporting References.. 17

Supporting Text

Supporting Introduction

This paper sheds light on the relationships of 6th and 7th pandemic strains and an isolate from the first of 4 outbreaks in Makassar between 1937 and 1957. The Makassar outbreak strain is thought to be a precursor of the 7th pandemic. Now that we have data to assess the time frame, it is important to review the basis for considering the Makassar outbreaks to be a precursor to the 7th pandemic strain, and the following is a guide to the literature on which this perception is based. Three reviews, Linton [1], Pollitzer [2] and Felsenfeld [3], are particularly useful.

Key to the recognition of a relationship between the Makassar outbreaks and 7th pandemics is the division of O1 *Vibrio cholerae* into 2 biotypes. The history of biotyping in *V. cholerae* goes back to 1905 when 6 "strains" were isolated at the El Tor quarantine camp in Egypt from pilgrims [1]. The pilgrims did not have clinical cholera but the "organisms appeared identical morphologically, biochemically and serologically with the cholera vibrio." They were distinguished by being haemolytic and in this were similar to other non-cholera vibrios that had been studied earlier [4]. Haemolytic O1 vibrios were then isolated "repeatedly" at El Tor and in India but only rarely associated with clinical cholera [3,4]. For example Tanamal [5] summarising literature from that era says "El Tor vibrios were found repeatedly in mild diarrhoeas and symptomless persons. They were also seen in Bengal and Madras where they were isolated from wells and from carriers without clinical disease". The haemolytic strains were named El Tor and this is now recognised as a biotype, with the then known pandemic isolates being classical biotype. The 2 forms have sometimes been given species status but this is clearly not the case. All were of the same serotype, which we now attribute to having the type 1 O antigen. *V. cholerae* has many O antigens most of which are only found in environmental strains. Environmental strains are generally haemolytic, but the distinction into biotypes applies only to isolates that are O1 and toxigenic. There were occasional cases of disease caused by El Tor organisms and Pollitzer [2] cites outbreaks in Bulgaria in 1913, Kiel in 1916 and Poland in 1919.

The situation changed when outbreaks of cholera occurred in the island Sulawesi (known at the time as Celebes), Indonesia in 1937/38, 1939/40, 1944 and 1957. The disease itself was not distinguished clinically from true cholera [6] but the organisms were El Tor. The first outbreak involved 48 cases (registered), 26 from September to November 1937 (dry season) and 22 from January to April 1938 (wet season) all in a coastal area about 100 km long around Makassar in SW Sulawesi.

The second outbreak was from October 1939 to April 1940 around the Tempe Lake (37 patients). Also in December 1939 there were 15 cases in localities about 40 km north east from the lake. In June and July 1939 (sic) there were 10 cases about 120 km south of the lake area. The two outbreaks had an average mortality of 70% and a carrier rate of 4 per 1000. El Tor vibrios were found in 43% of wells. Vibrios were seldom found in water after the disease passed in that locality, indicating that the outbreak strain was human associated and not an environmental strain sometimes causing disease.

The third outbreak was in 1944 during the Japanese occupation. The Japanese manager of the grand hotel in Makassar became ill in mid February and died within 12 hours – El Tor vibrios were isolated from stools and vomitus. About 3 weeks later there was a case in the Takular prison and 6 carriers were also found. In June, 5 cases and 6 carriers in Watapone were found, all with El Tor vibrios.

The fourth outbreak was in 1957 when there were 70 cases in Makassar, mostly between January and April followed by single cases until February 1958. There were also 81 persons with paracholera (see below) in adjacent areas – a total of 151 cases in Sulawesi in a year. Of the 70 in Makassar 31 were taken to hospital and 13 died. 39 died in the villages without treatment. Morbidity was 2.2% and mortality of patients high at 90%. Vibrios were only found in stools during the outbreaks.

We have given this detail to show the nature of the change in the El Tor organism. The El Tor outbreaks showed that the Makassar strain differed from the earlier isolates from the El Tor quarantine station and elsewhere in causing cholera, but while the symptoms resembled those of "true cholera", the disease differed from true cholera in not spreading rapidly and appeared to give only occasional localised outbreaks. The disease was called paracholera or El Tor diarrhoea. In summary from 1937 to 1957 there were four El Tor outbreaks in Sulawesi over a 20 year period, but these died back each time within about a year, often with several years between outbreaks. This contrasted with annual outbreaks of "true cholera" in India. Also in contrast to the ready spread of "true cholera" the disease did not spread beyond the Makassar area apart from 2 cases in 1957 in Jakarta [7]. However, paracholera was not reportable at that time and may have occurred elsewhere and not been reported. Also a mild diarrheic outbreak caused by El Tor organisms was observed in the city of Ubol, Thailand, in 1960 in the wake of an epidemic caused by true cholera vibrios, but the origin of this outbreak could not be traced [3]. The strain causing the Makassar outbreaks described above was pathogenic but not pandemic.

The situation then changed again. In 1960 there were outbreaks in North Sulawesi and Jakarta. In 1961 there were 394 cases in central Java (117 deaths) in May –July and 95 (21 deaths) in August/September, 200 in South Borneo and 102 cases in Makassar. In the second half of 1961 there was cholera in Hong Kong, Macao, Sarawak and a major

outbreak in the Philippines (10,000 cases and 13% mortality) [8]. Sulawesi was no longer the main focus.

However although El Tor organisms spread rapidly, the number of cases due to classical cholera in its major base in what was then India meant that the number of classical cases globally was still much higher than those due to El Tor organisms [9]: between 1959 and 1963 the number of cases of Classical cholera ranged from 28,182 to 55,069 whereas those of El Tor cholera between 1961 and 1963 ranged from 10,088 to 13,393. However these El Tor outbreaks grew into the 7th pandemic which displaced the classical biotype as the pandemic form.

Supporting Discussion - Large insertions and deletions

The large indels discussed in the main text are divided into 6 categories for easy discussion below of their properties and the major factors that may contribute to their gain or loss.

1) IS and IS associated indels. 8 indels are IS elements and 3 are associated with IS elements. The IS elements are named as in ISFinder (http://www-is.biotoul.fr/is.html). Of the IS elements, 1 (B12) is in the CTX phage region of M66-2 and N16961, 4 (B14, B18, B19 and S4) in O395, 2 (B17 and S2) in M66-2 and 1 (B3) in N16961. The number of ISs in the *V. cholerae* genome is small with 27 IS insertions and 7 types of IS in the 3 genomes. But some ISs are damaged, and it appears that there are only 4 complete active ISs (IS1004, ISVch1, ISVch4 and ISVch5) that have undergone transposition. B3, B12 and S2 are ISVch4 transpositions. ISVch4 seems to be quite active as it is also present in 3 other large indels (B1, S9 and VPI-2), and the integron. There are a number of ISs in other larger indels for example in B1 and B5 but these will not be discussed here. S4 is at the end of a recombinant segment and perhaps came in during recombination with another strain, as proposed to have occurred in the entry of an H-rpt element in the *Salmonella* D2 O-antigen gene cluster [10]. B14 and B17-B19 are IS1004. There are 5 other copies of IS1004 in the genomes with 1 degraded and 1 in the integron. These 4 new events could be transpositions from any of the other four. B14 is identical to the copy in the integron while B17 and B18 are identical to VC0870. S4 is ISVch1, the only other copy of which is on the VPI [11] and it is possible that S4 transposed from the VPI. S6 in O395 contains 3 hypothetical genes and an ISVch5 insertion, of which there are 2 other copies including one in the integron. However, S5 and S6 are alternative insertions at the same site, which is within a region that has undergone recombination. The probable explanation is that the gain of one and loss of the other in either O395 or the M66-2/N16961 lineage resulted from a recombination event rather than independent insertion and deletion events. B16 is part of VPI-2 and next to an ISVch4 element, that presumably mediated the deletion as this is a well documented property of IS elements

[12]. S9 includes an ISVch4 element at its left end and is immediately downstream of another ISVch4 which is at one end of the large inversion. The two ISVch4 elements are identical and S9 insertion or deletion was probably mediated in some way by ISVch4.

2) Phage genomes. There are 3 phage genomes in addition to that of CTXΦ. Two are Mu-like phages (B6, B7) and the other is a K139-like insert (S1), all in O395. K139 phage [13] was first isolated from O139 outbreak strain MO10 and can be recovered frequently from strains of O1 El Tor isolates [13]. A related phage was found in O395 by Southern blot and PCR [13], and now confirmed by the genome sequence. The 2 O395 K139-like sequences differ by 8 SNPs, likely to be sequencing errors in the previously reported sequences. However the K139-like phage in O395 differs considerably from the MO10 K139 phage. For example, the integrase gene is more closely related to a homologue in *Vibrio vulnificus* than to the original K139 gene (data not shown). The K139 phage in MO10 is located on the large chromosome while the K139-like phage in O395 is located on the small chromosome. Interestingly the 2 genes flanking the K139-like phage in O395 contain a 252 bp direct repeat differing by only 10 SNPs, which may have been involved in phage integration.

The two Mu-like phages (B6 and B7) are 100% identical and are situated about 43 kb apart on the large chromosome. Both sites of insertion have base duplications (GGAGT and GTTACCA in B6 and B7 respectively) to give a direct repeat. It is known that Mu generates a 5-bp duplication [14]. Since Mu propagates by transposition it is possible that the original Mu phage that entered the cell had 2 rounds of replicative transposition to give 2 identical insertions. Classical and El Tor strains are best distinguished by different patterns of sensitivity to phages**,** and this may well be due in part at least to immunity conferred by the Mu and K139-like prophages.

3) CTXΦ related genes. M66-2 differs from N16961 by absence of the CTXΦ prophage CORE and RS regions (B9, Table S5 and Figure 6). It appears to be a simple loss and not a gain in N16961, because the US Gulf strain that branched off the 7th pandemic lineage before M66-2 [15] is also toxigenic [16]. It would also account for the disease being not distinguished from "true cholera" [6].

The CTXΦ of O395 on the large chromosome differs from that of N16961 in both structure and sequence variation. The structural differences lie in the arrangement of the RS elements. The O395 CTXΦ has the CTX CORE followed by the RS2 element, a partial *cep* gene and another RS2 element (treated here as an indel (B11)) while the N16961 CTXΦ has the CTX CORE flanked by an RS1 upstream (named as indel B10) and an RS2 downstream. Immediately downstream of the CTXΦ, there are 2 TLC elements in M66-2 and N16961 and 3 in O395. Many of the CTXΦ prophage, RS elements and TLC elements are separated by *attRS* sites, which are the integration site for

the CTXΦ. The first RS2 element in O395 is truncated at the 3' end as it lacks the VC1464a gene, and Davis et al. [17] suggest that the O395 CTXΦ is a fusion of 2 truncated CTX phages, the 1st having about 500 bp truncated at the 3' end after *rstR* including the VC1464a gene and the *attRS* site while most of the 2nd phage is deleted with only a fragment of *cep* and the RS2 element present. Tandem arrays of CTXΦ are well documented [18] and a deletion as proposed in such an array would account for the O395 situation and in particular the location of the remnant *cep* gene. This event would have occurred after the O/NM divergence. This could account for the additional RS2 element in O395. N16961 has only one CTXΦ and we have no basis for saying if the presence of one in N16961 or 2 in O395 is primitive, and B11 is allocated to the O/NM divergence. The various TLC elements have very few SNP differences and the different number in O395 and N16961/M66-2 could be due to gain or loss by recombination between adjacent copies after replication as proposed by Mekalanos [19], for reduction by homologous recombination to give excision at any time. The O395 and N16961 *rtx* region and TLC elements are very similar, but the CORE CTXΦ and RS 2 elements differ substantially (Figure 6), except for the *ctxA* genes that are identical and the *ctxB* genes that differ by 2 non-synonymous substitutions, with a segment of the *orfU* gene and rstR being extremely divergent. The many differences in the CTXΦ while the *ctx* genes are so similar suggest that the genes other than *ctxA* and *ctxB* were substituted in one lineage by recombination, but the alternative is that the phage were gained independently but had very little difference in the *ctx* genes. The substitution in one or other may have been driven by the major differences in *rstR* or *orfU*.

O395 has an additional CTXΦ phage on the small chromosome (S3) in addition to that present on the large chromosome [17]. It appears to be a copy made before the deletion that joined 2 CTXΦ as discussed above, as the two are identical in sequence, where aligned. But the small chromosome has a single intact CTXΦ.

4) Integrase containing islands. Four indels (B1, B2, B4, B5) contain an integrase gene of which 2 also have the expected direct repeats. B1 and B4 are inserts in O395 and have a recombinase/integrase gene as last gene. B1 is low GC with no direct repeats. B4 has a 16 bp direct repeat and part of its DNA is low GC. B5 is VSP-II, an insert in N16961, discovered using microarray hybridisation by Dziejman et al. [20] and mapped by O'Shea et al. [21]. The genome sequences define the island precisely. It is flanked by imperfect direct repeats and has an integrase gene as last gene and a tRNA gene downstream, indicating that the recombinase/integrase is using the tRNA sequence as insertion site as is common for integrases. B2 is VSP-I, an insertion in N16961 also found by Dziejman et al. [20] and like VSP-II, is present in 7th pandemic only. It is low GC but has no direct repeats. Although it has an integrase/recombinase gene at the 3' end, B2 may not have entered N16961 by integration, as it is located in a region with recombinant segments at both ends and may have hitchhiked in through homologous recombination in flanking

DNA after integration in the donor. However, the region is recombinant in all 3 strains and it is not clear what happened.

5) *rrn* locus. B20 is an rRNA operon. One of the *rrn* loci has two 16S-23S gene sets (g and h) in N16961 and O395, but only one (h) in M66-2. The other could have been lost by homologous recombination. The 7th pandemic strain is known to contain variable copies of the *rrn* operon [22] and the loss in M66-2 is not surprising.

6) Other indel events. Five indels (B8, B15, S5, S7 and S8) have no repeats or other indications of their origins. B15 contains a single gene coding for a hypothetical protein and is located within a recombinant segment, so is likely to have come in by recombination. S5, present in both N16961 and M66-2, is an alternative insertion to S6 at the same site as discussed above. S8 in O395 carries an ATPase gene, and is an alternative insertion to a block of 3 genes (S7) encoding hypothetical proteins present in the other 2 genomes. The exchange is likely to have occurred by recombination as the site is within a recombinant segment. B8 is a known deletion in the RTX gene region in O395 and is also present in 2 other Classical strains examined [23] (also shown in Fig. 4), rendering the RTX toxin non-functional. The deletion includes parts of *rtxA* and *rtxB*, the whole of *rtxC* and a newly annotated ORF in the middle.

B13 is an extra copy of the toxin-linked cryptic (TLC) element present in O395 downstream of the CTX phage on the large chromosome (Fig. 4). The TLC element was shown to be a cryptic plasmid by Rubin et al. [24] and is now known to be present in 2 tandem copies in M66-2 and N16961 but 3 tandem copies in O395. The first and second copies of the TLC differ between N16961 and O395 by 2 and 3 SNPs respectively suggesting that they were present as tandem copies before the divergence of the 2 clones.

In summary of the 29 indels, 19 appear as typical insertions that either inserted or deleted during the divergence of the strains, 7 (B2, B15, S4, S5, S6, S7, S8) are in recombination segments and were probably gained or lost as part of a recombination event, 2 (B13 and B20) were due to a change in number of a repeated element, and 1 (B8) was a partial gene deletion. In 2 of the 7 recombination cases there are alternate insertions at the same site (S5/S6 and S7/S8) so recombination involved gain of one and loss of another component. Thus 5 recombination events were involved in transfer of the 7 indels. In many cases it is not known if there was a loss in one lineage or gain in another as indicated in Figure 1.

Supporting Discussion – Analysis of *attC* sites in the integrons

It has been shown that most *attC* sites of *Vibrio* species, in *V. cholerae* also known as VCRs (*V. cholerae* repeat) [25], are species-specific and highly conserved within each

species [26,27]. We looked at the *attC* sites in the 3 genomes under study for indications of the origins of the cassettes. We extracted the *attC* sites from the genome sequences of 4 *Vibrio* species (*V. fischeri* ES114, *V. parahaemolyticus*, *V. vulnificus* CMCP6, *V. vulnificus* YJ016)[28-30], and *Vibrio sp*. DAT722 [27]), and aligned them together with those from the 3 *V. cholerae* strains studied here. Most *V. cholerae attC* sites fall into two major clusters. Cluster I *attC* sites (including 385 *attCs*) are very closely related and exclusively *V. cholerae* while cluster II *attC* sites (including 36 *attCs*) are mostly *V. cholerae*, but also include a few *V. metschnikovii* sequences, suggesting import by that species. The remaining *V. cholerae attC* sites (including 39 *attC*s) are quite diverse and were arbitrarily placed into 14 groups, with *attCs* from other species, indicating that they are within cassettes that have been imported from other species into *V. cholerae*. Their distribution is shown in Figure S4.

Cassettes conserved among the 3 strains generally have cluster I or II *attC* sites. However there are exceptions. For example M66-2 cassettes M99 and M104, present in all 3 strains, have *attC* sites from the 14 non- *V. cholerae* groups, suggesting that the cassettes were obtained from an outside source before the 3 strains diverged. Five of the 9 N16961 cassettes present at the beginning of the integron (block A cassettes) have *attC* sites from the 14 groups, suggesting that they are from another species. However, not all unique block A cassettes are associated with a foreign *attC*, indicating that some block A cassettes have been obtained from within *V. cholerae*. In contrast only one of the 40 cassettes at the 5' end of M66-2 (block A) have *attC* sites from the 14 groups, and most, including those unique cassettes, have cluster I *attC* sites. Many of these cassettes are also present in N16961 or O395 or duplicates within M66-2 (Figure S4). It seems that M66-2 got most of the block A cassettes from within *V. cholerae.*

Supporting Discussion – Pathogenicity and pandemicity

A major reason for studying the evolution of *V. cholerae* is to better understand its pathogenicity and pandemicity. This study gives us the catalogue of genetic differences between the 3 genomes that must be responsible for differences in pathogenicity and pandemicity. The gain of 2 major segments of DNA, VSP-I and VSP-II [20], is the most obviously relevant addition to the genetic information in N16961 although their functions are still to be determined. However any of the genes affected by mutation, recombination, gain/loss of DNA segments ranging from single genes to big islands, or gain/loss of cassettes in the integron, may have contributed to the rise of the 2 pandemic clones and the later replacement of the 6th.

We first looked at the 273 genes with 1 or more non-synonymous mutational SNPs using Clusters of Orthologous Groups (COG) [31].Only the functional category, "signal transduction", was significantly over represented (Z test, p=0.0023; Figure S5), and none of the recombinant genes were for over representation of any functional categories

recombinant genes were significantly over represented, although the proportion of signal transduction genes involved in recombination was higher than the average. The signal transduction genes that had undergone mutational or recombinational changes, included 28 (54.9%) of the 51 genes encoding proteins with GGDEF and/or EAL domains [32,33]. The proportion is significantly higher (Z test, p = 0.0038) than the overall genome wide changes at 35.4%, involving 1252 of the 3536 genes present in all three genomes.

Proteins with GGDEF and/or EAL domains modulate the cellular levels of c-di-GMP, which has an important role in controlling rugose colony formation, exopolysaccharide production, motility and biofilm formation [34]. Two of these genes, VC2454 (*vpvC*) [35] and VCA0074 (*cdgA*) [36], have been shown to affect the rugose phenotype, and both underwent recombination in the O/MN lineage, making them candidates for involvement in development of pathogenicity and or pandemicity.

V. cholerae has multiple iron transport systems with 12 loci and a total of 46 genes [37], of which 20 (43.5%) differ in the 2 pandemic clones in comparison to 35.4% for the genome overall, but the difference is not statistically significant

A proportion of the recombinant events are likely to have resulted from selection for specific beneficial alleles, and the genes involved are likely to have been under positive selection pressure in the donor, which will be reflected in the ratio of synonymous to non-synonymous substitution rates (K_s/K_a ratio). Traditionally a K_s/K_a ratio of less than 1.0 is considered to indicate positive selection pressure. However we consider a cut-off of 1.0 to be too conservative, as purifying selection acts in the opposite direction to positive selection pressure [38]. The average K_s/K_a ratio for the 26 house keeping genes previously studied [15] is 37.2 reflecting strong purifying selection against most non-synonymous mutations. 209 of the 1442 genes in recombinant segments have a K_s/K_a ratio of less than 4.0(Table S7), which still requires a 10-fold or greater proportion of non-synonymous substitutions than in house keeping genes. We further found that a higher proportion of these genes are expressed at high levels *in vivo,* based on data reported by Bina et al. [39]. Twenty (10.0%) of the 207 genes have an expression level of 1.5 or greater, in contrast to only 67 (5.49%) of the 1221 genes with a K_s/K_a ratio equal to or greater than 4.0 (Fisher's exact test, P = 0.027; Figure S6). These observations suggest some of the genes that could be targeted in functional studies. Note that not all of the 1442 genes were included as 14 genes had no expression data. These genes were either pseudogenes (8) or newly added genes (6) which were not present in the original annotation of the N16961 genome.

Supporting Methods

DNA preparation and sequencing

Chromosomal DNA from *V. cholerae* M66-2 was isolated by standard protocols. Two pUC118 libraries (inserts of 2–3 and 6–8 kb) were generated by mechanical shearing of chromosomal DNA. Double-ended plasmid sequencing reactions were done using an ABI BigDye Terminator V3.1 Cycle Sequencing Kit and an ABI 3730 Automated DNA Analyzer (Applied Biosystems). 55,726 reads were generated providing an 11.4-fold coverage, and assembled into 279 contigs using the PHRED PHRAP and CONSED [40] programs. Linkages among contigs were based on sequences of gap-spanning clones and comparison of the contigs to the published genome sequence of N16961. Sequence gaps were closed by primer walking on linking clones or sequencing PCR products amplified from genome DNA. All repeated DNA regions and low-quality regions were verified by PCR and sequencing of the product. The ribosomal RNA operon sequences were assembled separately by construction of DNaseI shotgun banks. The final genome is based on 62, 004 reads.

Chromosomal DNA from *V. cholerae* O395 was isolated by standard protocols. pUC118 libraries were generated as for M66-2 and 14,210 reads were generated using an ABI 3730 Automated DNA Analyzer. In addition O395 sequence data was generated with a Roche 454 GS-FLX System (through Creative Genomics, NJ, USA) using high-density pyrosequencing methodology [41]. 334,497 sequence reads were generated with an average length of 216 bases for approximately 72 Mb of data. The ABI 3730 and 454 sequencing data, were assembled by using the Newbler Assembler software provided by 454 Roche. The analysis yielded 147 contigs. Most of the assembly gaps were caused by repeat regions and the contigs were ordered based on the reference genome sequence of N16961 by Nucmer[42]. All gaps were closed by PCR and sequencing.

Verification of M66-2 and O395 SNPs

The conclusions from analysis of the sequence described in the main text and elsewhere in the supporting text include some which are dependent on an extremely high level of sequence accuracy. We verified the sequence accuracy for all mutational SNPs and pseudogenes in M66-2. Firstly we inspected the assembled PHRAP files in CONSED. For 97 variations found (67 mutational changes (Table 1) and 30 pseudogenes including 8 with a length of less than 300bp (see annotation section below)), there were 95 which have at least 2 fold sequence coverage from different clones, and the other one was confirmed by PCR. The trace files for the relevant regions were then inspected and compared with those of the homologous regions of strain O395. In every case the basis for the different base call(s) was evident and consistent in all trace files, giving very strong support for the polymorphisms.

For O395, the sequence was also verified as for M66-2. For 429 variations (371 mutational changes (Table 1), 58 pseudogenes including 21 of less than 300bp in length (see annotation section below)) all have a good sequence quality with least 10 fold coverage.

All of the sequence variations in M66-2 and O395 relative to each other or N16961 were confirmed, with no changes required to the original annotation.

Verification of N16961 SNPs

For N16961, we checked most variations using the independent sequences of the genes in the N16961 ORF clone collection (DQ772770-DQ776221, DQ899316-DQ899639), which were generated by the Harvard Institute of Proteomics, and also the unfinished MO10 genome. MO10 is an isolate of the O139 variant of the 7th pandemic, and therefore if MO10 and the Harvard sequences were the same but different to N16961, the N16961 sequence was treated as an error and corrected. For any other variation among the 3 sequences the N16961 region was resequenced. As a result 114 corrections were made to the N16961 sequence, as listed in Table S8. The corrections to the N16961 sequence had a significant impact on the numbers of SNPs and pseudogenes recorded in the M66-2 and N16961 lineages.

Annotation and analysis

Open reading frames from 30 amino acids in length were predicted using Glimmer 3.0[43] and verified manually using the annotation of N16961. Transfer RNA and ribosomal RNA genes were predicted using tRNAscan-SE[44] and by similarity to N16961 rRNA genes, respectively. Artemis [45] was used to collate data and facilitate annotation. Function predictions were based on BLASTP similarity searches against the Swiss-Prot protein database (http://www.ebi.ac.uk/swissprot/) and the clusters of orthologous groups (COG) database (www.ncbi.nlm.nih.gov/COG). Orthologous gene sets were identified by BLASTP Reciprocal Best Hits.

Pseudogenes were detected by comparing the genome sequences of M66-2, N16961 and O395 using the program Psi_Phi [46] and verified manually. Genes disrupted in all three strains were found if two neighbouring genes matched a single gene in the homology search against the nr database of GenBank. We also included the pseudogenes previously annotated in the N16961 genome. During the sequence verification, 39 of the annotated N16961 pseudogenes were found to be full genes after sequence correction. Of the remaining annotated pseudogenes, 26 are less than 300 bp in length, and most of them were annotated as hypothetical genes. For these we looked at sequence conservation in other *V. cholerae* strains and other *Vibrio* species. Twenty-one are not conserved among the *V. cholerae* strains examined and were removed from the list. A further 4 present in *V. cholerae* have no sequence homology to other *Vibrio* species and were also removed, as we are using 300 bp as the cutoff for an orf being a gene unless there is other evidence.

Only one, VC0124, has a homolog that can be found in *V. vulnificus*, *Vibrionales bacterium*, *Vibrio sp.* and *V. splendidus etc*. Therefore only VC0124 can be confirmed as a gene but it should not be a pseudogene. The sequences used comprised the uncompleted genome sequences of *V. cholerae* strains B33, NCTC 8457, 2740-80, MAK 757, MO10, AM-19226, MZO-2, MZO-3, V51, V52, 623-39 and RC385, *V. alginolyticus* 12G01, *V. angustum* S14, *V. harveyi* HY01, *V. parahaemolyticus* AQ3810, *Vibrio sp*. Ex25, *Vibrio sp*. MED222, *V. splendidus* 12B01 and *Vibrionales bacterium* SWAT-3, and the completed genome sequences of *V. fischeri* ES114, *V. harveyi* ATCC BAA-1116, *V. parahaemolyticus* RIMD 2210633, *V. vulnificus* CMCP6, *V. vulnificus* YJ016 (GenBank accession numbers are AAWE00000000, AAWD00000000, AAUT00000000, AAUS00000000, AAKF00000000, AATY00000000, AAWF00000000, AAUU00000000, AAKI00000000, AAKJ00000000, AAWG00000000, AAKH00000000, AAPS00000000, AAOJ00000000, AAWP00000000, AAWQ00000000, AAKK00000000, AAND00000000, AAMR00000000, AAZW00000000, NC_006840, NC_006841, NC_009783, NC_009784, NC_004603, NC_004605, NC_004459, NC_004460, NC_005139, and NC_005140 respectively).

Sequence comparisons of individual genes were done using MULTICOMP [47]. Synonymous and non-synonymous substitution rates (K_s and K_a) from orthologous gene pairs were calculated using a program provided by Li [48] and seqinR (http://pbil.univ-lyon1.fr/software/SeqinR/seqinr_home.php). Alignment of the whole genomes of M66-2, N16961 and O395 was done using Multi-LAGAN [49] and checked visually.

Assessing the possibility that the pseudogenes arose after isolation

We looked at the sequence data for MO10 as it is a derivative of the 7th pandemic (see main text) and pseudogenes present in their common ancestor should be present. For the 7 pseudogenes attributed to the N16961 lineage, 5 of the genes are present in the incomplete MO10 genome, of which 4 are pseudogenes identical to the N16961 pseudogenes (VC0284, VC0583. VCA0133 and VCA0979 (VCA0980)) and the other (VC0822 (VC0821)) is intact in MO10. There were no homologues of VC0258 (VC0255) and VC1620 in the current release of MO10 sequence so it is not known if they are absent or yet to be sequenced. We conclude that at least 4 and up to 6 of the 7 N16961 lineage pseudogenes arose prior to isolation of N16961.

Generation of alignment and SNP plot of the 3 genomes (Figure 4 and Figure S1)

Colinearity of the 3 genomes was determined using Artemis Comparison Tool (ACT) [50] (Figure 3). For alignment the major inversions in the 2 chromosomes were handled by changing the O395 order to that of N16961. Large indels were removed and the remaining genome sequences were aligned using Multi-LAGAN [49]. Most regions aligned well but some regions with small indels aligned poorly so were taken out for

alignment by ClustalW [51] and the output used to replace the corresponding regions. Scripts written in house were used to identify and characterise substitutions and to generate coordinates for plotting using GNUplot (http://www.gnuplot.info/) to generate Figure S1. Other information such as gene names was plotted similarly.

Distinguishing mutations and recombination

In an earlier study that included the 3 strains used in this study, we observed a distinction between genes that had undergone a single mutational change and those that had undergone recombination recognized by having 4 to 51 SNPs relative to other strains in the lineage under study [15]. Perhaps the first to make this distinction were Guttman and Dykhuizen [52] using sequences of 4 genes in 12 strains of *E. coli*. Five of the strains were closely related and their genes were either identical or quite different. They inferred that the differences the latter group of genes were due to recombination. In a genome scale study of 2 *Mycobacterium tuberculosis* strains, the same distinction was made between mutation and recombination for genes with one or many base changes, and the number of mutational changes was be used to infer a time since divergence[53], In other cases such as *Y. pestis*[54] only mutational changes were observed.

In our case the genome alignments revealed a pattern with regions having a much higher frequency of SNPs than over most of the genome, which we attribute to a combination of SNPs due to mutation and SNPs due to recombination. Many of the putative recombinant regions covered a number of genes and we needed to define the ends of putative recombinant segments, as the junctions generated during recombination are assumed to be random, and not related to the boundaries of genes. We therefore developed a formal approach to identification of recombinant segments on the assumption that SNPs can result from 2 classes of events: the first comprises mutations that accumulate over time and are assumed to be distributed randomly, and the other is recombination that can import a high substitution segment in one event, also assumed to occur randomly. Defining the segments between two adjacent SNPs as ISSs (Inter-SNP Segments), the distribution of ISSs around the genome is expected to follow an exponential distribution if all the observed SNPs are due to mutations that occur as a Poisson process. However, ISSs brought in by recombination events will disturb this distribution and form anomalous clusters of ISSs. Therefore, the overall distribution observed will have an excess of short ISSs due to recombination and will not follow exponential distribution. This excess of short ISSs may be removed to fit an exponential distribution if most parts of the genome have not been involved in recombination. A progressive exclusion of the short ISSs will allow one to find a cut-off value. The distribution of ISSs with length longer than a cut-off value is expected to fit an exponential distribution.

The insertion of a segment by homologous recombination generates a junction at each end. These junctions are also assumed to be distributed randomly with respect to the mutational SNPs, although of course the 2 junctions that bound a specific recombination event are not randomly distributed with respect to each other. The effect of this is that ISSs bounded by a mutational SNP and a recombinational SNP will be part of the random distribution of mutational SNPs, so no adjustment is needed in the analysis that follows. None of the above takes into account any effect of selection affecting survival of specific mutations or recombinant segments, but this is not expected to affect significantly the overall distributions.

Based on the principles stated above, we developed a 4-step process to identify recombinant segments. The genome alignment used included most of the consensus sequence, but excluded the CTX region in the large chromosome, the integron in the small chromosome, all regions present in only one genome, and also all rRNA regions, as SNPs within them can be generated by intra-genome exchange [22]. For each genome sequence we then extracted the positions of all SNPs being sites that are different from the consensus sequence and recorded the distances between adjacent SNPs (ISSs).

Step 1/ Determining the distribution of SNPs due to mutation. The SNPs due to mutation will be randomly distributed throughout the genome, while those due to recombination will be clustered in recombinant segments, as the only recombinant segments that we detect are those with a higher frequency of SNPs than in the rest of the genome. The ISS values (di $(0<i<N)$) were entered into a database according their order in the alignment. The mean ISS values are 1738.6, 1707.8 and 207.6 for the large chromosomes of M66-2, N16961 and O395, respectively, and 1618.5, 694.0 and 146.1, respectively for the small chromosomes. None of the 6 distributions fits the exponential distribution for the observed mean. This is as expected as a result of recombination. It is a property of an exponential distribution that the right hand tail of the distribution is itself an exponential distribution and as we anticipate that the larger ISSs will be bounded by mutational SNPs, we used this property to look for the cutoff value for the longer ISSs that fit an exponential distribution. The cut-off value was varied starting from 0 and increased with a step size of 1 bp. A statistical test procedure described by Schmidt [55] was used to evaluate the fit to an exponential distribution of the ISSs longer than the cut-off. The effective cut-off values are 825, 1138 and 967 for the small chromosomes of M66-2, N16961 and O395, respectively. We used the distribution of the right tail to deduce the exponential distributions for the 3 genomes, which had mean ISS lengths of 29414.5, 23437.8 and 4787.23 respectively. For the large chromosomes the cut-off values are 1181, 1237, 887, with mean lengths of 35471.7, 21067.9 and 4105.71 respectively. Thus for each of the 6 chromosomes we now have an exponential distribution that we treat as being due to random mutation (Figure S7).

Step 2/ Preliminary definition of recombinant regions. We treated each genome as a mosaic of segments and used a sliding window of length equal to the cut-off value described above, to distinguish the substitutions potentially imported by recombination. The window moves with a step size of 1 bp and if there are more than 2 substitutions within the window, they are provisionally assumed to be caused by recombination. Overlapping windows with substitutions attributed to recombination were assumed in the first instance to comprise a region imported in a single recombination event. In this way each genome is divided into segments attributed provisionally to recombination or mutation alternately, but still in need of refinement.

.

Step 3/ Refining the boundaries of recombinant regions. The MaxChi procedure[56,57], has been widely used to determine the boundaries of recombination events and we used it to refine the distinction between recombinant and mutational segments, as well as to test the homogeneity within each segment.

MaxChi assesses the distribution of SNPs within a region of length l and mutation number m, to determine if it can be cut into 2 segments with statistically different distributions, and if so the cutpoint k gives the maximum probability that the 2 parts have different distributions. This is the most probable site for a recombination event. The Chi-Square test used in MaxChi is unreliable if the number of SNPs is too low. Thus for segments of 10 or fewer SNPs the Fisher's exact test was used instead. We call this procedure MaxFisher.

The segments defined in step 2 are treated as a series N_1, N_2, N_3, … N_i, … N_n. A stepwise process is used to determine the border between each pair of adjacent segments. In each round of the process, we first determine if the distribution of the SNPs within the segment N_i is consistent with a random distribution, using MaxChi or MaxFisher as appropriate. If this gives a significant value (≤0.05) for dividing the segment we cut the segment into 2 segments (new N_i and N_{i+1}) at the cut-point (k=kmax), and repeat this step on the new N_i until N_i is no longer separated into 2. We then assess the border between the segments N_i and N_{i-1}. To do this we temporarily merge these 2 segments and examine the merged region as above. If the MaxChi/ MaxFisher value is not significant, we merge the 2 segments, but if it is significant, a new cut-point is set based on the cut-point found. This process is then performed on segment N_{i+1} and so on until the entire chromosome has been examined. This whole process is then repeated and the number and span of the segments may change. We repeated the process up to 10 times in initial testing and found that the result stabilizes after 6 rounds, so we used 6 rounds in our final analysis.

The segments are next judged to be recombinant or mutational by comparing the mean ISS within them with the cut-off value from step 1. Adjacent recombinant segments are amalgamated as we have no way of distinguishing between multiple recombination

events and events that bring in DNA that had undergone recombination in the donor. The ISSs in the refined recombinant segments in most cases are within the cut-off value. However there are exceptions for ISSs at the boundary of recombinant and mutational segments. These exceptions violate the rules used in Step 2, and an adjustment is made to the boundary of recombinant segment by moving the cut-point forward or backward by one SNP so that any such ISS with greater than the cut-off value is excluded and any smaller than the cut-off value included in the recombinant segment.

Step 4/ Defining putative ends for each recombinant segment. We now have divided the SNPs into those most probably due to mutation and those most probably due to recombination, but to estimate the proportion of the genome that has undergone recombination we need to define the boundaries of each recombinant segment. These boundaries will be located somewhere between the outer SNPs at each end of recombinant segments and the adjacent mutational SNPs. For each junction we use the mean distance between SNPs in the sub-segment at the end of the recombinant segment as defined by MaxChi/MaxFisher in step 3, and assume that in the donor chromosome, the SNP after the terminal SNP that was incorporated, was at that mean distance from the terminal incorporated SNP, and that the end of the segment incorporated was midway between the 2 SNPs. We therefore extended the recombinant segment at each end by a distance equal to half of the mean inter-SNP distance for the terminal component of the recombinant segment.

The final outcome is a total of 181 segments in the 3 strains attributed to recombination, with the boundaries set to the best estimate. The method is not biased in favour of recombination or mutation, but gives an estimate of which SNPs are due to recombination and which to mutation, starting with the assumption that both occur, except that no segment of 2 SNPs is considered recombinant. There is a considerable excess of such groups, but the pattern may be due to clustered mutation and we simply note the excess and that we have made an arbitrary decision to count as recombinant only segments with 3 or more substitutions. Except for the small genome of N16961, the distribution of all ISSs in designated mutational segments is consistent with an exponential distribution. However, short recombinant segments, or low divergence recombinant segments may still be present, but we are not able to identify them individually, or distinguish their presence from variation in mutation frequency around the chromosome. Indeed we stress that while we believe that the outcome is a good overview of the contributions of mutation and recombination to the variation among these strains, there will nonetheless be some cases where SNPs are misallocated.

Allocation of recombinant segments to specific genomes and assessment of the distribution of recombinant segments

Most differences among the genomes could be attributed to changes in the M66-2 or N16961 lineages or the O/NM divergence. However this was not possible where all three sequences were different, which was the case where there were recombinant segments in all 3 genomes. The genome sequences of 2740-80, a US Gulf strain related to N16961 (for phylogenetic position of the US gulf strains, see Salim *et al.* [15] Figure 1), and of V52, an O37 clinical isolate from the Sudan related to O395, were used to help determine if one of the regions is the original sequence. If in such a region, M66-2 has same sequence as US Gulf or V52, we say that M66-2 has the original sequence and if O395 is the same as 2740-80, then O395 has the original sequence. However, if O395 is the same as V52, it can not be excluded that the recombination occurred in the common ancestor of the O and V52, so it can not be shown to be the original sequence. Seventeen and 8 regions were resolved in the large and small chromosomes respectively but 2 regions in each chromosome remain unresolved.

We also assessed the distribution of recombinant segments by taking the mid point of each recombinant segment and using the statistical test procedure described by Schmidt [55] found that they were randomly distributed ($P > 0.05$) in the M and N genomes but not in the O395 genome where there were too few shorter distances between segments. However with 32% of the genome within recombinant segments, there will be some cases of overlap and these would be treated as a single event and distort the distribution, and this may be the reason for deviation from the Poisson distribution.

Supporting References

1. Linton RW (1940) The Chemistry and Serology of the Vibrios. Bacteriol Rev 4: 261-319.
2. Pollitzer R (1959) Cholera. Monograph Series 43 Geneva: World Health Organization: 147-158.
3. Felsenfeld O (1964) Present Status of the El Tor Vibrio Problem. Bacteriol Rev 28: 72-86.
4. Feeley JC, Pittman M (1963) Studies on the haemolytic activity of El Tor vibrios. Bull World Health Organ 28: 347-356.
5. Tanamal ST (1959) Notes on paracholera in Sulawesi (Celebes). Am J Trop Med Hyg 8: 72-78.
6. De Moor CE (1949) Paracholera (El Tor): Enteritis choleriformis El Tor van Loghem. Bull World Health Organ 2: 5-17.
7. Gan KH, Achja EM, Sukono, Roekmono, Warsa R (1958) First cases of paracholera El-Tor (enteritis choleriformis El Tor Van Loghem) in Java. Trop Geogr Med 10: 113-116.
8. Mukerjee S (1963) Problems of Cholera (El Tor). Am J Trop Med Hyg 12: 388-392.

9. Felsenfeld O (1966) A review of recent trends in cholera research and control. With an annex on the isolation and identification of cholera vibrios. Bull World Health Organ 34: 161-195.
10. Xiang SH, Hobbs M, Reeves PR (1994) Molecular analysis of the *rfb* gene cluster of a group D2 *Salmonella enterica* strain: evidence for its origin from an insertion sequence-mediated recombination event between group E and D1 strains. J Bacteriol 176: 4357-4365.
11. Karaolis DKR, Johnson JA, Bailey CC, Boedeker EC, Kaper JB, et al. (1998) A *Vibrio cholerae* pathogenicity island (VPI) associated with epidemic and pandemic strains. Proc Natl Acad Sci U S A 95: 3134-3139.
12. Mahillon J, Chandler M (1998) Insertion sequences. Microbiol Mol Biol Rev 62: 725-774.
13. Kapfhammer D, Blass J, Evers S, Reidl J (2002) *Vibrio cholerae* phage K139: complete genome sequence and comparative genomics of related phages. J Bacteriol 184: 6592-6601.
14. Allet B (1979) Mu insertion duplicates a 5 base pair sequence at the host inserted site. Cell 16: 123-129.
15. Salim A, Lan R, Reeves PR (2005) *Vibrio cholerae* pathogenic clones. Emerg Infect Dis 11: 1758-1760.
16. Olsvik O, Wahlberg J, Petterson B, Uhlen M, Popovic T, et al. (1993) Use of automated sequencing of polymerase chain reaction-generated amplicons to identify three types of cholera toxin subunit B in *Vibrio cholerae* O1 strains. J Clin Microbiol 31: 22-25.
17. Davis BM, Moyer KE, Boyd EF, Waldor MK (2000) CTX prophages in classical biotype *Vibrio cholerae*: functional phage genes but dysfunctional phage genomes. J Bacteriol 182: 6992-6998.
18. Davis BM, Waldor MK (2000) CTXphi contains a hybrid genome derived from tandemly integrated elements. Proc Natl Acad Sci U S A 97: 8572-8577.
19. Mekalanos JJ (1983) Duplication and amplification of toxin genes in *Vibrio cholerae*. Cell 35: 253-263.
20. Dziejman M, Balon E, Boyd D, Fraser CM, Heidelberg JF, et al. (2002) Comparative genomic analysis of *Vibrio cholerae*: genes that correlate with cholera endemic and pandemic disease. Proc Natl Acad Sci U S A 99: 1556-1561.
21. O'Shea YA, Finnan S, Reen FJ, Morrissey JP, O'Gara F, et al. (2004) The *Vibrio* seventh pandemic island-II is a 26.9 kb genomic island present in *Vibrio cholerae* El Tor and O139 serogroup isolates that shows homology to a 43.4 kb genomic island in *V. vulnificus*. Microbiology 150: 4053-4063.
22. Lan R, Reeves PR (1998) Recombination between rRNA operons created most of the ribotype variation observed in the seventh pandemic clone of *Vibrio cholerae*. Microbiology 144: 1213-1221.

23. Lin W, Fullner KJ, Clayton R, Sexton JA, Rogers MB, et al. (1999) Identification of a *vibrio cholerae* RTX toxin gene cluster that is tightly linked to the cholera toxin prophage. Proc Natl Acad Sci U S A 96: 1071-1076.
24. Rubin EJ, Lin W, Mekalanos JJ, Waldor MK (1998) Replication and integration of a *Vibrio cholerae* cryptic plasmid linked to the CTX prophage. Mol Microbiol 28: 1247-1254.
25. Barker A, Clark CA, Manning PA (1994) Identification of VCR, a repeated sequence associated with a locus encoding a hemagglutinin in *Vibrio cholerae* 01. J Bacteriol 176: 5450-5458.
26. Rowe-Magnus DA, Guerout AM, Biskri L, Bouige P, Mazel D (2003) Comparative analysis of superintegrons: engineering extensive genetic diversity in the Vibrionaceae. Genome Res 13: 428-442.
27. Boucher Y, Nesbo CL, Joss MJ, Robinson A, Mabbutt BC, et al. (2006) Recovery and evolutionary analysis of complete integron gene cassette arrays from *Vibrio*. BMC Evol Biol 6: 3.
28. Ruby EG, Urbanowski M, Campbell J, Dunn A, Faini M, et al. (2005) Complete genome sequence of *Vibrio fischeri*: a symbiotic bacterium with pathogenic congeners. Proc Natl Acad Sci U S A 102: 3004-3009.
29. Makino K, Oshima K, Kurokawa K, Yokoyama K, Uda T, et al. (2003) Genome sequence of *Vibrio parahaemolyticus*: a pathogenic mechanism distinct from that of *V. cholerae*. Lancet 361: 743-749.
30. Chen CY, Wu KM, Chang YC, Chang CH, Tsai HC, et al. (2003) Comparative genome analysis of *Vibrio vulnificus*, a marine pathogen. Genome Res 13: 2577-2587.
31. Tatusov RL, Fedorova ND, Jackson JD, Jacobs AR, Kiryutin B, et al. (2003) The COG database: an updated version includes eukaryotes. BMC Bioinformatics 4: 41.
32. Galperin MY, Nikolskaya AN, Koonin EV (2001) Novel domains of the prokaryotic two-component signal transduction systems. FEMS Microbiol Lett 203: 11-21.
33. Galperin MY (2004) Bacterial signal transduction network in a genomic perspective. Environ Microbiol 6: 552-567.
34. Jenal U, Malone J (2006) Mechanisms of cyclic-di-GMP signaling in bacteria. Annu Rev Genet 40: 385-407.
35. Beyhan S, Yildiz FH (2007) Smooth to rugose phase variation in *Vibrio cholerae* can be mediated by a single nucleotide change that targets c-di-GMP signalling pathway. Mol Microbiol 63: 995-1007.
36. Lim B, Beyhan S, Meir J, Yildiz FH (2006) Cyclic-diGMP signal transduction systems in *Vibrio cholerae*: modulation of rugosity and biofilm formation. Mol Microbiol 60: 331-348.
37. Wyckoff EE, Mey AR, Payne SM (2007) Iron acquisition in *Vibrio cholerae*. Biometals 20: 405-416.

38. Lan R, Stevenson G, Reeves PR (2003) Comparison of two major forms of the *Shigella* virulence plasmid pINV: positive selection is a major force driving the divergence. Infect Immun 71: 6298-6306.
39. Bina J, Zhu J, Dziejman M, Faruque S, Calderwood S, et al. (2003) ToxR regulon of *Vibrio cholerae* and its expression in vibrios shed by cholera patients. Proc Natl Acad Sci U S A 100: 2801-2806.
40. Gordon D, Abajian C, Green P (1998) Consed: a graphical tool for sequence finishing. Genome Res 8: 195-202.
41. Margulies M, Egholm M, Altman WE, Attiya S, Bader JS, et al. (2005) Genome sequencing in microfabricated high-density picolitre reactors. Nature 437: 376-380.
42. Kurtz S, Phillippy A, Delcher AL, Smoot M, Shumway M, et al. (2004) Versatile and open software for comparing large genomes. Genome Biol 5: R12.
43. Delcher AL, Bratke KA, Powers EC, Salzberg SL (2007) Identifying bacterial genes and endosymbiont DNA with Glimmer. Bioinformatics 23: 673-679.
44. Lowe TM, Eddy SR (1997) tRNAscan-SE: a program for improved detection of transfer RNA genes in genomic sequence. Nucleic Acids Res 25: 955-964.
45. Berriman M, Rutherford K (2003) Viewing and annotating sequence data with Artemis. Brief Bioinform 4: 124-132.
46. Lerat E, Ochman H (2004) Psi-Phi: exploring the outer limits of bacterial pseudogenes. Genome Res 14: 2273-2278.
47. Reeves PR, Farnell L, Lan R (1994) MULTICOMP: a program for preparing sequence data for phylogenetic analysis. Comput Appl Biosci 10: 281-284.
48. Li WH (1993) Unbiased estimation of the rates of synonymous and nonsynonymous substitution. J Mol Evol 36: 96-99.
49. Brudno M, Do CB, Cooper GM, Kim MF, Davydov E, et al. (2003) LAGAN and Multi-LAGAN: efficient tools for large-scale multiple alignment of genomic DNA. Genome Res 13: 721-731.
50. Carver TJ, Rutherford KM, Berriman M, Rajandream MA, Barrell BG, et al. (2005) ACT: the Artemis Comparison Tool. Bioinformatics 21: 3422-3423.
51. Thompson JD, Higgins DG, Gibson TJ (1994) CLUSTAL W: Impoving the sensitivity of progressive multiple sequence alignment through sequence weighting, position specifc gap penalties and weight matrix choice. Nucleic Acids Res 22: 4673-4680.
52. Guttman DS, Dykhuizen DE (1994) Clonal divergence in *Escherichia coli* as a result of recombination, not mutation. Science 266: 1380-1383.
53. Hughes AL, Friedman R, Murray M (2002) Genomewide pattern of synonymous nucleotide substitution in two complete genomes of Mycobacterium tuberculosis. Emerg Infect Dis 8: 1342-1346.
54. Achtman M, Morelli G, Zhu P, Wirth T, Diehl I, et al. (2004) Microevolution and history of the plague bacillus, *Yersinia pestis*. Proc Natl Acad Sci U S A 101: 17837-17842.

55. Schmidt KH (2004) A new test for random events of an exponential distribution. EUR PHYS J A 8: 141-145.
56. Smith JM (1992) Analyzing the mosaic structure of genes. J Mol Evol 34: 126-129.
57. Spencer M (2003) Exact significance levels for the maximum chi(2) method of detecting recombination. Bioinformatics 19: 1368-1370.
