# Supplementary material for: A Recalibrated Molecular Clock and Independent Origins for the Cholera Pandemic Clones
Source: PLoS One. 2008 Dec 30;3(12):e4053. doi: 10.1371/journal.pone.0004053 (PMC2605724; doi:10.1371/journal.pone.0004053)

Figure S1. Alignment of genomes of *V. cholerae* M66-2 (Makassar 1937 outbreak strain), N16961 (7th pandemic strain), O395 (6th pandemic outbreak strain). Large chromosome Panel 1

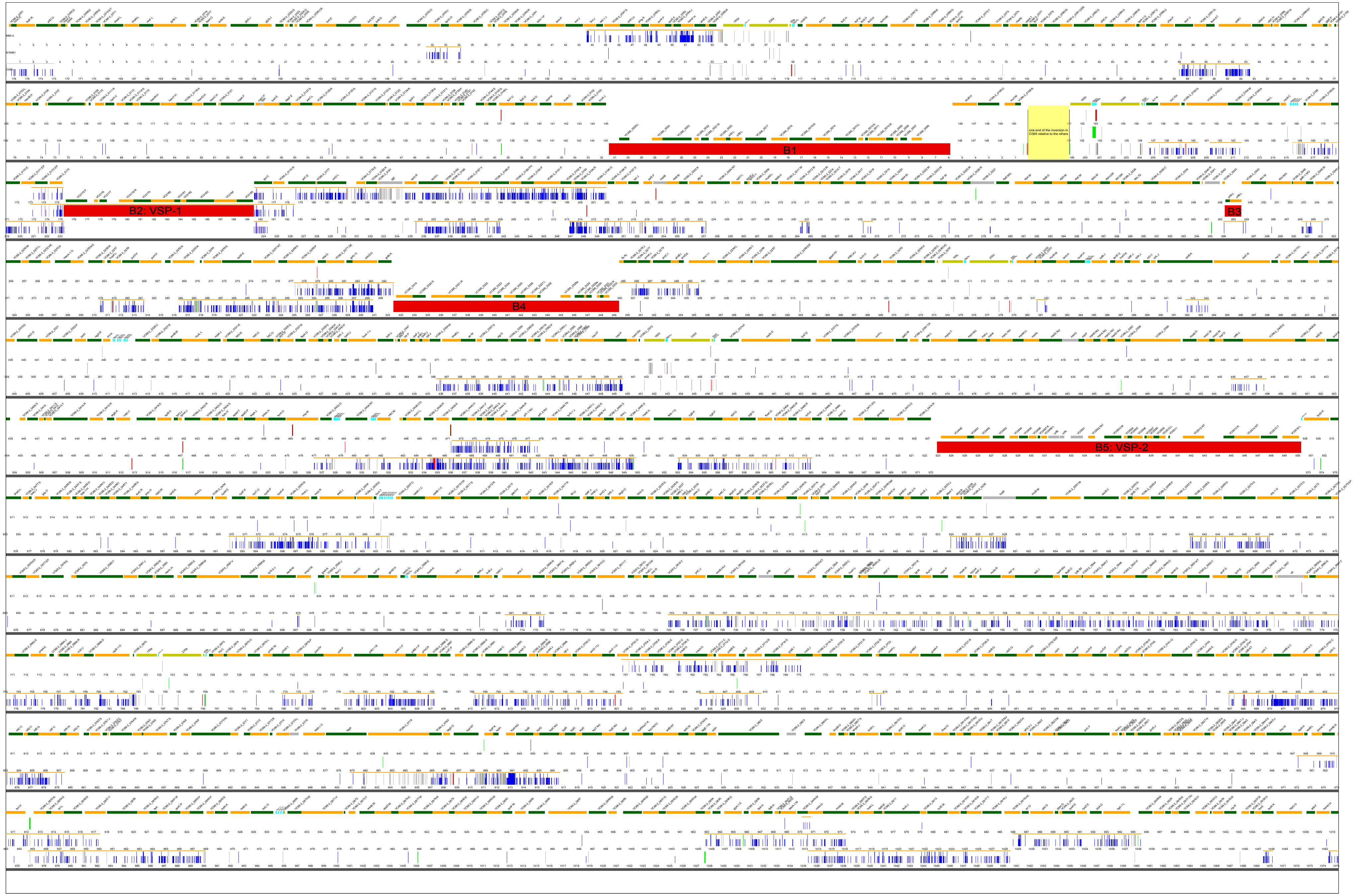

Figure S1. Alignment of genomes of *V. cholerae* M66-2 (Makassar 1937 outbreak strain), N16961 (7th pandemic strain), O395 (6th pandemic outbreak strain). Large chromosome Panel 2

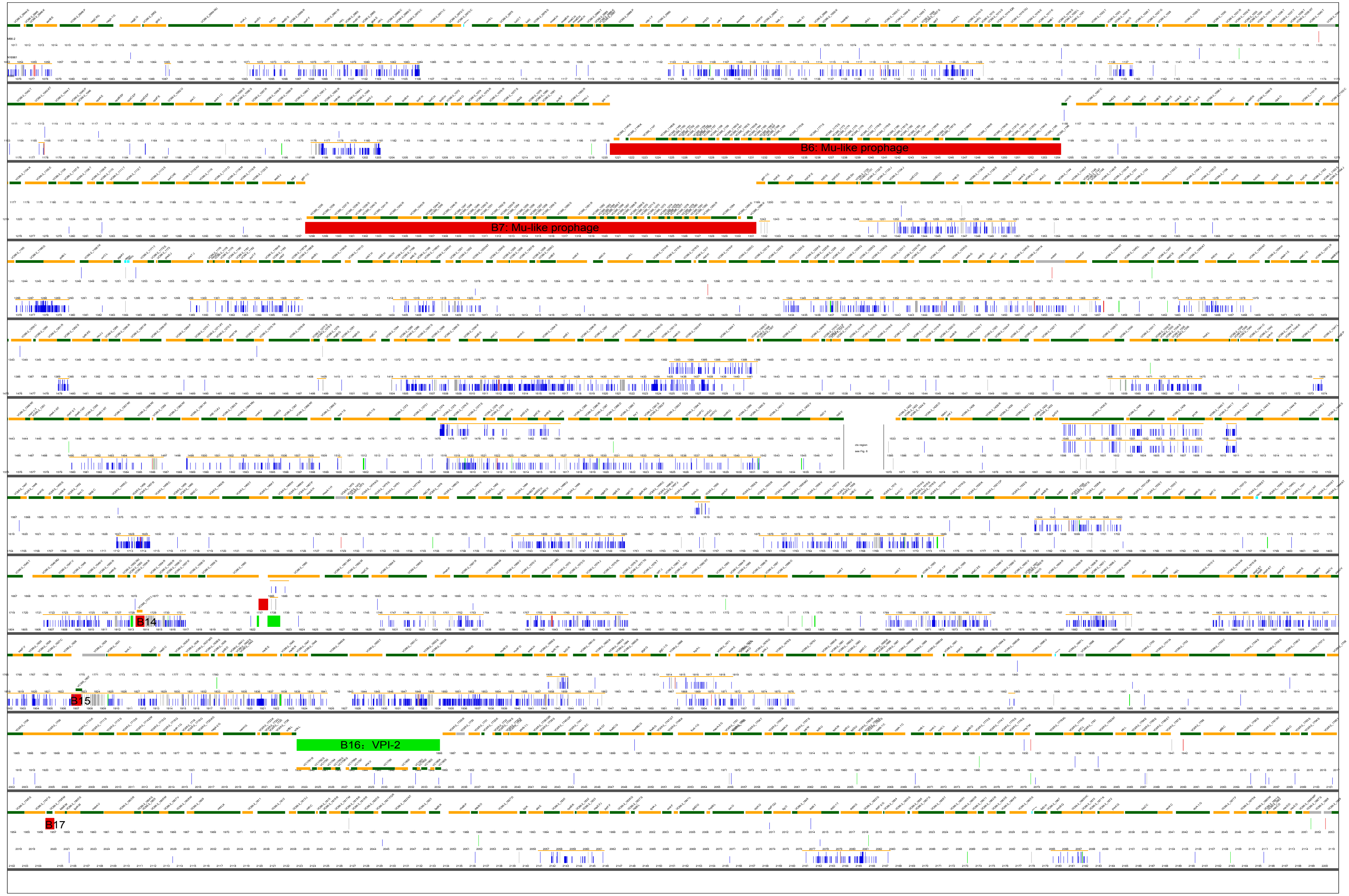

Figure S1. Alignment of genomes of *V. cholerae* M66-2 (Makassar 1937 outbreak strain), N16961 (7th pandemic strain), O395 (6th pandemic outbreak strain). Large chromosome Panel 3

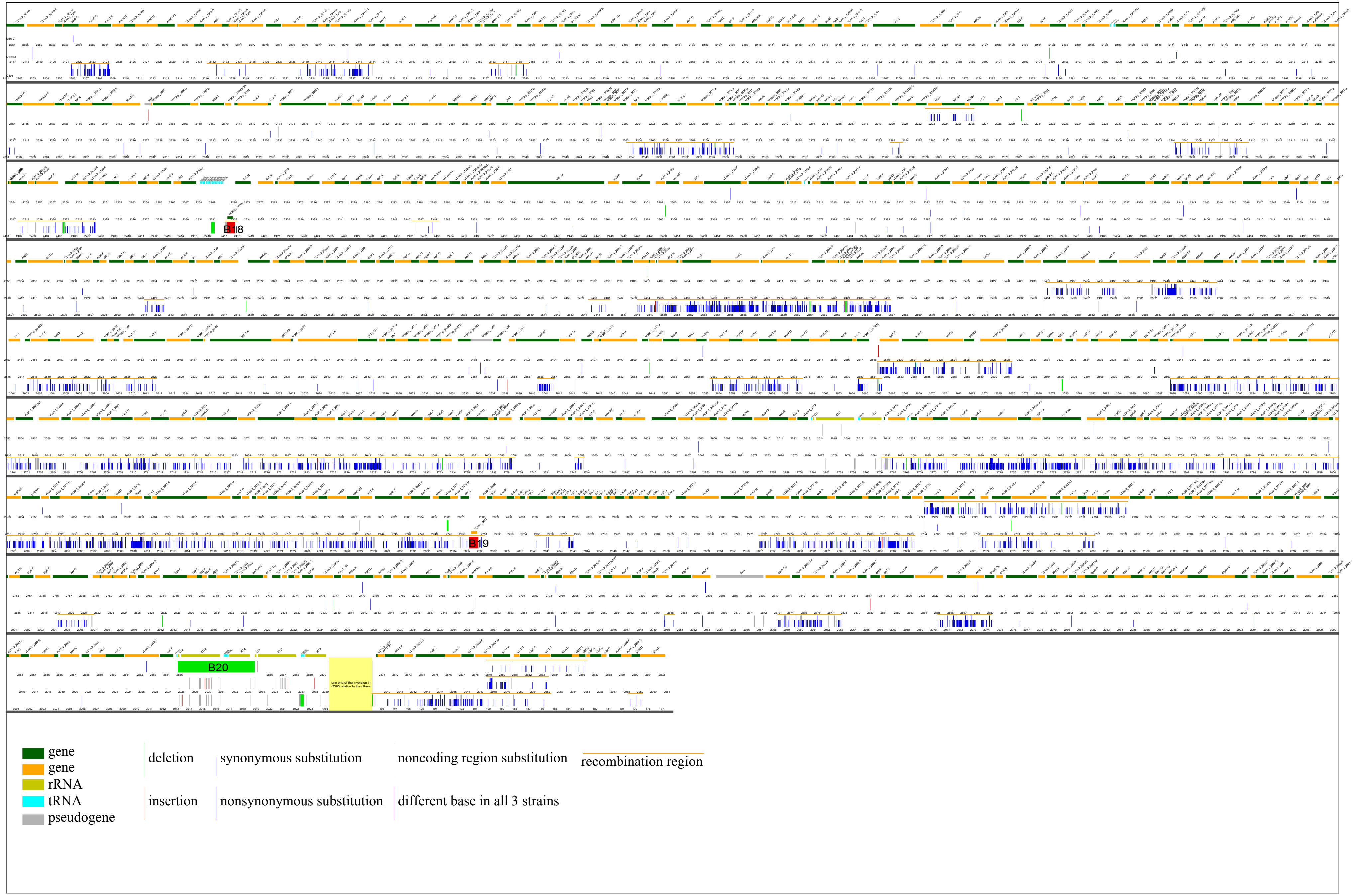

Figure S1. Alignment of genomes of *V. cholerae* M66-2 (Makassar 1937 outbreak strain), N16961 (7th pandemic strain), O395 (6th pandemic outbreak strain). Small chromosome

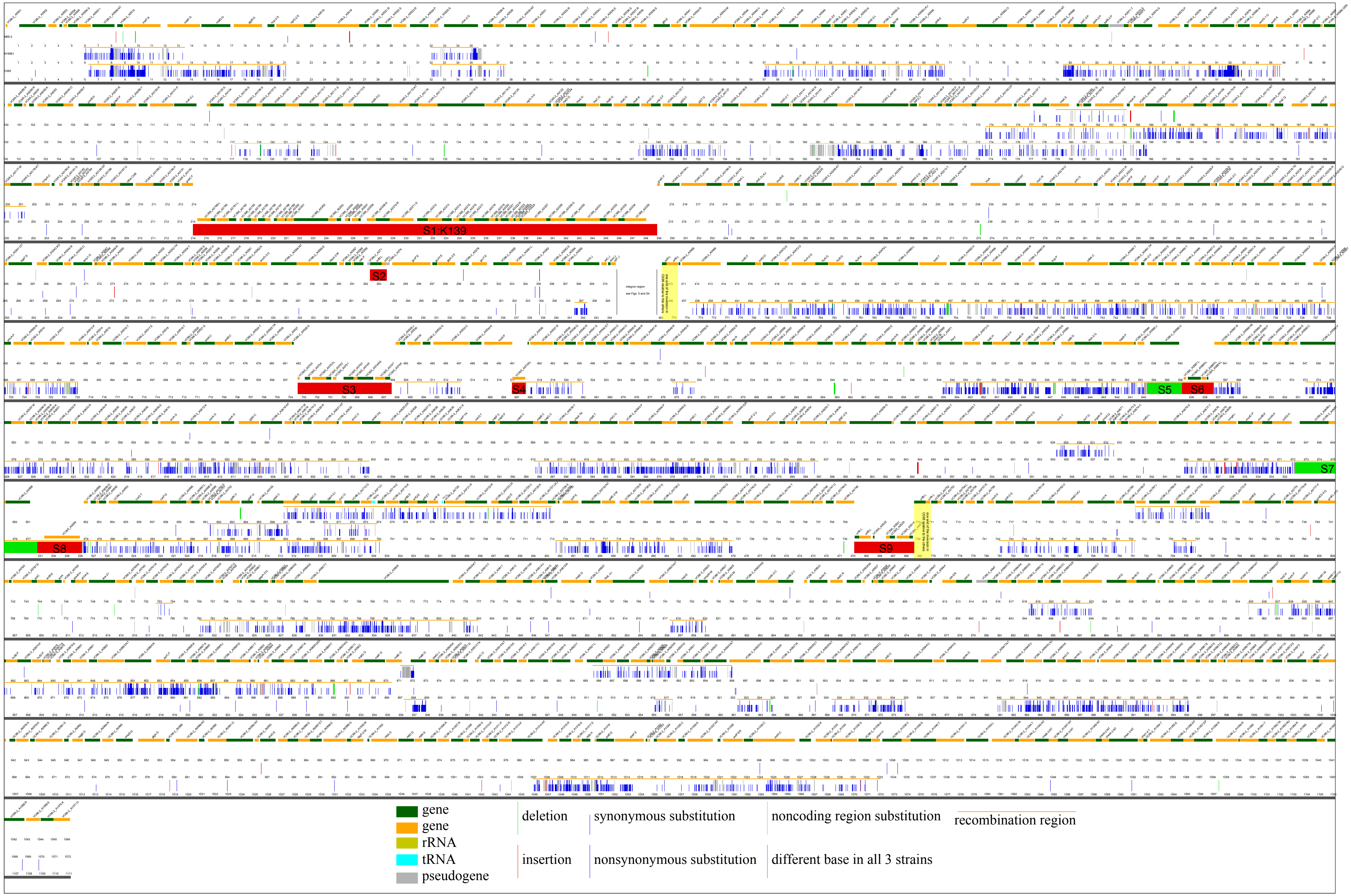

Figure S1. Plot of mutations, recombination events and indels in the genomes of strains M66-2, N16961 and O395.

The genomes of M66-2, N16961 and O395 were aligned as described in Supporting Methods. The large chromosome is presented in 3 pages and the small chromosome in 1 with 100 Kb per row. The maps are best viewed on screen zoomed in at appropriate magnification or printed at A0 size. The *ctx* region and the integron are described elsewhere and not included but their positions are marked. The boundaries of the segments inverted in O395 relative to M66-2 and N16961 are also marked. The 8 *rrn* gene sets are named a-h as in the GenBank annotation for N16961**.** Top: the genes for M66-2 with annotation. Below: 3 bands for the genomes of M66-2, N16961 and O395 respectively from top to bottom, each with genome map positions in kb. Large indels shown as red (insertion) or green (absence) blocks and named as in Table S4. Vertical lines mark sites where that genome differs from the others: blue, base substitution in gene (two thirds height, synonymous substitutions; full height, nonsynonymous substitutions); grey, base substitution in pseudogene or non-coding region; red, base present; green, base absent; purple, different base in all 3 genomes. Orange line above group of base difference markers shows segment inferred to have undergone recombination. The grey lines separate sections of the alignment.

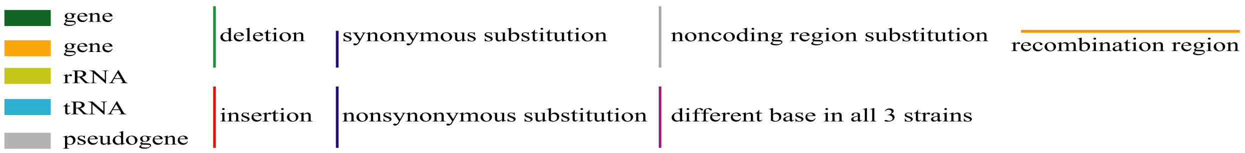

Supplement: Figure S1 — Plot of mutations, recombination events and indels in the genomes of strains M66-2, N16961 and O395 (6.69 MB PDF) [file pone.0004053.s002.pdf]
