# Supplementary material for: A Recalibrated Molecular Clock and Independent Origins for the Cholera Pandemic Clones
Source: PLoS One. 2008 Dec 30;3(12):e4053. doi: 10.1371/journal.pone.0004053 (PMC2605724; doi:10.1371/journal.pone.0004053)
Supplement: Figure S2 — Histogram of the lengths of the recombinant segments (0.29 MB PDF) [file pone.0004053.s003.pdf]

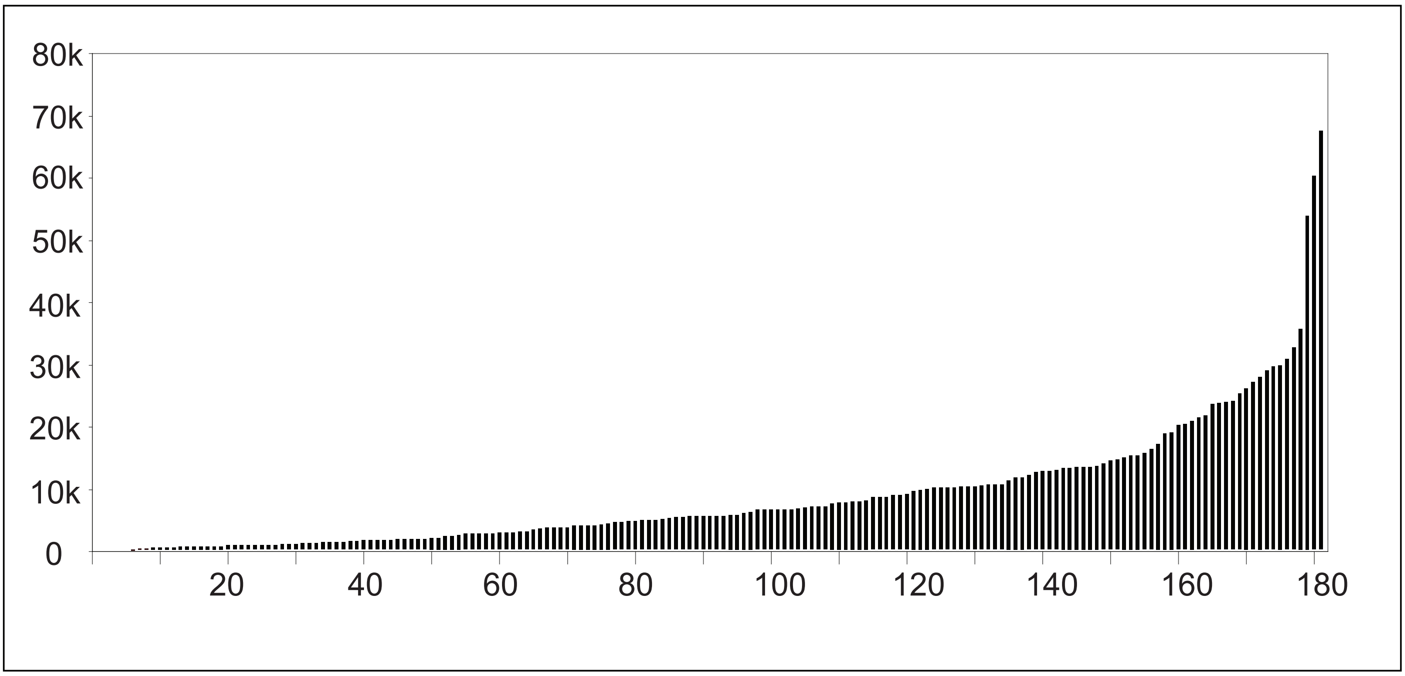

Figure S2. Histogram of the lengths of recombinant segments.

The 181 recombinant segments in the 3 genomes, determined as described in Supporting Methods, were plotted in the figure. Each column represents one recombinant segment, sorted from the smallest to the largest by size. Y-axis is the size of recombinant segments.
