# Supplementary material for: A Recalibrated Molecular Clock and Independent Origins for the Cholera Pandemic Clones
Source: PLoS One. 2008 Dec 30;3(12):e4053. doi: 10.1371/journal.pone.0004053 (PMC2605724; doi:10.1371/journal.pone.0004053)
Supplement: Figure S3 — Comparison of the 3 integrons using artemis comparson tool and dot plots (0.58 MB PDF) [file pone.0004053.s004.pdf]

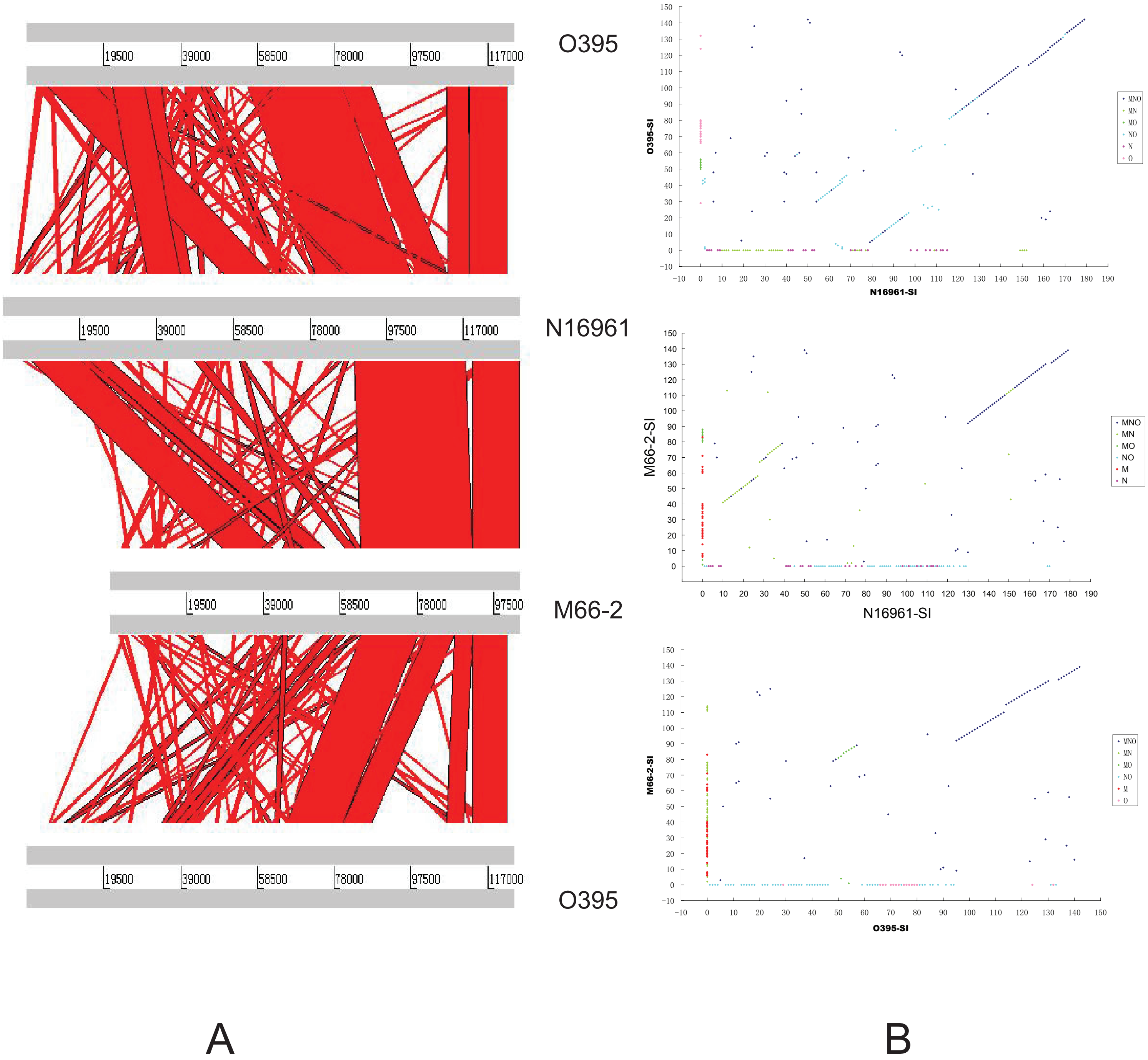

Figure S3. Comparison of the 3 integrons using artemis comparison tool and dot plots.

(A) Graphic representation of homologies of the integrons of M66-2, N16961 and O395 based on pairwise BLASTN searches, depicted using the Artemis comparison tool. The blocks shown in Figure 5 and Figure S4 can be seen. **(B)** Pairwise comparisons of cassettes in the intgrons of strains M66-2, N16961 and O395. In each comparison cassettes that are present in only one of the pair are indicated by a coloured symbol against the axis for that strain, whereas those identical or near identical in the 2 strains are shown as a symbol in the body of the figure at a point projected from the 2 axes. Cassettes present in more than one copy show as symbols on a line(s) projecting from one (or both) of the axes. The colour code is the same for the 3 panels. The numbers on the axis are cassette numbers.
