# Supplementary material for: A Recalibrated Molecular Clock and Independent Origins for the Cholera Pandemic Clones
Source: PLoS One. 2008 Dec 30;3(12):e4053. doi: 10.1371/journal.pone.0004053 (PMC2605724; doi:10.1371/journal.pone.0004053)
Supplement: Figure S4 — Relationships of cassettes in the integrons of strains M66-2, N16961 and O395 (0.31 MB PDF) [file pone.0004053.s005.pdf]

Integron cassettes of M66-2 and their relationships Integron cassettes of N16961 and their relationships Integron cassettes of O395 and their relationships

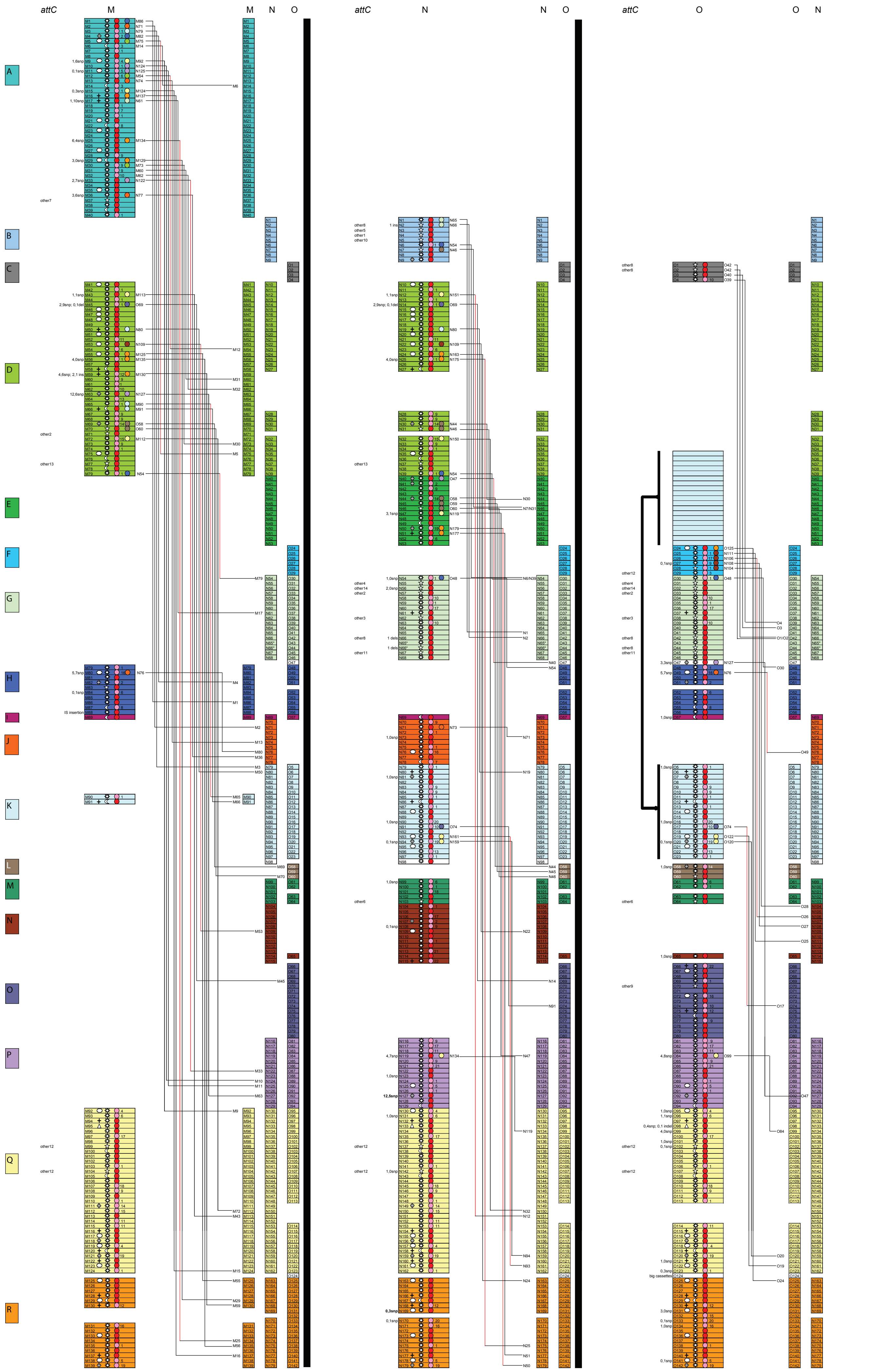

Figure S4. Relationships of cassettes in the integrons of strains M66-2, N16961 and O395.

Note that Figure 5 gives an overview of the cassette copying discussed in this legend, and the 2 figures can be usefully used in conjunction.

The 3 sections relate to the properties and relationships of cassettes of M66-2, N16961 and O395 respectively from left to right, that are represented by a column named M66-2 cassettes etc, in each section. The cassettes are numbered in map order for each strain with information about each cassette. Cassettes present in 2 or 3 of the strains are aligned, and as can be seen there is considerable conservation of order, but many cassettes are not present in all 3 genomes, as indicated by gaps in the alignment. The gaps define blocks of cassettes with different distributions, named A-R as shown to the left of the figure, and the cells for cassettes in each block are filled with a different colour. Only for block K (cassettes O5-O23) does the order vary, and for O395 these cassettes are shown against homologues in N16961, with a "shadow" of the cassettes in their true position connected by a double headed arrow to indicate presumed translocation. There are a total of 271 unique cassettes of which 139, 179 and 142 are present in M66-2, N16961 and O395 respectively. Within the cell for each cassette are 3 or 4 symbols as described below and/or in the key at the bottom of the figure. To the right of each section are subsidiary copies of 2 or 3 of the cassette columns as required to indicate the cassette relationships discussed below. For those cassettes present in 2 copies that are identical or near identical, lines emanate from the upstream cassette in one of the major columns, and connect to the downstream member of the pair in one of the subsidiary columns. These connections represent the proposed copying of the downstream cassette during addition of the upstream member by IntI, thereby indicating the putative origins of those cassettes in the major columns. Every 5th line is coloured red. We have to include all three genomes to cover the putative donors for M66-2 cassettes, whereas for N16969 and O395 all putative donor cassettes are present in one or both of N16961 and O395. The presumptive donor cassette is named to the right of the new cassette, and to the left is given the number of base differences (if any) between donor and copy in the non-*attC* and *attC* components respectively of the cassette, separated by a comma. Insertions etc are also indicated. We propose that where the donor cassette is not present in the strain with the upstream cassette, that the parent cassette has since been lost in that lineage. The right hand set of symbols in the cassettes is for cassettes that have been copied and are circles coloured to indicate the block for the source cassette. For cassettes present 3 or more times, the colour is based on the copy nearest the 3' end of the integrons which is treated as origin for all others. However the lines connecting new and source cassettes are made to minimise copying from cassettes that have since been lost. For example in the case of N30, we have treated the ultimate source as O58 (block L) although the line connects it to N44 (block D): however N44 is proposed to be derived from O54 which must be the ultimate source regardless or wether it was copied directly or via N44. Other symbols in the cassettes are described below. The integron regions cover bp 310949-409046 (M66-2), 309750-435031 (N16961) and 344619-463728 (O395).

Symbols:

toxin/antitoxin cassettes — Cassette includes gene with pathogenicity — Cassette includes antibiotic resistance gene — Cassette includes gene for other enzyme

cluster I *V. cholerae attC* — cluster II *V. cholerae attC* — *attC* other than that of cluster I and II, with division into 14 groups based on distribution in a NJ tree. The numbers in *attC* cells indicate the group number

Cassette is in one of 22 groups, which have 2 or more related cassetes forms (see Table S4) - There may also be other copies of this cassette attributed to copying within the lineage

Cassette not in a group with 2 or more related cassettes forms - there may be other copies of this cassette attributed to copying within the lineage

Note:

1. Coloured circles on the right-hand side in the cassette column indicate the origin of the cassette, each being the colour of the donor block.
2. The column immediately to the left of the cassettes gives the SNP differences between the cassette and its most closely related cassette with the second number being differences in attC and the 1st number being differences in the rest of the cassette.
