# Supplementary material for: A Recalibrated Molecular Clock and Independent Origins for the Cholera Pandemic Clones
Source: PLoS One. 2008 Dec 30;3(12):e4053. doi: 10.1371/journal.pone.0004053 (PMC2605724; doi:10.1371/journal.pone.0004053)
Supplement: Figure S5 — Function Categories (COGs) of genes with or without mutational changes (0.05 MB PDF) [file pone.0004053.s006.pdf]

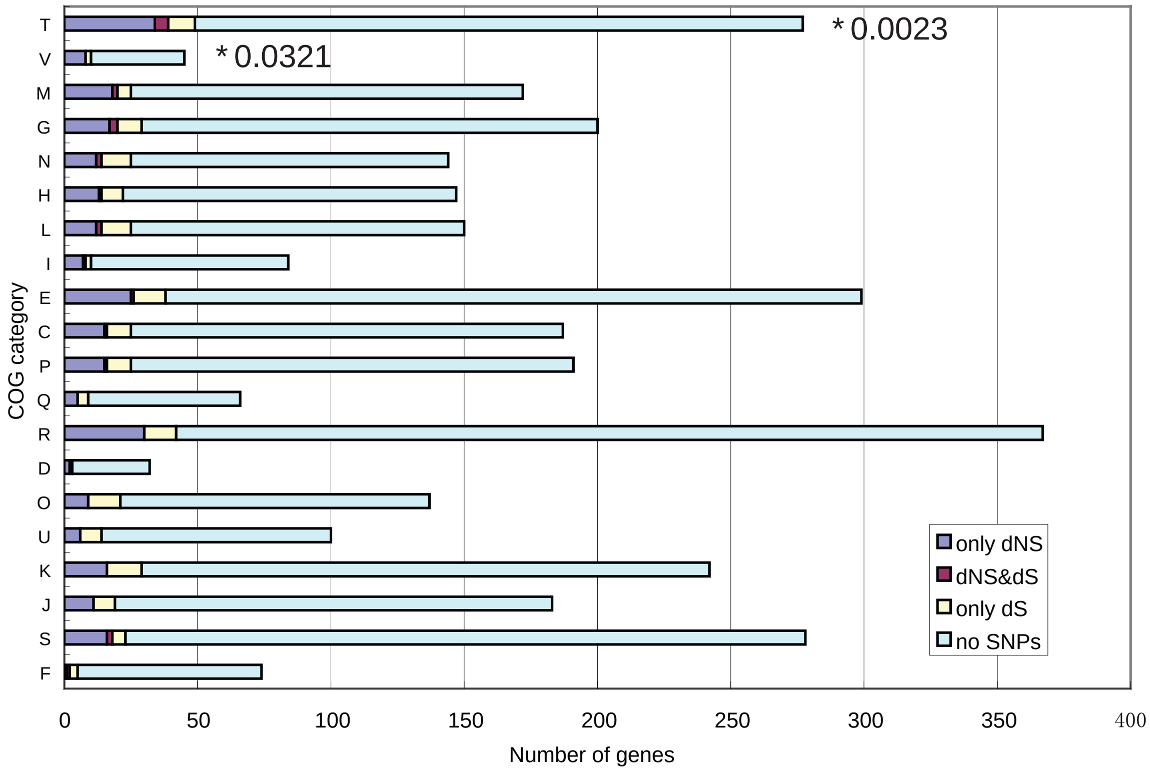

Figure S5. Function Categories (COGs) of genes with or without mutational changes.

Genes from non-recombination regions were categorised by Clusters of Orthologous Groups (COG) to determine whether any particular COG has significantly more genes that have undergone change. SNP-carrying genes were divided into 3 categories: synonymous SNPs only (only dS), non-synonymous only (only dNS), and both types of SNPs (dNS&dS). The number of genes carrying no SNPs in each COG was also plotted. COGs A and B were excluded as there is only one gene in each category and only one SNP in one gene. Genes not in COGs were also excluded. The categories marked with a star indicate that the COG is significantly overrepresented ($p < 0.05$, Z test) when genes carrying any non-synonymous SNPs were tested for their distribution among the COGs. The numbers at the end of the bars are raw p values. When the Bonferroni correction for multiple tests (20 tests for 20 categories) was applied, only category T is significant at the corrected P value ($p < 0.0023$). The COG functional categories are as follows: A, RNA processing and modification; B, Chromatin structure and dynamics; C, Energy production and conversion; D, Cell cycle control, cell division, chromosome partitioning; E, Amino acid transport and metabolism; F, Nucleotide transport and metabolism; G, Carbohydrate transport and metabolism; H, Coenzyme transport and metabolism; I, Lipid transport and metabolism; J, Translation, ribosomal structure and biogenesis; K, Transcription; L, Replication, recombination and repair; M, Cell wall/membrane/envelope biogenesis; N, Cell motility; O, Posttranslational modification, protein turnover, chaperones; P, Inorganic ion transport and metabolism; Q, Secondary metabolites biosynthesis, transport and catabolism; R, General function prediction only; S, Function unknown; T, Signal transduction mechanisms; U, Intracellular trafficking, secretion, and vesicular transport; V, Defence mechanisms; W, Extracellular structures; Y, Nuclear structure; Z, Cytoskeleton.
