# Supplementary material for: A Recalibrated Molecular Clock and Independent Origins for the Cholera Pandemic Clones
Source: PLoS One. 2008 Dec 30;3(12):e4053. doi: 10.1371/journal.pone.0004053 (PMC2605724; doi:10.1371/journal.pone.0004053)
Supplement: Figure S6 — Correlation of gene expression level with ratio of synonymous and non-synonymous substitution rates (0.46 MB PDF) [file pone.0004053.s007.pdf]

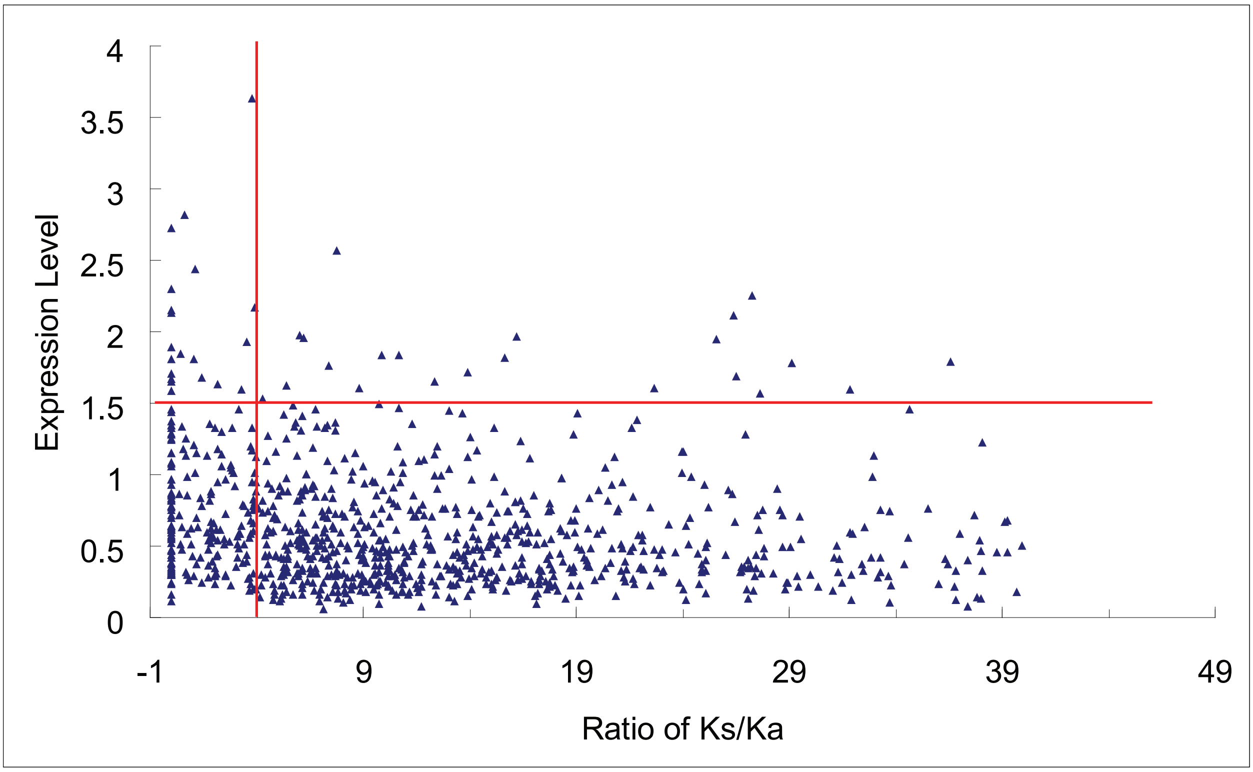

Figure S6. Correlation of gene expression level with ratio of synonymous and non-synonymous substitution rates.

Synonymous (Ks) and non-synonymous (Ka) substitution rates were determined for each gene from recombinant regions (Table 1). The *in vivo* data was from Bina et al. [1]. Statistically significant correlation was found between Ks/Ka and expression level (Spearman rank correlation, r= -0.146, P = 0.0001). Genes were further divided into 2 groups by the expression level (above and below 1.5) and the Ks/Ka ratios (< 4.0 and >= 4.0) as shown by the horizontal and vertical lines respectively. The division is statistically significant (P = 0.027, Fisher's exact test).

1. Bina J, Zhu J, Dziejman M, Faruque S, Calderwood S, et al. (2003) ToxR regulon of *Vibrio cholerae* and its expression in vibrios shed by cholera patients. Proc Natl Acad Sci U S A 100: 2801-2806.
