# Supplementary material for: A Recalibrated Molecular Clock and Independent Origins for the Cholera Pandemic Clones
Source: PLoS One. 2008 Dec 30;3(12):e4053. doi: 10.1371/journal.pone.0004053 (PMC2605724; doi:10.1371/journal.pone.0004053)
Supplement: Figure S7 — Distribution of Inter-SNP segment (ISS) lengths for the 6 chromosomes (0.37 MB PDF) [file pone.0004053.s008.pdf]

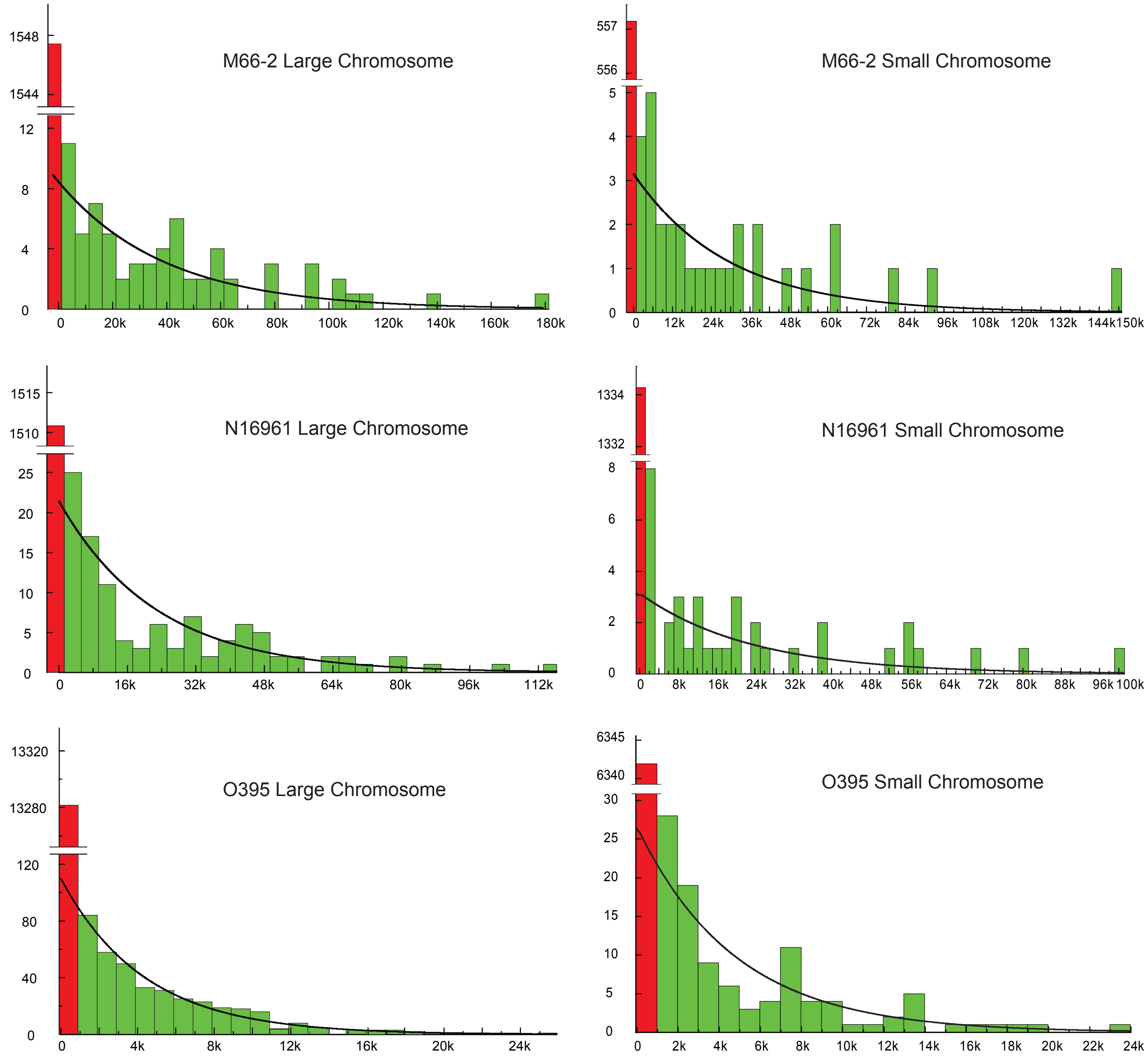

Figure S7. Distribution of Inter-SNP segment (ISS) lengths for the 6 chromosomes.

In the top left panel for the large chromosome of M66-2, the first bin (red) extends from 1 to 1,181 base pairs, the cut-off point discussed in the text. The remaining bins (green) have a range of 5000. The function plotted is an exponential distribution based on the ISSs of length greater than the cut-off. It can be seen that it is a good fit to the ISSs above the cut-off, but that those below the cut-off do not fit at all. Note that the first bin has the width exaggerated, but the true width is apparent from the scale. The remaining panels have the comparable data for the other chromosomes as indicated using the appropriate cut-off values and suitable bin sizes.
