# Supplementary material for: A Recalibrated Molecular Clock and Independent Origins for the Cholera Pandemic Clones
Source: PLoS One. 2008 Dec 30;3(12):e4053. doi: 10.1371/journal.pone.0004053 (PMC2605724; doi:10.1371/journal.pone.0004053)
Supplement: Table S1 — General features of the M66-2, N16961 and O395 genomes (0.02 MB PDF) [file pone.0004053.s009.pdf]

Table S1. General features of the M66-2, N16961 and O395 genomes

	M66-2		N16961		O395	
	Large chromosome	Small chromosome	Large chromosome	Small chromosome	Large chromosome	Small chromosome
Size (bp)	2,892,523	1,046,382	2,961,168	1,072,316	3,024,078	1,111,222
G+C content (%)	47.83	47.04	47.7	46.91	47.78	46.87
Coding sequences	2,669	1,052	2,737	1,095	2,841	1,134
Pseudogenes*	19 (0.7)	9 (0.9)	16 (0.6)	10 (0.9)	24 (0.8)	17 (1.5)
Coding percentage	88.6	86.9	88.2	86.3	87.5	85.0
Average gene size (bp)	967	872	960	852	942	846
rRNA operons	7	0	8	0	8	0
tRNA	93	4	94	4	92	4

* Number in brackets is percentage
