# Supplementary material for: A Recalibrated Molecular Clock and Independent Origins for the Cholera Pandemic Clones
Source: PLoS One. 2008 Dec 30;3(12):e4053. doi: 10.1371/journal.pone.0004053 (PMC2605724; doi:10.1371/journal.pone.0004053)
Supplement: Table S2 — Allocation of SNPs in the O/MN divergence to either O395 or MN lineage (0.17 MB PDF) [file pone.0004053.s010.pdf]

Table S2. Allocation of SNPs in the O/MN divergence to either O395 or MN lineage.

O395 gene	location	S/NS /NC	MZO-3	MZO-2	AM19226	RC385	1587	consensus
VC395_A0002	2348	s	O	O	O	O	O	O
VC395_A0280	298841	s	O	O	O	O	O	O
VC395_A0297	313986	s	O	O	O	-	O	O
VC395_A0306	322000	s	O	O	O	-	O	O
VC395_A0607	557429	s	O	O	O	O	O	O
VC395_A0607	555374	s	O	O	O	O	O	O
VC395_A0857	817127	s	O	O	O	O	O	O
VC395_A0859	819725	s	O	O	O	O	O	O
VC395_A0878	844033	s	O	O	O	-	O	O
VC395_A0921	889869	s	O	O	O	O	O	O
VC395_A0929	897574	s	O	O	O	O	O	O
VC395_A0951	919454	s	O	O	O	-	O	O
VC395_A0974	939058	s	O	O	O	O	O	O
VC395_A0997	958191	s	O	O	**MN**	O	O	O
VC395_A1004	966730	s	O	O	O	-	O	O
VC395_A1042	1005170	s	O	O	O	O	O	O
VC395_A1049	1013120	s	O	O	O	O	**3**	O
VC395_A1078	1043454	s	O	O	O	O	O	O
VC395_0172	171693	s	-	O	O	O	O	O
VC395_0158	156171	s	O	O	O	O	O	O
VC395_0139	137704	s	O	O	O	O	O	O
VC395_0050	52426	s	O	O	O	O	O	O
VC395_0041	43631	s	O	O	O	O	O	O
VC395_0029	34756	s	-	O	-	-	O	O
VC395_0222	229475	s	O	O	O	O	O	O
VC395_0222	230090	s	O	O	O	O	O	O
VC395_0440	471364	s	O	O	O	O	O	O
VC395_0448	480449	s	O	O	O	O	O	O
VC395_0467	503276	s	O	O	O	O	O	O
VC395_0488	522369	s	O	O	O	-	O	O
VC395_0568	606686	s	O	O	O	O	O	O
VC395_0582	620728	s	O	O	O	-	O	O
VC395_0584	623579	s	O	O	O	-	O	O
VC395_0632	672303	s	O	O	O	O	O	O

VC395_0648	695151	s	O	O	O	-	O	O
VC395_0796	858471	s	O	O	O	O	O	O
VC395_0885	954697	s	O	O	O	O	O	O
VC395_0885	957311	s	O	O	O	O	O	O
VC395_0894	962855	s	-	O	O	O	-	O
VC395_0952	1025291	s	O	O	O	O	O	O
VC395_1035	1113635	s	O	O	O	O	O	O
VC395_1079	1154234	s	O	O	O	O	O	O
VC395_1111	1187122	s	O	O	O	O	O	O
VC395_1218	1279652	s	O	O	O	O	O	O
VC395_1333	1382154	s	O	O	O	O	-	O
VC395_1369	1411808	s	O	O	**MN**	O	O	O
VC395_1373	1415012	s	O	O	O	O	O	O
VC395_1478	1538318	s	O	O	O	O	O	O
VC395_1504	1567456	s	O	O	O	O	O	O
VC395_1567	1635944	s	O	**3**	**3**	O	O	O
VC395_1606	1676903	s	O	O	O	O	O	O
VC395_1970	2078776	s	O	-	O	O	O	O
VC395_1983	2096313	s	O	O	O	O	O	O
VC395_1987	2101084	s	O	**MN**	O	O	O	O
VC395_1999	2113341	s	O	O	O	O	O	O
VC395_2035	2155187	s	O	-	O	-	O	O
VC395_2109	2232707	s	O	O	O	-	O	O
VC395_2165	2292390	s	O	O	O	O	O	O
VC395_2174	2298841	s	O	O	O	O	O	O
VC395_2177	2301139	s	O	O	O	O	O	O
VC395_2177	2301520	s	O	O	O	O	O	O
VC395_2207	2337732	s	O	O	O	O	O	O
VC395_2210	2341176	s	O	O	O	O	O	O
VC395_2255	2380619	s	O	O	O	O	O	O
VC395_2325	2442995	s	**MN**	O	O	O	O	O
VC395_2343	2466656	s	O	O	O	O	O	O
VC395_2345	2467886	s	O	O	O	O	O	O
VC395_2361	2482570	s	O	O	O	O	O	O
VC395_2377	2501849	s	O	O	O	-	O	O
VC395_2418	2541104	s	O	O	O	O	O	O
VC395_2475	2601087	s	O	O	O	O	O	O

VC395_2488	2619826	s	O	O	O	3	O	O
VC395_2589	2740357	s	O	O	O	-	O	O
VC395_2601	2752006	s	O	O	O	O	O	O
VC395_2704	2846866	s	-	O	O	-	O	O
VC395_2706	2847673	s	-	O	O	-	O	O
VC395_2751	2896770	s	O	O	O	O	O	O
VC395_2828	2976298	s	O	O	O	O	O	O
VC395_2838	2984901	s	O	O	O	O	O	O
VC395_A0240	254524	s	-	-	-	-	-	O/MN
VC395_0137	135897	s	**MN**	**MN**	**O**	**O**	**MN**	O/MN
VC395_0855	923148	s	-	-	-	-	-	O/MN
VC395_0855	923547	s	-	-	-	-	-	O/MN
VC395_1878	1990241	s	-	-	-	-	-	O/MN
VC395_1884	2002031	s	-	-	-	-	-	O/MN
VC395_2491	2623770	s	**MN**	**O**	**MN**	**MN**	**O**	O/MN
VC395_1920	2033962	s	-	-	-	-	-	O/MN
VC395_1914	2029749	s	-	-	-	-	-	O/MN
VC395_A0283	300385	s	MN	MN	MN	MN	MN	MN
VC395_A0284	302951	s	MN	MN	MN	MN	MN	MN
VC395_A0318	335566	s	-	-	MN	-	-	MN
VC395_A0595	542542	s	MN	MN	MN	MN	MN	MN
VC395_0164	162722	s	MN	MN	MN	MN	MN	MN
VC395_0289	298282	s	MN	MN	MN	-	MN	MN
VC395_0377	402257	s	MN	MN	MN	MN	MN	MN
VC395_0397	425416	s	MN	MN	MN	-	MN	MN
VC395_0458	490886	s	MN	MN	MN	MN	MN	MN
VC395_0469	505401	s	MN	MN	MN	MN	MN	MN
VC395_0641	686081	s	MN	MN	MN	MN	MN	MN
VC395_0871	944007	s	MN	MN	MN	MN	MN	MN
VC395_0910	975691	s	MN	MN	MN	MN	MN	MN
VC395_0946	1018619	s	**O**	MN	MN	MN	MN	MN
VC395_1117	1194406	s	MN	MN	MN	-	MN	MN
VC395_1230	1292741	s	MN	MN	MN	-	MN	MN
VC395_1375	1418354	s	-	**O**	MN	MN	MN	MN
VC395_1706	1787402	s	MN	MN	MN	MN	MN	MN
VC395_2110	2234032	s	MN	MN	MN	3	MN	MN
VC395_2153	2280379	s	MN	MN	MN	MN	MN	MN

VC395_2172	2297710	s	MN	MN	MN	MN	MN	MN
VC395_2237	2362083	s	MN	MN	MN	MN	MN	MN
VC395_2339	2462075	s	MN	MN	MN	MN	MN	MN
VC395_2360	2481853	s	MN	MN	MN	MN	MN	MN
VC395_2522	2662576	s	MN	MN	MN	MN	MN	MN
VC395_2532	2677206	s	MN	MN	MN	MN	MN	MN
VC395_2770	2914160	s	MN	MN	MN	MN	MN	MN
VC395_A0057	72518	ns	O	O	O	O	O	O
VC395_A0092	106912	ns	O	O	O	O	O	O
VC395_A0111	130633	ns	O	O	O	O	O	O
VC395_A0116	140320	ns	O	-	-	-	-	O
VC395_A0134	155986	ns	O	O	O	O	O	O
VC395_A0170	197133	ns	O	O	O	O	O	O
VC395_A0174	203049	ns	O	O	O	O	O	O
VC395_A0180	205722	ns	O	O	O	O	O	O
VC395_A0319	337020	ns	-	-	-	O	-	O
VC395_A0604	551161	ns	O	O	O	O	O	O
VC395_A0837	798020	ns	O	O	O	-	O	O
VC395_A0879	845782	ns	O	O	O	-	O	O
VC395_A0883	847138	ns	O	O	O	O	O	O
VC395_A0887	853967	ns	-	O	O	O	-	O
VC395_A0923	892474	ns	O	O	O	O	O	O
VC395_A0946	912280	ns	O	O	O	O	O	O
VC395_A0952	920179	ns	O	O	O	-	O	O
VC395_A0956	923611	ns	O	O	O	O	O	O
VC395_A0961	926675	ns	O	O	O	O	O	O
VC395_A0963	928946	ns	O	O	O	O	O	O
VC395_A0967	932023	ns	O	O	O	O	O	O
VC395_A0980	943213	ns	O	O	O	O	O	O
VC395_A0985	947759	ns	O	O	O	O	O	O
VC395_A1042	1006075	ns	O	O	O	O	O	O
VC395_A1054	1019119	ns	O	O	O	-	-	O
VC395_A1064	1031018	ns	O	O	O	O	O	O
VC395_A1111	1084779	ns	O	O	O	O	O	O
VC395_A1132	1107555	ns	O	O	O	O	O	O
VC395_A1133	1108788	ns	O	O	O	O	O	O
VC395_0158	156161	ns	O	O	O	O	O	O

VC395_0149	147641	ns	O	O	O	-	O	O
VC395_0118	113635	ns	O	O	O	O	O	O
VC395_0116	112527	ns	O	O	O	O	O	O
VC395_0100	98682	ns	O	O	O	O	O	O
VC395_0095	93300	ns	O	O	O	O	O	O
VC395_0072	72682	ns	O	O	O	O	O	O
VC395_0069	69426	ns	O	O	O	O	O	O
VC395_0068	69205	ns	O	O	O	O	O	O
VC395_0050	52187	ns	O	O	O	O	O	O
VC395_0036	41361	ns	O	O	O	O	O	O
VC395_0265	271621	ns	O	-	-	-	-	O
VC395_0269	275348	ns	O	-	-	O	-	O
VC395_0337	350905	ns	O	O	O	O	O	O
VC395_0347	362884	ns	O	O	O	O	O	O
VC395_0368	387771	ns	-	O	-	-	O	O
VC395_0371	390441	ns	O	O	O	O	O	O
VC395_0381	407667	ns	O	O	O	O	**3**	O
VC395_0383	409891	ns	O	-	O	-	O	O
VC395_0396	423917	ns	O	O	O	O	O	O
VC395_0437	466613	ns	O	O	O	O	O	O
VC395_0437	466774	ns	O	O	O	O	O	O
VC395_0438	468333	ns	O	O	O	O	O	O
VC395_0443	474817	ns	O	O	O	O	O	O
VC395_0459	492144	ns	O	O	O	O	O	O
VC395_0531	568585	ns	O	O	O	O	O	O
VC395_0534	573373	ns	O	O	O	O	O	O
VC395_0545	582308	ns	O	O	O	O	O	O
VC395_0545	582851	ns	O	O	O	O	O	O
VC395_0551	587922	ns	O	O	O	O	O	O
VC395_0571	610073	ns	O	O	O	-	O	O
VC395_0575	615643	ns	O	O	O	O	O	O
VC395_0585	624507	ns	O	-	O	-	O	O
VC395_0606	639799	ns	O	O	O	O	O	O
VC395_0611	644031	ns	O	O	O	O	O	O
VC395_0656	703550	ns	-	O	O	-	O	O
VC395_0662	710742	ns	O	O	O	-	O	O
VC395_0775	833046	ns	O	O	O	O	O	O

VC395_0786	847162	ns	O	O	O	O	O	O
VC395_0789	850347	ns	O	O	O	O	-	O
VC395_0803	865163	ns	O	O	O	O	O	O
VC395_0900	967079	ns	O	O	O	O	O	O
VC395_0926	992547	ns	O	O	O	O	O	O
VC395_0930	999995	ns	O	O	O	O	O	O
VC395_0936	1005272	ns	-	O	O	O	O	O
VC395_1042	1117780	ns	O	O	O	O	O	O
VC395_1081	1156780	ns	O	O	O	O	O	O
VC395_1086	1161082	ns	O	O	O	O	O	O
VC395_1101	1175190	ns	O	O	O	O	O	O
VC395_1108	1184414	ns	O	O	O	O	O	O
VC395_1109	1185382	ns	O	O	O	O	O	O
VC395_1144	1220002	ns	O	O	O	O	O	O
VC395_1200	1258426	ns	O	O	O	O	O	O
VC395_1221	1282123	ns	O	O	O	O	O	O
VC395_1317	1362738	ns	O	O	O	O	O	O
VC395_1410	1460011	ns	O	O	O	-	O	O
VC395_1435	1489821	ns	O	O	O	O	O	O
VC395_1474	1536195	ns	O	O	O	O	O	O
VC395_1481	1541191	ns	O	O	O	-	O	O
VC395_1483	1543971	ns	O	O	O	O	O	O
VC395_1492	1553535	ns	O	O	O	O	O	O
VC395_1535	1601834	ns	-	O	O	-	-	O
VC395_1535	1603451	ns	-	O	O	-	-	O
VC395_1537	1606032	ns	-	O	-	-	-	O
VC395_1538	1606859	ns	-	O	-	-	-	O
VC395_1566	1634725	ns	O	O	O	O	O	O
VC395_1639	1718815	ns	O	O	O	O	O	O
VC395_1646	1726664	ns	O	O	O	O	O	O
VC395_1650	1730407	ns	O	O	O	O	O	O
VC395_1657	1738352	ns	O	O	O	O	O	O
VC395_1658	1739531	ns	O	O	O	O	O	O
VC395_1675	1755661	ns	O	O	O	O	O	O
VC395_1677	1756812	ns	O	O	O	O	O	O
VC395_1698	1779772	ns	O	O	O	O	O	O
VC395_1701	1782235	ns	-	-	-	O	-	O

VC395_1714	1796397	ns	O	O	O	O	O	O
VC395_1717	1800050	ns	O	O	O	O	O	O
VC395_1769	1865788	ns	O	O	O	O	O	O
VC395_1895	2015389	ns	-	-	O	-	O	O
VC395_1923	2036380	ns	-	-	-	-	O	O
VC395_1934	2044110	ns	O	O	O	O	O	O
VC395_1960	2069748	ns	O	O	O	-	O	O
VC395_1977	2087012	ns	O	O	O	O	O	O
VC395_1983	2094947	ns	O	O	O	O	O	O
VC395_1983	2096068	ns	O	O	O	O	O	O
VC395_1985	2098270	ns	O	O	O	O	O	O
VC395_2017	2136058	ns	O	O	O	O	O	O
VC395_2127	2249969	ns	O	O	O	O	O	O
VC395_2145	2266306	ns	O	O	O	-	O	O
VC395_2145	2269067	ns	O	O	O	-	O	O
VC395_2152	2278840	ns	O	O	O	O	O	O
VC395_2157	2284411	ns	O	O	O	-	O	O
VC395_2184	2309721	ns	O	O	O	O	O	O
VC395_2189	2315837	ns	O	O	O	O	O	O
VC395_2199	2328498	ns	O	O	O	O	O	O
VC395_2239	2363228	ns	O	O	O	O	O	O
VC395_2282	2399987	ns	O	O	O	O	O	O
VC395_2322	2435836	ns	O	O	O	O	O	O
VC395_2347	2469906	ns	O	O	O	O	O	O
VC395_2369	2493341	ns	-	O	O	O	O	O
VC395_2381	2505799	ns	O	O	O	O	O	O
VC395_2403	2527690	ns	O	O	O	O	O	O
VC395_2452	2571754	ns	O	O	O	**3**	O	O
VC395_2459	2585243	ns	O	O	O	O	O	O
VC395_2491	2626733	ns	O	O	O	O	O	O
VC395_2492	2627953	ns	O	O	O	O	O	O
VC395_2597	2746754	ns	O	O	O	O	O	O
VC395_2730	2871717	ns	O	O	O	O	O	O
VC395_2777	2918090	ns	O	O	O	O	O	O
VC395_2801	2941519	ns	O	O	O	O	O	O
VC395_2812	2952548	ns	O	O	O	O	O	O
VC395_2832	2980225	ns	O	O	O	O	O	O

VC395_2847	2993943	ns	O	O	O	O	O	O
VC395_2859	3005993	ns	O	O	O	O	O	O
VC395_A0240	254285	ns	-	-	-	-	-	O/MN
VC395_0277	285312	ns	-	-	-	-	-	O/MN
VC395_0279	288155	ns	-	-	-	-	-	O/MN
VC395_0829	889914	ns	-	-	-	-	-	O/MN
VC395_0837	900169	ns	-	-	-	-	-	O/MN
VC395_0850	918293	ns	-	-	-	-	-	O/MN
VC395_0853	921538	ns	-	-	-	-	-	O/MN
VC395_0853	921672	ns	-	-	-	-	-	O/MN
VC395_0856	924407	ns	-	-	-	-	-	O/MN
VC395_1876	1986957	ns	-	-	-	-	-	O/MN
VC395_1880	1994187	ns	-	-	-	-	-	O/MN
VC395_1885	2003619	ns	-	-	-	-	-	O/MN
VC395_2502	2637281	ns	-	-	-	-	-	O/MN
VC395_1916	2032203	ns	-	-	-	-	-	O/MN
VC395_1914	2029126	ns	-	-	-	-	-	O/MN
VC395_A0059	75382	ns	MN	MN	MN	-	MN	MN
VC395_A0270	290728	ns	MN	MN	MN	MN	MN	MN
VC395_A0286	304811	ns	MN	MN	MN	MN	MN	MN
VC395_A0851	811374	ns	MN	MN	MN	MN	MN	MN
VC395_A1004	967137	ns	MN	MN	MN	-	MN	MN
VC395_A1057	1022967	ns	MN	MN	MN	MN	MN	MN
VC395_A1116	1090894	ns	MN	MN	MN	MN	MN	MN
VC395_0117	113118	ns	MN	MN	MN	MN	MN	MN
VC395_0073	73692	ns	MN	-	-	MN	MN	MN
VC395_0064	66115	ns	MN	MN	MN	MN	MN	MN
VC395_0391	420099	ns	MN	MN	MN	-	MN	MN
VC395_0634	675315	ns	MN	MN	MN	MN	MN	MN
VC395_0735	793894	ns	MN	MN	MN	MN	MN	MN
VC395_0879	950498	ns	MN	MN	MN	MN	MN	MN
VC395_0943	1012672	ns	MN	MN	MN	MN	MN	MN
VC395_1043	1118667	ns	MN	MN	MN	MN	MN	MN
VC395_1109	1185403	ns	MN	MN	MN	MN	MN	MN
VC395_1320	1365536	ns	-	MN	MN	MN	MN	MN
VC395_1433	1488254	ns	MN	MN	MN	MN	MN	MN
VC395_1445	1500097	ns	MN	MN	MN	MN	MN	MN

VC395_1484	1544728	ns	MN	MN	MN	MN	MN	MN
VC395_1637	1716456	ns	MN	MN	MN	MN	MN	MN
VC395_1738	1821750	ns	MN	-	MN	-	-	MN
VC395_1738	1823628	ns	MN	-	-	-	-	MN
VC395_1944	2055929	ns	MN	MN	MN	MN	MN	MN
VC395_1992	2105555	ns	MN	MN	MN	MN	MN	MN
VC395_2063	2182974	ns	MN	MN	MN	MN	MN	MN
VC395_2067	2190127	ns	MN	MN	MN	MN	MN	MN
VC395_2072	2195280	ns	-	MN	MN	MN	MN	MN
VC395_2146	2270953	ns	MN	MN	MN	MN	MN	MN
VC395_2153	2279853	ns	MN	MN	MN	MN	MN	MN
VC395_2184	2310924	ns	MN	MN	MN	MN	MN	MN
VC395_2210	2340985	ns	MN	MN	MN	MN	MN	MN
VC395_2711	2850725	ns	MN	MN	**O**	**-**	MN	MN
VC395_2712	2851464	ns	MN	MN	**O**	MN	MN	MN
VC395_2748	2891778	ns	MN	MN	MN	MN	MN	MN
VC395_0038	41710	nc	O	O	**3**	O	O	O
VC395_0226	234368	nc	O	O	O	O	O	O
VC395_0385	414198	nc	-	O	O	O	O	O
VC395_0622	659274	nc	O	O	O	-	O	O
VC395_0868	940583	nc	O	O	O	O	O	O
VC395_0925	991843	nc	O	O	O	O	O	O
VC395_0927	995878	nc	O	O	O	O	O	O
VC395_0945	1015563	nc	O	O	O	O	O	O
VC395_0946	1018790	nc	O	O	O	O	O	O
VC395_0986	1059761	nc	O	O	O	-	O	O
VC395_0995	1067644	nc	O	O	O	-	O	O
VC395_1088	1162759	nc	O	O	O	O	O	O
VC395_1113	1191428	nc	O	O	O	O	O	O
VC395_1487	1548618	nc	O	O	O	O	O	O
VC395_1612	1684276	nc	O	O	O	O	O	O
VC395_1612	1684710	nc	O	O	O	O	O	O
VC395_1612	1686933	nc	O	O	O	O	O	O
VC395_1674	1754287	nc	O	O	O	O	O	O
VC395_1675	1755935	nc	O	O	O	O	O	O
VC395_1704	1785052	nc	O	O	O	O	O	O
VC395_1766	1860401	nc	O	O	O	O	O	O

VC395_1766	1860415	nc	O	O	O	O	O	O
VC395_1767	1862186	nc	O	O	O	O	O	O
VC395_1977	2087144	nc	O	O	O	O	O	O
VC395_2018	2139359	nc	O	O	O	-	O	O
VC395_2056	2177842	nc	O	O	O	O	O	O
VC395_2340	2464289	nc	O	O	O	O	O	O
VC395_2455	2578201	nc	O	O	O	O	O	O
VC395_2457	2582366	nc	O	O	O	O	O	O
VC395_2505	2643090	nc	O	O	O	O	O	O
VC395_2600	2751902	nc	O	O	O	O	O	O
VC395_2813	2956462	nc	O	O	O	O	O	O
VC395_2813	2957127	nc	O	O	O	O	O	O
VC395_A0021	30448	nc	O	O	O	O	O	O
VC395_A0094	109996	nc	O	O	O	O	O	O
VC395_A0134	157474	nc	O	O	O	O	O	O
VC395_A0296	313877	nc	O	O	O	-	O	O
VC395_A0320	339271	nc	-	-	O	-	-	O
VC395_A0613	564622	nc	O	O	O	O	O	O
VC395_A0605	552216	nc	-	O	O	O	O	O
VC395_A0897	866320	nc	O	O	O	O	O	O
VC395_A0934	904514	nc	O	O	O	-	O	O
VC395_A1029	997314	nc	O	O	O	O	O	O
VC395_A1078	1044314	nc	O	O	O	O	O	O
VC395_A0977	941203	nc	**3**	O	O	**3**	O	O
VC395_0384	411402	nc	-	-	-	-	-	O/MN
VC395_0384	412131	nc	-	-	-	-	-	O/MN
VC395_0862	934115	nc	-	-	-	-	-	O/MN
VC395_1334	1383881	nc	-	-	-	-	-	O/MN
VC395_1599	1660501	nc	-	-	-	-	-	O/MN
VC395_0144	140738	nc	MN	MN	MN	MN	MN	MN
VC395_0107	101887	nc	MN	MN	MN	MN	MN	MN
VC395_0489	524250	nc	MN	MN	MN	-	MN	MN
VC395_0576	616835	nc	MN	MN	MN	MN	MN	MN
VC395_1307	1353651	nc	MN	MN	MN	MN	MN	MN
VC395_1872	1980162	nc	MN	MN	MN	MN	MN	MN
VC395_1943	2055767	nc	MN	MN	MN	MN	MN	MN
VC395_A0021	30291	nc	MN	MN	MN	MN	MN	MN

VC395_A0855	51356	nc	MN	MN	MN	MN	MN	MN

Footnote:
NS, non-synonymous, S, synonymous, NC: non coding.
-, site not in genome
MN and O, Mutation allocated to the MN or O lineage respedctively because base at that site is as found in O395, N16961 respedctively.
3, a third base different to that in MN or O

MZO-3, MZO-2, AM19226, RC385 and 1587' s sequence data from genomes in accession number s AAUU00000000, AAWF00000000, AATY00000000, AAKH00000000, AAUR00000000 respectively. Note that these genomes were all "in progress" and some contigs only, so some segments now absent may get added later.

Red is for base calls not consistent with the consensus or too varaible to allow a consensus.
