# Supplementary material for: A Recalibrated Molecular Clock and Independent Origins for the Cholera Pandemic Clones
Source: PLoS One. 2008 Dec 30;3(12):e4053. doi: 10.1371/journal.pone.0004053 (PMC2605724; doi:10.1371/journal.pone.0004053)
Supplement: Table S3 — Pseudogenes allocated to the M66-2, , N16961, MN and O395 lineages (0.04 MB PDF) [file pone.0004053.s011.pdf]

Table S3. Pseudogenes allocated to the M66-2, , N16961, MN and O395 lineages*

M66-2	N16961	O395	gene name	function
**MNO lineage**				
VC66-2_0242	VC0254	VC395_0286		conserved hypothetical protein
VC66-2_0776	VC0818	VC395_0835		transposase, putative
VC66-2_0803	VC0846	VC395_0862		integrase, degenerate
VC66-2_1698	VC1759	VC395_1874		integrase, truncation
VC66-2_1730	VC1807	VC395_1922		conserved hypothetical protein
VC66-2_2620	VC2700	VC395_2813	pulA	pullulanase
**MN lineage**				
VC66-2_1470	VC1529 (VC1528)	VC395_1647		hypothetical protein
VC66-2_1629	VC1690 (VC1689)	VC395_1808	GalA	Alpha-galactosidase
VC66-2_A0847	VCA0887	VC395_A0912		Putative intracellular protease/amidase
**M lineage**				
VC66-2_0182	VC0194	VC395_0226	ggt	gamma-glutamyltranspeptidase
VC66-2_0200	VC0212	VC395_0244	msbB	lipid A biosynthesis (kdo)2-(lauroyl)-lipid IVA acyltransferase
VC66-2_0227	VC0239	VC395_0271		conserved hypothetical protein
VC66-2_0559	VC0601	VC395_0618	hrpB	ATP-dependent helicase HrpB
VC66-2_0658	VC0700	VC395_0717	slt	soluble lytic murein transglycosylase
VC66-2_1041	VC1085	VC395_1100		sensor histidine kinase
VC66-2_1242	VC1287	VC395_1406	mdoH	periplasmic glucans biosynthesis protein MdoH
VC66-2_1888	VC1964	VC395_2079		hypothetical protein
VC66-2_1889	VC1965	VC395_2080		hypothetical protein
VC66-2_1994	VC2070	VC395_2185	sixA	phosphohistidine phosphatase
VC66-2_2309*	VC2386	VC395_2501*		conserved hypothetical protein
VC66-2_A0071	VCA0074	VC395_A0067		GGDEF family protein
VC66-2_A0274(VC66-2_A0271)	VCA0275	VC395_A0312		IS1004
**N lineage**				
VC66-2_0243	VC0258 (VC0255)	VC395_0287		rfbT-related protein
VC66-2_0269	VC0284	VC395_0313		TonB system receptor
VC66-2_0541	VC0583	VC395_0600	hapR	hemagglutinin/protease regulatory protein
VC66-2_0779	VC0822 (VC0821)*	VC395_0838*		hypothetical protein
VC66-2_1560	VC1620	VC395_1738		RTX toxin
VC66-2_A0131	VCA0133	VC395_A0126	pnuC	pnuC protein
VC66-2_A0939	VCA0979 (VCA0980)	VC395_A1004		methyl-accepting chemotaxis protein
**O lineage**				
VC66-2_0293	VC0308	VC395_0352		hypothetical protein

VC66-2_0397	VC0412	VC395_0456		hypothetical protein
VC66-2_0779	VC0822 (VC0821)*	VC395_0838*		hypothetical protein
VC66-2_0878	VC0921	VC395_0937		polysaccharide export protein, putative
VC66-2_1029	VC1073	VC395_1088		chitinase, putative
VC66-2_1061	VC1105	VC395_1120		conserved hypothetical protein
VC66-2_1123	VC1168	VC395_1234(VC395_1287)	gltP-1	proton/glutamate symporter
VC66-2_1329	VC1374	VC395_1493		DnaK-related protein
VC66-2_1330	VC1375	VC395_1494		hypothetical protein
VC66-2_1404	VC1448	VC395_1568	rtxB	RTX toxin transporter
VC66-2_1407	VC1451	VC395_1569	rtxA	RTX toxin RtxA
VC66-2_1435	VC1492	VC395_1612		conserved hypothetical protein
VC66-2_1539	VC1599	VC395_1715		GGDEF family protein
VC66-2_1554	VC1614	VC395_1730(VC395_1732)		conserved hypothetical protein
VC66-2_1589	VC1650	VC395_1767	vcc	collagenase
VC66-2_1653	VC1713	VC395_1830		transcriptional regulator, AsnC family
VC66-2_1763	VC1840	VC395_1955		conserved hypothetical protein
VC66-2_2309*	VC2386	VC395_2501		conserved hypothetical protein
VC66-2_A0105	VCA0107	VC395_A0100		conserved hypothetical protein
VC66-2_A0112	VCA0114	VC395_A0107		conserved hypothetical protein
VC66-2_A0118	VCA0120	VC395_A0113		IcmF-related protein
VC66-2_A0144	VCA0146	VC395_A0139		Putative TRAP transporter large permease protein
VC66-2_A0193	VCA0197	VC395_A0236	guaC	GMP reductase
VC66-2_A0215	VCA0219	VC395_A0256	hlyA	haemolysin
VC66-2_A0255	VCA0258	VC395_A0296		conserved hypothetical protein
VC66-2_A0483	VCA0524	VC395_A0800		conserved hypothetical protein
VC66-2_A0807	VCA0848	VC395_A0872		GGDEF family protein
VC66-2_A0855	VCA0895	VC395_A0920		chemotactic transducer-related protein
VC66-2_A0922	VCA0962	VC395_A0987		conserved hypothetical protein
VC66-2_A1059	VCA1102	VC395_A1123		conserved hypothetical protein

Note:

Pseudogenes in the major integron are not included in this table

VC66-2_2309* and its equivalent in O395 are independently inactivated in M66-2 and O395

VC0822 (VC0821)* and its equivalent in O395 are independently inactivated in N16961 and O395
