# Supplementary material for: A Recalibrated Molecular Clock and Independent Origins for the Cholera Pandemic Clones
Source: PLoS One. 2008 Dec 30;3(12):e4053. doi: 10.1371/journal.pone.0004053 (PMC2605724; doi:10.1371/journal.pone.0004053)
Supplement: Table S4 — Integron cassette groups (0.08 MB PDF) [file pone.0004053.s012.pdf]

Table S4. **Integron cassette groups**

Group	Cassette form	locations number	Designation in three genomes		
			M66-2	N16961	O395
1	1	4		6	
	"		79	39	
	"			54	30
	"				48
	2	2	65		
	"		90	85	11
	3	1	103	141	106
	4	1	110	148	113
	5	2	15		
	"		124	162	123
	6	2	56	25	
	"		135	175	138
	7	2	3		
	"			79	5
	8	2	10		
	"			124	89
	9	1	42	11	
	10	1	44	13	
	11	2	45	14	
	"				69
	12	1	74	34	
	13		40		
	14			75	
	15	1		59	35
	16	1		87	13
	17	1		126	91
	18	1		97	23
	19	1		100	62
	20	2			28
	"			104	
	21	2			25
	"			111	
	22			72	
	23		20		
	24		18		
	25		7		
	26		61		
2	27	2	4		
	"		82		51

	28			41	
	29			107	
3	30	2	6		
	"		14		
	31				29
4	32	2	9		
	"		92	130	95
	33		119	157	118
5	34	2	11		
	"			125	90
	35	1	138	178	141
6	36	2	12		
	"		54	23	
	37	1	84		52
	38	1	93	131	96
	39	1		99	61
	40			52	
7	41		19		
	42			78	
8	43	1	87		55
	44		22		
9	45		28		
	46	2	30		
	"		73	33	
	47	2	31		
	"		60		
	48	1	67	28	
	49	1	68	29	
	50	1	108	146	111
	51			42	
	52			70	
	53	1		83	9
	54	1		84	10
	55	2			27
	"			108	
	56	1		116	81
	57	1		120	85
	58				77
10	59	2	32		
	"		62		
	60	1		58	34
	61	2			4
	"			63	39

	62	2		91	17
	"				74
11	63	1	52	21	
	64	1		118	83
	65	1	114	152	
	66	1	115	153	114
12	67	2	59		
	"		130	168	130
	68				75
13	69	1	64		
	70	1		96	22
14	71	3	69	30	
	"			44	
	"				58
	72	1	111	149	
15	73	2	72	32	
	"		112	150	
	74	1			132
16	75	1	80		49
	76	1	131	171	134
	77			76	
	78				72
17	79	1	97	135	100
	80	1		117	82
	81	2			26
	"			106	
	82	1		60	36
18	83	1	107	145	110
	84			101	
19	85	2		94	20
	"		121	159	120
	86	2		50	
	"		139	179	142
20	87	1		90	16
	88	1		170	133
21	89	1		113	
	90	1		121	86
22	91	1		115	
	92	1			66
	93	2	1		
	"		86		54
	94	3	2		
	"			71	

"			73	
95	2	5		
"		75	35	
96	2	13		
"			74	
97	3	16		
"			51	
"		137	177	140
98	2	17		
"			61	37
99	2	25		
"		134	174	137
100	2	29		
"		129	167	129
101	2	33		
"			122	87
102	2	36		
"			77	
103	1	41	10	
104	2	43	12	
"		113	151	
105	1	46	15	
106	1	47	16	
107	1	48	17	
108	1	49	18	
109	2	50	19	
"			80	6
110	1	51	20	
111	2	53	22	
"			109	
112	3	55	24	
"				24
"		125	163	125
113	1	57	26	
114	1	58	27	
115	4	63		
"			40	
"				47
"			127	92
116	2	66		
"		91	86	12
117	4	70	31	
"			7	

"			46	
"				60
118	1	76	36	
119	1	77	37	
120	1	78	38	
121	1	81		50
122	1	85		53
123	1	88		56
124	1	89	69	57
125	1	94	132	97
126	1	95	133	98
127	3	96	134	99
"			119	84
"			47	
128	1	98	136	101
129	1	99	137	102
130	1	100	138	103
131	1	101	139	104
132	1	102	140	105
133	1	104	142	107
134	1	105	143	108
135	1	106	144	109
136	1	109	147	112
137	1	116	154	115
138	1	117	155	116
139	1	118	156	117
140	1	120	158	119
141	1	122	160	121
142	2		93	19
"		123	161	122
143	1	126	164	126
144	1	127	165	127
145	1	128	166	128
146	1	132	172	135
147	1	133	173	136
148	1	136	176	139
149	3		1	
"			65	41
"				43
150	5		2	
"				1
"				2
"			66	42

"				44
151	2		45	
"				59
152	1		55	31
153	1		56	32
154	1		57	33
155	1		62	38
156	2			3
"			64	40
157	1		67	45
158	1		68	46
159	1		81	7
160	1		82	8
161	1		88	14
162	1		89	15
163	1		92	18
164	1		95	21
165	1		102	63
166	1		103	64
167	1		114	65
168	1		123	88
189	1		128	93
170	1		129	94
171	1		169	131
172	1	8		
173	1	21		
174	1	23		
175	1	24		
176	1	26		
177	1	27		
178	1	34		
179	1	35		
180	1	37		
181	1	38		
182	1	39		
183	1	71		
184	1	83		
185	1		3	
186	1		4	
187	1		5	
188	1		8	
189	1		9	
190	1		43	

191	1	48	
192	1	49	
193	1	53	
194	1	98	
195	1	105	
196	1	110	
197	1	112	
198	1		67
199	1		68
200	1		70
201	1		71
202	1		73
203	1		76
204	1		78
205	1		79
206	1		80
207	1		124

Note: Cassette forms 1-92 that have been allocated to groups are indicated by a pink circle in Figure S4. Cassette forms allocated to the same group are homologous based on BLASTN searches with an e value less than 10^{-10} . Cassettes placed on the same row are in the same location as shown in Figure S4.
