# Supplementary material for: A Recalibrated Molecular Clock and Independent Origins for the Cholera Pandemic Clones
Source: PLoS One. 2008 Dec 30;3(12):e4053. doi: 10.1371/journal.pone.0004053 (PMC2605724; doi:10.1371/journal.pone.0004053)
Supplement: Table S5 — Major indels in the 3 genomes (0.04 MB PDF) [file pone.0004053.s013.pdf]

Table S5. Major indels in the 3 genomes

Indels	Location[*]	Size(bp)	Lineage[†]	Main properties	Additional properties[‡]
**phage**					
B6	1220297..1254162	33866	O, O/NM	Mu-like prophage	B6 and B7 are 100% identical and more homologous to the *Shewanella* Mu-like prophage than the original Mu phage.
B7	1297410..1331275	33866	O, O/NM	Mu-like prophage	
S1	214245..249127	34883	O, O/NM	K139-like phage	251 bp DRs.
**ctx related**					
B9	1564133..1573773	9641	NO, delM	ctx phage	18 bp imperfect DRs.
B10	1564133..1566857	2725	N, O/NM	RS1 in N	
B11	1652612..1655299	2688	O, O/NM	RS2 in O	
S3	696316..703355	7040	O, insO	ctx phage	
**island with integrase**					
B1	5823..31424	25602	O, O/NM	Novel O island	has recombinase/integrase as last gene;2 Transposase (VC395_0018, VC395_0019) and 1 DNA-invertase; low GC, no DRs.
B2	175198..189466	14269	N, insN	VSP-I	1 putative integrase(VC0185); low GC and no DRs. Within a region that has undergone recombination.
B4	332373..349315	16943	O, O/NM	Novel O island	1 VINT_BPPH8 (P06155) Integrase;16 bp DRs, and regions of low GC.
B5	522863..550186	27324	N, insN	VSP-II	an integrase gene as last gene and tRNA gene downstream; low GC in parts, imperfect DRs.
**Insertion sequence (IS) and IS associated**					
S4	686227..687260	1034	O, O/NM	ISVch1	VC395_A0738, IS481 family; 7 bp DRs. At the end of a region that has undergone recombination.
B14	1813134..1813755	622	O, O/NM	IS1004	VC395_1731, 8 bp imperfect DRs.
B17	1956298..1956919	622	M, insM	IS1004	VC66-2_1798, 8 bp DRs.

	B18	2417061..2417682	622	O, O/NM	IS1004	VC395_2301, 9 bp DRs.
	B19	2835084..2835705	622	O, O/NM	IS1004	IS1004 (VC395_2681), 8 bp imperfect DRs.
	B12	1583125..1584394	1270	MN, O/MN	ISVch4	orfAB (VC66-2_1420/VC66-2_1421) in TLC element.
	B3	262291..263551	1261	N, insN	ISVch4	orfAB (VC0256/VC0257), 6 bp DRs.
	S2	292398..293659	1261	M, insM	ISVch4	orfAB (VC66-2_A0272/VC66-2_A0273),.no DRs.
	S6	637261..639633	2373	O, O/NM	ISVch5	VC395_A0686 (ISVch5) and 3 hypothetical proteins; same site as S5; No DRs. Within recombinant region.
	B16	1938769..1949510	10742	NO, delM	Partial VPI-2	VPI-2 includes 2 Mu related genes; low GC, no DRs.
	S9	465338..469823	4486	O, O/NM	ISVch4+5 genes	Starts with ISVch4 with 8 bp DRs., and adjacent to the ISVch4 at end of big inversion. Effectively 2 ISs with 5 hypothetical proteins between.
**rrn**						
	B20	2927885..2933644	5760	NO, delM	*rrn* operon	2 16S-23S *rrn* segments in N16961 and O395, but only one in M66-2.
**others**						
	B8	1547914..1555781	7868	MN, delO	Partial RTX region deletion	Part of *rtxB* gene, *rtxC*, VC66-2_1405 equivalent and part of *rtxA* gene are absence in O395.
	B13	1665519..1670354	4836	O, O/NM	TLC element	3rd copy of toxin-linked cryptic (TLC) element in CTX region.
	B15	1906519..1907323	805	O, O/NM	Hypothetical ORF	1 hypothetical protein (VC395_1807). Low GC, no DRs. Within a region that has undergone recombination.
	S5	563579..566177	2599	MN,O/NM	a hypothetical protein	Present in MN; located at same site as S6.
	S7	672301..677746	5446	MN, O/NM	3 unknown ORF	Present in MN; located at same site as S8; Within a region that has undergone recombination.
	S8	527797..530766	2970	O, O/NM	1 ATPase gene	Present in O; same site as S7.

[*] For deletions the location given is N16961 genome positions for M66-2 and O395 deletions, and O395 genome positions for deletions in N16961.

[†] Symbol before comma indicates the strain where the indel is present: O, O395; N, N16961; M, M66-2.
Abbreviation after comma indicates lineage to which the insertion (ins) or deletion (del) is attributed.

[‡] DR, direct repeat;
