# Supplementary material for: A Recalibrated Molecular Clock and Independent Origins for the Cholera Pandemic Clones
Source: PLoS One. 2008 Dec 30;3(12):e4053. doi: 10.1371/journal.pone.0004053 (PMC2605724; doi:10.1371/journal.pone.0004053)
Supplement: Table S6 — Substitution rates and divergence times (0.04 MB PDF) [file pone.0004053.s014.pdf]

Table S6. Substitution rates and divergence times

Chromosome	No. Genes analysed[*]	Total size (bp)	Pairs[†]	K_a[‡]	K_s[‡]	Time (years)[§]	
						Whittam rate	Dykhuizen rate
Small	435	370,734	M-N	6.5×10^{-5}	3.9×10^{-5}	6,500	1,300
	435	371,706	M-O	1.7×10^{-4}	2.17×10^{-4}	36,167	7,233
	435	371,703	N-O	1.58×10^{-4}	2.25×10^{-4}	37,500	7,500
Large	1,644	1,503,666	M-N	6.4×10^{-5}	7.4×10^{-5}	12,333	2,467
	1,644	1,503,399	M-O	1.87×10^{-4}	2.76×10^{-4}	46,000	9,200
	1,644	1,503,693	N-O	1.95×10^{-4}	3.19×10^{-4}	53,167	10,633
Both	2,079	1,874,400	M-N	6.4×10^{-5}	6.7×10^{-5}	11,167	2,233
	2,079	1,875,105	M-O	1.83×10^{-4}	2.65×10^{-4}	44,167	8,833
	2,079	1,875,396	N-O	1.88×10^{-4}	3.01×10^{-4}	50,167	10,033

[*]Number of genes remaining after excluding genes in recombination regions.
[†]M refers to M66-2, O to O395, and N to N16961.
[‡]K_a and K_s: non-synonymous and synonymous substitution rate.
[§]Whittam and Dykhuizen rates refer to mutation rates used for calculation of divergence time based on K_s which is $6x10^{-9}$ per site per year and $3x10^{-8}$ per site per year respectively [1,2].

1. Guttman DS, Dykhuizen DE (1994) Clonal divergence in *Escherichia coli* as a result of recombination, not mutation. Science 266: 1380-1383.
2. Whittam TS (1996) Genetic variation and evolutionary processes in natural populations of *Escherichia coli*. In: Neidhardt FC, Curtiss R, Ingraham JL, Lin ECC, Low KB et al., editors. *Escherichia* and *Salmonella*: Cellular and Molecular Biology. 2nd ed. Washington, D. C.: ASM Press. pp. 2708-2720.
