# Supplementary material for: A Recalibrated Molecular Clock and Independent Origins for the Cholera Pandemic Clones
Source: PLoS One. 2008 Dec 30;3(12):e4053. doi: 10.1371/journal.pone.0004053 (PMC2605724; doi:10.1371/journal.pone.0004053)
Supplement: Table S7 — Genes in recombinant segments with protein product having ks/ka less than 4 (0.10 MB PDF) [file pone.0004053.s015.pdf]

Table S7. Genes in recombinant segments with protein product having ks/ka less than 4

O395	gene name	Function	rec*	ks/ka	expression data†
VC395_0130		DNA topoisomerase I-related protein	M	0.70	0.32
VC395_0129	purK	phosphoribosylaminoimidazole carboxylase, ATPase subunit	M	3.64	0.19
VC395_0127		conserved hypothetical protein	M	0.00	0.42
VC395_0212		conserved hypothetical protein	O	3.54	1.93
VC395_0221		conserved hypothetical protein	M	0.00	0.34
VC395_0223		Threonine efflux protein	M	3.89	2.17
VC395_0237		conserved hypothetical protein	MO	3.71	0.68
VC395_0257		putative lipopolysaccharide biosynthesis protein	O	3.41	0.78
VC395_0262		conserved hypothetical protein	O	0.00	1.25
VC395_0293		conserved hypothetical protein	O	0.00	1.65
VC395_0303		conserved hypothetical protein	O	3.80	3.63
VC395_0308		conserved hypothetical protein	O	0.63	2.82
VC395_0360		conserved hypothetical protein	O	0.00	1.24
VC395_0418		conserved hypothetical protein	O	2.14	0.54
VC395_0424		conserved hypothetical protein	O	0.00	#N/A
VC395_0425		conserved hypothetical protein	O	2.16	1.18
VC395_0563		conserved hypothetical protein	O	0.00	0.75
VC395_0614	sfsA	sugar fermentation stimulation protein	O	2.19	0.43
VC395_0615		conserved hypothetical protein	O	0.72	0.98
VC395_0617		conserved hypothetical protein	O	2.84	1.03
VC395_0650	ompU	outer membrane protein OmpU	O	0.00	0.47
VC395_0678		conserved hypothetical protein	O	3.59	0.73

VC395_0687		conserved hypothetical protein	O	0.00	1.81
VC395_0698	ribF	riboflavin kinase/FMN adenylyltransferase	O	3.51	0.43
VC395_0703		conserved hypothetical protein	O	2.06	1.32
VC395_0718		trp operon repressor, putative	O	3.39	0.51
VC395_0724		conserved hypothetical protein	O	0.00	1.08
VC395_0750		conserved hypothetical protein	O	1.64	1.13
VC395_0754		putative acetoin utilization protein	O	1.84	0.86
VC395_0755		conserved hypothetical protein	O	0.00	0.86
VC395_0757		conserved hypothetical protein	O	0.00	0.81
VC395_0771		conserved hypothetical protein	MO	2.80	1.04
VC395_0774		conserved hypothetical protein	MO	2.06	0.35
VC395_0782		conserved hypothetical protein	O	0.00	0.87
VC395_0783	xseA	exodeoxyribonuclease VII, large subunit	O	0.00	0.56
VC395_0814	citD	citrate lyase, gamma subunit	O	2.90	1.01
VC395_0817	citX	citX protein	O	0.00	0.78
VC395_0837	tagA	ToxR-activated gene A protein	O	1.74	0.47
VC395_0840	tagD	tagD protein	O	3.95	0.88
VC395_0841	tcpI	toxin co-regulated pilus biosynthesis protein I	O	3.72	0.23
VC395_0842	tcpP	toxin co-regulated pilus biosynthesis protein P	O	1.41	0.78
VC395_0906	ispA	geranyltranstransferase	N	0.00	0.47
VC395_0919	syd	syd protein	O	0.72	0.58
VC395_0953		conserved hypothetical protein	N	0.58	1.17
VC395_0959	lipB	lipoate-protein ligase B	N	1.23	0.63
VC395_0970		conserved hypothetical protein	O	1.20	1.14
VC395_0979	crr	PTS system, glucose-specific IIA component	N	0.00	0.52

VC395_0982		conserved hypothetical protein	N	0.00	0.64
VC395_1002	hemH	ferrochelatase	O	3.10	0.56
VC395_1012	glnS	glutaminyl-tRNA synthetase	O	0.00	0.29
VC395_1022		conserved hypothetical protein	O	2.17	0.95
VC395_1102		response regulator	O	2.46	0.51
VC395_1103		sensor histidine kinase	O	0.00	0.38
VC395_1121		preprotein translocase SecA subunit-related protein	O	4.00	0.95
VC395_1297		conserved hypothetical protein	O	0.00	1.15
VC395_1298		pseudouridine synthase family 1 protein	O	0.00	0.78
VC395_1303		NifS-related protein	O	2.77	1.06
VC395_1340		conserved hypothetical protein	O	0.00	2.14
VC395_1342		conserved hypothetical protein	O	0.00	2.30
VC395_1344		conserved hypothetical protein	O	0.00	1.89
VC395_1346		conserved hypothetical protein	O	1.05	1.81
VC395_1349		conserved hypothetical protein	O	0.00	2.13
VC395_1364		putative vitamin B12 ABC transporter, ATP-binding protein Btı	O	1.34	0.84
VC395_1399		conserved hypothetical protein	O	3.86	0.61
VC395_1415	thiD	phosphomethylpyrimidine kinase	O	3.80	1.33
VC395_1442		conserved hypothetical protein	O	0.00	1.37
VC395_1449		conserved hypothetical protein	O	1.87	0.67
VC395_1450		conserved hypothetical protein	O	2.14	1.63
VC395_1497		conserved hypothetical protein	O	1.80	1.35
VC395_1520	cheB-1	protein-glutamate methylesterase CheB	O	3.70	0.59
VC395_1525		methyl-accepting chemotaxis protein	O	2.32	0.61
VC395_1539		conserved hypothetical protein	MO	3.84	0.75

VC395_1548	potA	spermidine/putrescine ABC transporter, ATP-binding protein	MO	1.93	0.56
VC395_1560	ccoQ	cytochrome c oxidase, subunit CcoQ	O	0.00	1.45
VC395_1565		sensor histidine kinase/response regulator	O	1.76	0.36
VC395_1615		conserved hypothetical protein	MN	3.79	0.95
VC395_1676		conserved hypothetical protein	M	0.00	1.70
VC395_1677	perA	catalase/peroxidase	M	2.07	0.29
VC395_1679		beta-lactamase-related protein	O	0.00	0.35
VC395_1685		conserved hypothetical protein	O	0.00	1.05
VC395_1688		conserved hypothetical protein	O	0.51	0.63
VC395_1691		conserved hypothetical protein	O	3.22	0.58
VC395_1692		conserved hypothetical protein	O	0.66	1.13
VC395_1695		enterobactin synthetase component F-related protein	O	0.00	0.30
VC395_1700	ankB	ankB protein	N	3.82	0.66
VC395_1703		conserved hypothetical protein	N	0.35	0.71
VC395_1731		conserved hypothetical protein	O	0.00	0.32
VC395_1745		conserved hypothetical protein	O	3.86	0.30
VC395_1803		conserved hypothetical protein	O	3.12	0.41
VC395_1814		DNA-binding protein inhibitor Id-2-related protein	O	3.33	0.79
VC395_1815		conserved hypothetical protein	O	1.04	1.21
VC395_1818	yciB	intracellular sepatation protein A	O	3.90	0.81
VC395_1839		conserved hypothetical protein	O	1.88	0.52
VC395_1845		conserved hypothetical protein	N	0.00	1.34
VC395_1847		conserved hypothetical protein	NO	3.98	1.12
VC395_1849		conserved hypothetical protein	O	2.56	0.97
VC395_1850		conserved hypothetical protein	O	0.00	0.84

VC395_1851	aat	leucyl/phenylalanyl-tRNA-protein transferase	O	2.13	0.43
VC395_1852		arginyl-tRNA-protein transferase-related protein	O	1.90	0.39
VC395_2020	ald	alanine dehydrogenase	O	0.00	0.62
VC395_2021		conserved hypothetical protein	O	1.84	0.57
VC395_2026	pyrF	orotidine 5`-phosphate decarboxylase	O	0.00	0.86
VC395_2046		conserved hypothetical protein	O	0.00	0.47
VC395_2060		FAD monooxygenase, PheA/TfdB family	O	1.68	0.59
VC395_2094	dgt	deoxyguanosinetriphosphate triphosphohydrolase	O	0.00	0.64
VC395_2098		putative peptidase	O	2.46	0.54
VC395_2101		putative serine hydrolase	O	1.11	1.01
VC395_2106		conserved hypothetical protein	O	0.00	1.58
VC395_2116		conserved hypothetical protein	O	1.84	0.56
VC395_2117		conserved hypothetical protein	O	0.93	0.62
VC395_2224		conserved hypothetical protein	O	3.67	0.56
VC395_2227		conserved hypothetical protein	O	3.73	1.20
VC395_2244	fliI	flagellum-specific ATP synthase FliI	O	1.82	0.81
VC395_2267		D,D-carboxypeptidase-related protein	O	2.54	0.73
VC395_2268		conserved hypothetical protein	O	0.00	1.15
VC395_2269		conserved hypothetical protein	O	0.00	1.29
VC395_2287		conserved hypothetical protein	O	0.00	1.27
VC395_2292	hemK	hemK protein	O	0.40	1.84
VC395_2317	cheR-2	chemotaxis protein methyltransferase CheR	O	2.34	1.30
VC395_2431		conserved hypothetical protein	O	0.00	1.08
VC395_2459		conserved hypothetical protein	N	0.00	0.30
VC395_2480		conserved hypothetical protein	O	3.89	1.00

VC395_2482		conserved hypothetical protein	O	0.00	0.93
VC395_2544		conserved hypothetical protein	O	2.36	1.14
VC395_2549		conserved hypothetical protein	O	1.41	1.68
VC395_2565	mazG	mazG protein	O	3.11	0.51
VC395_2572	acpS	holo-(acyl-carrier-protein) synthase	O	2.82	1.33
VC395_2585		conserved hypothetical protein	O	0.66	1.25
VC395_2623		conserved hypothetical protein	O	1.11	2.44
VC395_2635		conserved hypothetical protein	O	0.00	2.72
VC395_2638		3-deoxy-D-manno-octulosonate 8-phosphate phosphatase	O	1.83	0.88
VC395_2653		conserved hypothetical protein	O	0.00	1.35
VC395_2656		conserved hypothetical protein	O	0.00	1.43
VC395_2670	cysC	adenylylsulfate kinase	O	3.77	1.16
VC395_2680	fklB	peptidyl-prolyl cis-trans isomerase, FKBP-type	O	2.96	0.92
VC395_2689	secY	preprotein translocase, SecY subunit	O	1.86	0.62
VC395_2690	rplO	ribosomal protein L15	O	0.00	1.67
VC395_2717	slyD	peptidyl-prolyl cis-trans isomerase, FKBP-type	O	3.85	0.78
VC395_2725		conserved hypothetical protein	O	3.18	1.46
VC395_2810		GGDEF family protein	O	0.73	0.29
VC395_2812	dcuA	C4-dicarboxylate transporter, anaerobic	M	1.06	0.29
VC395_0187	atpF	ATP synthase F0, B subunit	M	2.54	0.29
VC395_0181	gidB	glucose inhibited division protein B	O	0.00	0.30
VC395_A0015	vgrG-2	vgrG protein	O	3.83	0.20
VC395_A0026		conserved hypothetical protein	O	3.30	1.60
VC395_A0048		conserved hypothetical protein	O	0.00	0.29
VC395_A0055		conserved hypothetical protein	O	0.00	0.83

VC395_A0068		conserved hypothetical protein	O	1.40	0.25
VC395_A0069		conserved hypothetical protein	O	0.00	#N/A
VC395_A0078		conserved hypothetical protein	O	0.00	0.11
VC395_A0102		conserved hypothetical protein	O	0.00	0.23
VC395_A0131		conserved hypothetical protein	O	0.00	0.56
VC395_A0139		putative TRAP transporter large permease protein	O	0.00	0.16
VC395_A0151		conserved hypothetical protein	NO	0.00	0.38
VC395_A0155		conserved hypothetical protein	MNO	1.18	0.37
VC395_A0165		conserved hypothetical protein	N	0.78	0.26
VC395_A0169	frnE	frnE protein	N	3.90	0.20
VC395_A0806	fruB	PTS system, fructose-specific IIA/FPR component	O	3.66	0.28
VC395_A0801		aminotransferase, class II	O	2.06	0.23
VC395_A0799		conserved hypothetical protein	O	2.35	1.05
VC395_A0791	tatA-2	tatA protein	O	0.46	0.51
VC395_A0790		DedA family protein	O	0.49	0.48
VC395_A0789		conserved hypothetical protein	O	3.56	0.39
VC395_A0786		cytochrome b561, putative	O	1.99	0.66
VC395_A0778		conserved hypothetical protein	O	2.52	0.28
VC395_A0743	ddlA	D-alanine--D-alanine ligase	O	0.00	0.31
VC395_A0733		conserved hypothetical protein	O	2.07	0.29
VC395_A0732		conserved hypothetical protein	O	0.00	0.37
VC395_A0689		conserved hypothetical protein	O	0.00	0.77
VC395_A0671		conserved hypothetical protein	O	3.17	0.48
VC395_A0669		conserved hypothetical protein	O	0.00	0.31
VC395_A0661		conserved hypothetical protein	O	3.26	0.64

VC395_A0657	cscA	sucrose-6-phosphate dehydrogenase	O	3.61	0.27
VC395_A0656	cscK	fructokinase	O	3.16	0.58
VC395_A0650		CbbY family protein	O	0.00	0.38
VC395_A0633	napB	periplasmic nitrate reductase, cytochrome c-type protein	O	3.85	0.82
VC395_A0616		conserved hypothetical protein	O	0.98	0.43
VC395_A0599		conserved hypothetical protein	N	0.00	0.24
VC395_A0581		conserved hypothetical protein	O	0.00	0.71
VC395_A0571		conserved hypothetical protein	NO	0.00	0.44
VC395_A0567		conserved hypothetical protein	NO	0.00	0.69
VC395_A0558		transcriptional regulator, LysR family	M	3.35	0.38
VC395_A0553		conserved hypothetical protein	M	1.37	0.41
VC395_A0545		conserved hypothetical protein	MO	0.93	0.51
VC395_A0544		conserved hypothetical protein	O	0.00	0.61
VC395_A0536		conserved hypothetical protein	O	3.02	0.35
VC395_A0534		conserved hypothetical protein	O	4.00	0.20
VC395_A0834		conserved hypothetical protein	MO	0.73	0.85
VC395_A0858		conserved hypothetical protein	N	1.16	0.70
VC395_A0866		conserved hypothetical protein	O	0.00	0.96
VC395_A0868		conserved hypothetical protein	O	0.00	1.33
VC395_A0890		conserved hypothetical protein	O	2.09	0.61
VC395_A0891	ompW	outer membrane protein OmpW	O	1.74	0.28
VC395_A0918		conserved hypothetical protein	N	0.00	0.55
VC395_A0933	hutX	hutX protein	N	0.00	0.42
VC395_A0939	hutC	putative hemin ABC transporter, permease protein	N	0.00	0.33
VC395_A0941		conserved hypothetical protein	N	1.99	0.56

VC395_A0944		conserved hypothetical protein	N	0.00	0.59
VC395_A0957		conserved hypothetical protein	N	2.18	0.44
VC395_A0960		conserved hypothetical protein	N	0.00	0.63
VC395_A0961		conserved hypothetical protein	N	2.77	0.22
VC395_A0964		sensory box/GGDEF family protein	N	2.13	0.31
VC395_A0989		putative glycine cleavage operon activator	M	2.75	0.53
VC395_A0993		conserved hypothetical protein	M	0.00	0.96
VC395_A0998		conserved hypothetical protein	M	0.37	0.86
VC395_A1001		conserved hypothetical protein	O	0.00	0.57
VC395_A1016	grx2	glutaredoxin 2	O	1.73	0.54
VC395_A1019		conserved hypothetical protein	O	0.47	1.33
VC395_A1101	pdxH	pyridoxamine 5`-phosphate oxidase	O	0.00	0.61

Footnote:

* This column indicates the lineage in which the recombination occurred.
† The expression data was from Bina et al.[1] and the data is the ratio of cDNA of the stool samples and genomic DNA. #N/A in this column means that no expression data can be obtained.

1. Bina J, Zhu J, Dziejman M, Faruque S, Calderwood S, et al. (2003) ToxR regulon of Vibrio cholerae and its expression in vibrios shed by cholera patients. Proc Natl Acad Sci U S A 100: 2801-2806.
