# Supplementary material for: A Recalibrated Molecular Clock and Independent Origins for the Cholera Pandemic Clones
Source: PLoS One. 2008 Dec 30;3(12):e4053. doi: 10.1371/journal.pone.0004053 (PMC2605724; doi:10.1371/journal.pone.0004053)
Supplement: Table S8 — Corrections to N16961 genome bases (0.06 MB PDF) [file pone.0004053.s016.pdf]

Table S8. Corrections to N16961 genome sequence

chromosome	location	gene location	original base	corrected base
Large	424629	*VC0398*	G	T
Large	424645	*VC0398*	A	GA
Large	424670	*VC0398*	G	none
Large	638169	*VC0607*	C	TC
Large	998435	*VC0933 (VC0932)*	T	CT
Large	1228191	*VC1157 (VC1156)*	C	CC
Large	1335598	*VC1261*	A	G
Large	1618601	*VC1504 (VC1505)*	C	CC
Large	1625933	*VC1513*	C	CC
Large	1649049	*VC1536 (VC1535)*	C	none
Large	1676600	*VC1563(VC1564)*	C	CC
Large	1676703	*VC1563(VC1564)*	G	CGA
Large	1748765	*VC1625*	T	CT
Large	1875074	*VC1739 (VC1738)*	T	TT
Large	1900854	*VC1761 (VC1760)*	T	none
Large	2489046	*VC2338*	G	GG
Large	2262543	*VC2105 (VC2104)*	T	none
Large	2266655	*VC2109*	C	CC
Large	2318560	*VC2173*	G	none
Large	2657595	*VC2477 (VC2476)*	T	CT
Large	2738434	*VC2556 (VC2557)*	T	CT
Large	2907037	*VC2733*	G	GG
Large	335660	*VC0321*	G	C*
Large	553569	*VC0518*	C	CC*
Large	555404	*VC0521*	G	GG*
Large	663766	*VC0624*	T	A*
Large	849965	*VC0793m*	G	GG*
Large	1087962	*VC1018*	T	GT*
Large	1183050	*VC1113*	A	G*
Large	1237259	*VC1165*	C	G*
Large	1237260	*VC1165*	G	C*
Large	1389484	*VC1307*	C	CC*
Large	1486534	*VC1395*	C	CC*
Large	2076300	*VC1925*	A	G*
Large	2153724	*VC2001*	T	A*
Large	2179341	*VC2027*	C	G*
Large	2358194	*VC2209*	C	CC*
Large	2379972	*VC2225*	A	G*
Large	2612864	*VC2437*	C	CC*
Large	2881400	*VC2710*	C	A*

Large	162660	*VC0165*	N	C*
Large	172531	*VC0172*	K	T*
Large	364992	*VC0343*	K	G*
Large	854836	*VC0798*	K	G*
Large	1011632	*VC0947*	W	A*
Large	1561424	*VC1451*	K	G*
Large	1561926	*VC1451*	S	C*
Large	1561942	*VC1451*	S	G*
Large	1561949	*VC1451*	W	A*
Large	1587146	*VC1482*	K	G*
Large	1587147	*VC1482*	Y	T*
Large	1587148	*VC1482*	Y	C*
Large	1587149	*VC1482*	Y	T*
Large	1587765	*VC1482*	W	T*
Large	1696639	*VC1585*	Y	C*
Large	1696645	*VC1585*	Y	C*
Large	2122955	*VC1971*	R	G*
Large	2122956	*VC1971*	M	C*
Large	2378839	*VC2224*	N	G*
Large	2590300	*VC2416*	R	A*
Large	2682957	*VC2497*	R	G*
Large	2682978	*VC2497*	S	G*
Large	764702	*between 16S and 23S*	K	G†
Large	57690	*23S*	Y	C†
Large	57714	*23S*	K	G†
Large	167458	*tRNA-pro*	R	G†
Large	328674	*23S*	Y	C†
Large	1670764	*between VC1558 and VC1559*	W	T†
Large	1735241	*VC1620(pseudogene)*	M	C†
Large	764715	*between 16S and 23S*	R§	
Large	2681238	*16S*	R§	
Large	2928036	*between 5S and 23S*	K§	
Large	2928147	*23S*	W§	
Small	17393	*VCA0015 (VCA0016)*	C	none
Small	53608	*VCA0044 (VCA0045)*	T	none
Small	57465	*VCA0049 (VCA0050)*	C	GC
Small	144626	*VCA0129*	A	none

Small	186168	*VCA0168 (VCA0169,0170)*	G	GG
Small	186754	*VCA0168 (VCA0169,0170)*	C	none
Small	186755	*VCA0168 (VCA0169,0170)*	C	none
Small	186759	*VCA0168 (VCA0169,0170)*	G	none
Small	186760	*VCA0168 (VCA0169,0170)*	G	none
Small	255374	*VCA0232*	G	GG
Small	277301	*VCA0257*	A	G
Small	279450	*VCA0260 (VCA0261)*	C	CC
Small	299055	*VCA0280*	G	none
Small	299080	*VCA0280*	C	none
Small	474527	*VCA0537*	C	CC
Small	535094	*VCA0596 (VCA0597)*	G	none
Small	686744	*VCA0740a*	T	CT
Small	969852	*VCA1022 (VCA1023)*	T	TT
Small	1036512	*VCA1082 (VCA1083)*	C	GC
Small	1062459	*VCA1106 (VCA1107)*	C	GC
Small	294983	*VCA0276*	G	GG*
Small	85485	*VCA0076*	T	G*
Small	152561	*VCA0137*	C	A*
Small	161478	*VCA0146*	C	G*
Small	472297	*VCA0534*	A	none*
Small	566512	*VCA0629*	C	G*
Small	566513	*VCA0629*	G	C*
Small	588148	*VCA0653*	C	G*
Small	609601	*VCA0674*	C	GC*
Small	631352	*VCA0692*	C	GC*
Small	631405	*VCA0692*	G	GG*
Small	690275	*VCA0745*	A	none*
Small	889858	*VCA0938*	T	GT*
Small	901045	*VCA0951*	C	T*
Small	980511	*VCA1031*	C	G*
Small	980512	*VCA1031*	G	C*
Small	980632	*VCA1031*	C	G*
Small	980633	*VCA1031*	G	C*
Small	980877	*VCA1031*	G	none*
Small	980887	*VCA1031*	T	GT*
Small	981512	*VCA1031*	A	none*
Small	993674	*VCA1043*	C	none*
Small	993684	*VCA1043*	C	A
Small	993700	*VCA1043*	C	none*
Small	993701	*VCA1043*	T	none*
Small	1004685	*VCA1055*	T	G*
Small	1045002	*VCA1089*	G	GG*

Small	1060870	*VCA1104*	T	CT*
Small	1060888	*VCA1104*	T	CT*
Small	646810	*VCA0705*	Y	T*
Small	366180	*VCA0387*	Y	C*
Small	413177	*VCA0464*	R	A*
Small	356433	*between VCA0368 and VCA0369*	Y¶	

* In each case the base has been corrected to that obtained from the N16961 ORF clone collection submitted by the Harvard Institute of Proteomics to the NCBI web site and confirmed by the unfinished MO10 genome.
† In each case the M66-2 and O395 base at the position are the same and consistent with the N16961 IUPAC base , and N16961 was corrected to the M66-2/O395 base.
§ For these 4 bases in the large chromosome, 2 are in a region absent in M66-2 and the other 2 are different at the position for both M66-2 and O395. However all are consistent with the N16961 IUPAC base. Because the 4 are in rrn regions, it is difficult to determine the corresponding region in other uncompleted Vibrio genomes, and the IUPAC codes were not changed. The ambiguity had no influence on our conclusions as SNPs in rrn regions were not used in SNP analysis.
¶ Base is in intergenic region (so no Harvard Institute clone data) in a N16961-specific region (relative to M66-2 and O395) of the integron, so can not be corrected. The ambiguity had no influence on our conclusions as SNPs in this region not scored.

others were corrected by resequencing.
